# Dietary intakes of flavan-3-ols and cardiovascular health: a field synopsis using evidence mapping of randomized trials and prospective cohort studies

**DOI:** 10.1186/s13643-018-0764-z

**Published:** 2018-07-18

**Authors:** Gowri Raman, Marissa Shams-White, Esther E. Avendano, Fan Chen, Janet A. Novotny, Aedin Cassidy

**Affiliations:** 1 Tufts Center for Clinical Evidence Synthesis, Institute for Clinical Research and Health Policy Studies, Tufts Medical Center, Box 63, 800 Washington Street, Boston, MA 02111 USA; 2 Tufts University Friedman School of Nutrition Science and Policy, 150 Harrison Ave, Boston, MA 02111 USA; 3 Agricultural Research Service, U.S. Department of Agriculture, Beltsville Human Nutrition Research Center, Beltsville, MD USA; 4 Department of Nutrition and Preventive Medicine, Norwich Medical School, University of East Anglia, Norwich, UK

**Keywords:** Cardiovascular disease, Diabetes, Lipids, Flavan-3-ols, Evidence mapping, Cocoa, Tea, Berries, Cinnamon, Red wine

## Abstract

**Background:**

There is considerable interest in the impact of increased flavan-3-ol intake on cardiovascular disease (CVD) and diabetes outcomes. Through evidence mapping, we determined the extent of the evidence base to initiate a future systematic review investigating the impact of flavan-3-ol intake on CVD and diabetes outcomes.

**Methods:**

We developed a research protocol, convened a technical expert panel (TEP) to refine the specific research questions, conducted a systematic search in multiple databases, double-screened abstracts and full-text articles, performed data extractions, and synthesized the data. We focused on randomized controlled trials (RCTs) and prospective cohort studies which assessed intakes of flavan-3-ol from foods, beverages, and supplement/extract sources on biomarkers and clinical outcomes of CVD and diabetes.

**Results:**

Of 257 eligible articles, 223 and 34 publications contributed to 226 RCTs and 39 prospective cohort studies, respectively. In RCTs, the most frequently studied interventions were cocoa-based products (23.2%); berries (16.1%); tea in the form of green tea (13.9%), black tea (7.2%), or unspecified tea (3.6%); and red wine (11.2%). Mean total flavan-3-ol intake was highest in the cocoa-based trials (618.7 mg/day) and lowest in the interventions feeding red wine (123.7 mg/day). The most frequently reported outcomes were intermediate biomarkers including serum lipid levels (63.4%), blood glucose (50.9%), blood pressure (50.8%), flow-mediated dilation (21.9%), and high-sensitivity C-reactive protein (21.9%). The included 34 prospective cohort studies predominantly examined exposures to flavan-3-ols (26%), cocoa-based products (23.2%), berries (16.1%), and green tea (13.9%) and CVD incidence and mortality.

**Conclusion:**

Through a systematic, evidence-based approach, evidence mapping on flavan-3-ol intake and CVD outcomes demonstrated sufficient data relating to flavan-3ol intake and biomarkers and clinical outcomes of CVD and diabetes. The current evidence base highlights the distribution of available data which both support the development of a future systematic review and identified the research need for future long-term RCTs.

**Systematic review registration:**

At present, evidence mapping is not eligible for registration on the international prospective register of systematic reviews (i.e., PROSPERO).

**Electronic supplementary material:**

The online version of this article (10.1186/s13643-018-0764-z) contains supplementary material, which is available to authorized users.

## Background

Dietary flavonoids represent a diverse range of polyphenolic compounds that occur naturally in plant foods. Their structural complexity has led to their sub-classification as flavonols, flavones, flavanones, flavan-3-ols (and their oligomers, pro(antho)cyanidins), isoflavones, and anthocyanins [1]. Although interest in the relationship between these bioactive constituents and cardiovascular disease (CVD) and diabetes risk is growing, their specific contribution to any health effects and the underlying mechanisms by which they act is an area of active research [2, 3].

To date, the sub-class that has received the most attention is flavan-3-ols, although most of the studies to date focus on two of the dietary sources, tea and chocolate. A number of meta-analyses have been published on the existing population-based evidence for these individual foods [1, 4–8]. Additionally, it is important to note that any health benefit from intake of tea or chocolate may be attributed to other bioactive constituents beyond flavan-3-ols and flavonoids, including other phenolics, caffeine, theobromine [9–11]. To date, in randomized controlled trials (RCTs) and prospective studies, although available findings on flavan-3-ols-rich foods are promising, dose-responses varied and the overall results are mixed. Potential adverse effects of flavan-3-ol extracts and supplements are also reported in literature [12, 13]. Studying a broad range of food sources rich in flavan-3-ols will help to further elucidate the potential health effects of flavan-3-ols per se to improve cardiometabolic health.

Evidence mapping is a tool used to systematically identify, organize, and summarize the quantity and focus of scientific evidence on a broad range of topics. Through evidence mapping, we determine the extent of the evidence base examining the impact of flavan-3-ol intake on CVD and diabetes outcomes and associated risk factors in RCTs and prospective cohort studies. The overall goal was to provide recommendations for future research and to identify if there is sufficient evidence to date to support initiating a full systematic review.

## Methods

### Description of evidence mapping

Evidence mapping is a tool to identify well-researched areas as well as gaps in evidence. It is often conducted to evaluate evidence on a broad topic before embarking on a full systematic review [14, 15]. Evidence mapping describes the characteristics of eligible literature typically in tabular forms and figures. Creating an evidence map usually involves the following steps: identifying a broad research area, defining key questions and variables and formulating a framework, engaging key stakeholders, conducting a systematic literature search, selecting studies for inclusion according to a priori eligibility criteria, and reporting descriptive results in tabular formats. In contrast to conducting a systematic review, evidence mapping does not necessitate detailed extraction, the assessment of risk of bias, and quantitative synthesis of results from eligible studies.

### Scope of the evidence map

Currently, there is no prescribed methodological or reporting standard (i.e., for evidence mapping) and, therefore, we followed a series of logical steps in creating this evidence map. First, we assembled a review team and developed a protocol; identified and convened a technical expert panel (TEP) with content expertise, methodological expertise, or both; refined specific research questions with the input of the TEP; and conducted a systematic search in multiple databases. We then double-screened abstracts and full-text articles, performed data extractions, and synthesized data in tabular forms. The data was used to identify areas within the topic of flavan-3-ols and cardiovascular outcomes with a paucity of research that are ideal focuses for future studies, as well as well-researched topics where there is sufficient evidence to warrant a full systematic review.

### Review team and development of a protocol

**Fig. 1:**
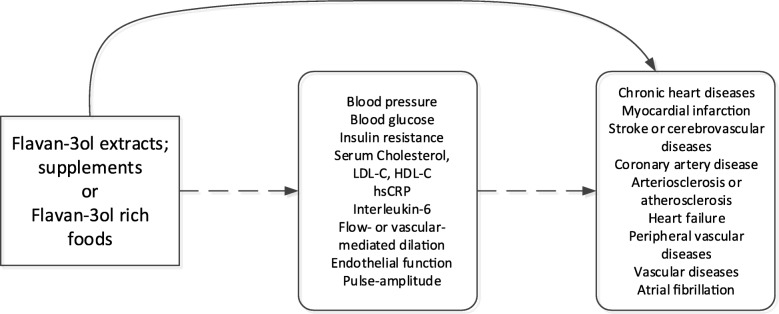
Analytic framework for evaluating the association between flavan-3-ol and outcomes. Legend: Analytic framework displays the potential association pathway between flavan-3-ol and outcomes of interest

Our review team was comprised of two nutrition researchers with expertise in flavan-3-ols, one methodologist, and three research assistants with an interest and training in nutrition research. First, we identified preliminary key questions and study eligibility criteria through discussions of Populations, Interventions/Exposures, Comparators, Outcomes, and Study designs or Settings (PI(E)COS) of interest and developed the analytic framework (Fig. 1). Next, we utilized the United States Department of Agriculture (USDA) monomer and procyanidin databases or Phenol-Explorer databases to identify commonly consumed foods and beverages that have the highest flavan-3-ol content—either as monomers or polymers—and assembled a comprehensive list of them in an Excel™ spreadsheet [16–18]. We did not search for foods that have low flavan-3-ol content but are consumed at a high frequency. A detailed search strategy and protocol was then developed outlining key questions and the scope of the review. This review was submitted to the International Life Sciences Institute (ILSI) bioactive committee for their input, but we retained the independence to decide whether or not to incorporate any suggestions received from the committee.

### Technical expert panel

Our review team identified and convened a multidisciplinary TEP, which included four flavan-3-ol researchers from the USDA, academic centers in North America and the UK; two methodologists with expertise in nutrition research; and a cardiologist. The TEP provided input to help refine the questions and protocol, identify important issues, and define parameters for the mapping of evidence. The review team also asked the TEP to suggest additional search strategy terms. See Additional file 1: Table S1 for a list of TEP members and affiliations.

### Search strategy

A comprehensive search of the scientific literature was conducted in MEDLINE®, the Cochrane Central Trials Registry®, and Commonwealth Agricultural Bureau (CAB) from inception through April 26, 2016. The reference lists of any previously published systematic reviews on flavonoids and health identified by the review team or TEP were hand-searched to identify any additional studies not captured by the database searches.

To capture interventions of interest, we combined terms of monomer and proanthocyanidins for flavan-3-ols, foods, beverages, and spices (e.g., cinnamon) rich in flavan-3-ols with the outcomes of interest including any cardiovascular outcomes and intermediate biomarkers, inflammatory markers, and endothelial function. The search strategy included terms for the study designs of interest (i.e., randomized trials and prospective cohort studies) and was limited to those conducted in humans (see Additional file 1: Table S2 for the complete search strategy). No language restriction was applied during the search process.

### Study selection

Our review team used an iterative process to establish study eligibility criteria based on the aforementioned PI(E)COS, detailed in Tables 1 and 2. Using these criteria, we assessed abstracts and/or titles of citations identified from literature searches for inclusion, retrieved full-text articles of potentially relevant citations, and re-assessed the articles for inclusion. Articles published in the English language were selected. Both abstract and full-text screenings were conducted in duplicate, with conflicts resolved by reconciliation with the whole research team in weekly group meetings. The project lead reconfirmed all rejected full-text articles. Additionally, content experts were consulted through emails for questions that arose in weekly group meetings.

**Table 1:** Inclusion and exclusion criteria

Inclusion criteria	Exclusion criteria
Populations: humans only	Populations: those including:
Adults 18+ years of age	Non-human subjects (e.g., in vitro, cell, stem cell, or animal studies)
Healthy adults	
Adults at an increased risk for cardiovascular disease (CVD)	Children and adolescents (< 18 years old)
Adults who had existing CVD	Pregnant or lactating women
Cancer survivors who were cancer-free at the time of the study	Adults with prior organ transplantation
All elderly *unless* clear disease (e.g., renal disease)	Adults with chronic, inflammatory, or autoimmune disease conditions (e.g., rheumatoid arthritis, polycystic ovarian syndrome, chronic kidney disease)
	100% smokers
	Participants in concurrent cancer or exercise trials
	Studies with < 10 subjects
Interventions:	Interventions:
Foods, beverages, supplements, or extracts that described or defined flavan-3-ol content either as monomers or polymers	Did not evaluate a specific intervention (e.g., spice blend, dietary patterns rich in flavan-3-ol food sources)
	Foods with no or low flavan-3-ol content (e.g., white chocolate, raisins)
Example foods: fruits (e.g., apples, berries, grapes), dark chocolate, cocoa, teas, red wine)	
	Foods with insufficient data to allow a reasonable estimation of flavan-3-ol content using standardized databases [16–18]
Studies must quantify flavan-3-ols consumed or provide sufficient data that allowed estimation of the flavan-3-ol content via standardized databases^1^ [16–18]	Did not specify the type of wine (e.g., white, red, rice), alcohol, or chocolate (e.g., dark, white, or milk chocolate) in their questionnaires and/or reported results
Comparators of interest:	Comparators of interest:
Low or no flavan-3-ol content, including placebos	Studies that did not differentiate flavan-3-ol content (e.g., a study evaluating the effects of alcohol comparing red wine vs. de-alcoholized red wine)
Outcomes of interest:	Outcomes of interest:
Key clinical and intermediate outcomes (Table 2)^2^	Clinical and intermediate outcomes of interest (Table 2) that do not meet the minimum follow-up requirements
Study designs of interest:	Study designs of interest:
Randomized controlled trials (RCTs)	Cross-sectional studies
Prospective cohort studies	Case reports
	Case series
	Retrospective studies
	Systematic reviews and meta-analyses^3^
	Reviews, lectures, opinion articles, news articles, proposed studies (i.e., no results reported)

^1^For all interventions of interest when flavan-3-ols were not fully reported in an article, we utilized the recent USDA monomer and procyanidin databases or Phenol-Explorer databases to estimate flavan-3-ol content–either as monomers or procyanidin based on the intake values reported in each article.

^2^When outcomes were reported at multiple time points, only baseline and final follow-up results were extracted

^3^Though systematic reviews and meta-analyses were excluded during the screening process, their references were screened for any additional, relevant studies

**Table 2:** Included outcomes of interest and minimum follow-up cutoffs

○ Cardiovascular disease (CVD) clinical outcomes (≥ 6 months follow-up), including acute coronary syndrome, angina, arteriosclerosis, atherosclerosis, atrial fibrillation, cerebrovascular disorders, coronary artery disease, heart failure, myocardial infarction, peripheral vascular disease, and venous thromboembolism^1^ ○ Serum lipids (≥ 3 weeks follow-up),including blood pressure (BP), diastolic BP (DBP), low-density lipoprotein (LDL), high-density lipoprotein (HDL), systolic BP (SBP), total cholesterol, triglycerides ○ Inflammatory markers (≥ 3 weeks follow-up), including high-sensitivity C-reactive protein (hs-CRP), intercellular adhesion molecule 1 (ICAM-1), interleukins, vascular cell adhesion molecule 1 (VCAM-1) ○ Metabolic parameters (≥ 1 week follow-up), including blood glucose, diabetes, hemoglobin A1C (HbA1C), homeostatic model assessment and insulin resistance (HOMA-IR), insulin resistance, quantitative insulin sensitivity check index (QUICKI) ○ Evaluation of endothelial function (any study length, including acute studies), using Endo-PAT, flow-mediated dilation (FMD), pulse-amplitude, pulse wave analysis, pulse wave velocity	

^1^When outcomes were reported at multiple time points, the results reported at baseline and the final follow-up were extracted

### Data extraction

In discussion with nutrition experts within the review team, data was extracted into customized forms in the Systematic Review Data Repository (SRDR) online system (https://srdr.ahrq.gov). The forms were designed to capture all elements relevant to the descriptions on study population characteristics, enrolled and analyzed sample sizes, study design features, interventions and comparators, and relevant outcomes. If flavan-3-ol levels were not explicitly reported in the paper, where possible, data extractors estimated flavan-3-ol intake levels using either the USDA monomer and proanthocyanidins or Phenol-Explorer databases [16–18]. We also extracted qualitative outcomes (i.e., direction of association and statistical significance). The forms were piloted on several studies, revised, and optimized as necessary by the project lead. One team member extracted each study’s data. A second team member confirmed interventions and outcome data entries at the time of data analysis.

### Data synthesis

All included studies were summarized in narrative form and in summary tables that tabulated the important features of the study populations, designs, interventions, outcomes, and results. Studies were also summarized qualitatively in tabular form and graphically using heat maps.

## Results

**Fig. 2:**
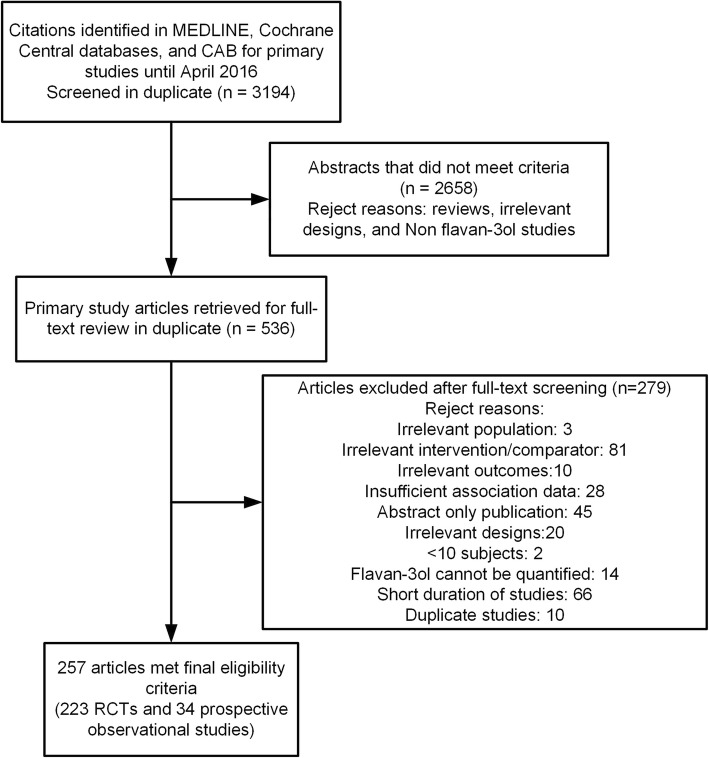
Study Flow Diagram. Legend: Abstracts identified (*n* = 3194); Abstracts not meeting criteria (*n* = 2658); Full-text articles excluded after screening (*n* = 279); Full-text articles meeting study eligibility criteria (*n* = 257); Eligible randomized controlled trials (*n* = 223) and prospective observational studies (*n* = 34). RCTs = randomized controlled trials

We identified 3194 abstracts examining flavan-3-ol intakes and cardiovascular outcomes (including MI, stroke) and a range of CVD risk factors. Of these, 536 (16.8%) abstracts met the broad eligibility criteria and were retrieved and re-examined as full-text articles; 257 of the full-text articles (223 RCTs and 34 prospective studies) were eligible for data entry into the final database (Fig. 2). Of the 257 articles, 223 publications contributed to 226 RCTs and 34 publications contributed to 39 prospective observational cohort studies (i.e., some articles reported multiple studies), and eligible studies are listed in the Additional file 1.

### Description of RCTs

**Fig. 3:**
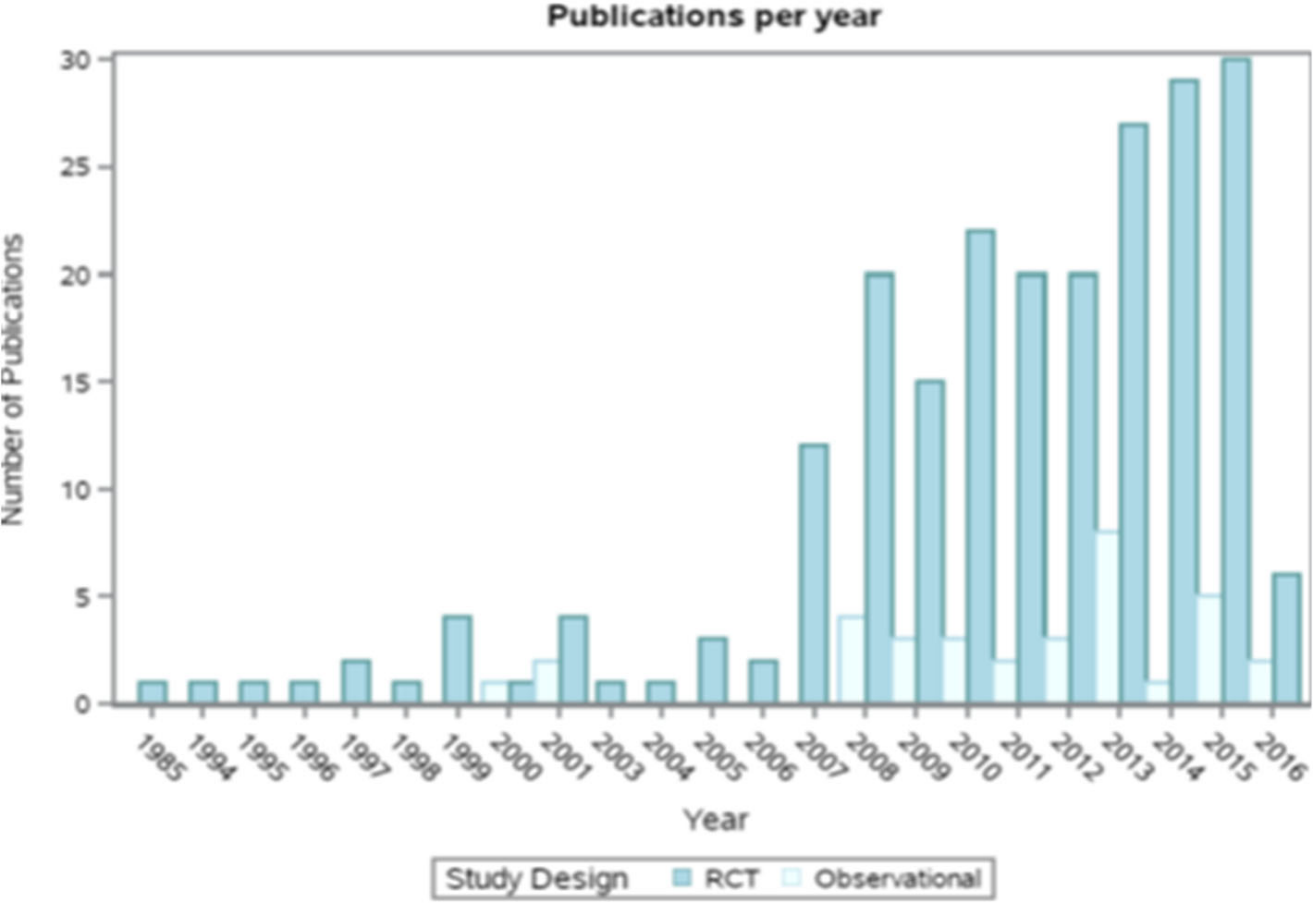
Bar graph displaying the mean number of RCT and observational study flavan-3-ols articles published per year between 1985 and 2016. RCT = randomized controlled trial

The 223 articles were published between March 1985 and April 2016 and contributed to 226 RCTs in the database (i.e., three publications contained two RCTs each) (Fig. 3). The top three regions where the RCTs were conducted were Western Europe (38.6%), North America (26.5%), and Asia (13.0%). The majority of RCTs were parallel study designs (62.3%), and the remaining were (37.7%) crossover studies. Regarding blinding, 55.6% of the RCTs were double-blinded, 17.9% were single-blinded, 11.2% were not blinded, and 15.3% did not explicitly report on blinding. Over half of the RCTs were conducted among adults ≥ 50 years of age (56.1%); 57.0% included predominantly metabolically at-risk adults; 29.2% included healthy adults (i.e., adults without any risk factors for CVD or without existing CVD); 4.9% included adults with existing CVD; 4.9% included adults with more than one of the aforementioned categories; and 4.0% included adults with other conditions at baseline.

#### Included interventions of interest

The most frequently studied intervention in RCTs was cocoa-based products (23.2%), consumed as dark chocolate, cocoa, or added cocoa polyphenols. The second most frequently studied intervention was tea in the form of green tea (13.9%), black tea (7.2%), or unspecified tea (3.6%), followed by berries and red wine in 16.1 and 11.2% of the RCTs, respectively. Less than 10% of the RCTs included other interventions such as apples, cinnamon, cranberries, flavan-3-ol monomer or proanthocyanidin supplementation, grapes, grape seed, plums, and mixed interventions of interest. Table 3 illustrates the evaluation of these interventions.

**Table 3:** Descriptive characteristics of eligible RCTs

	Total *N* (%)	RCT parallel *N* (%)	RCT crossover *N* (%)
*N* publications	223 (100.00)	139 (62.33)	84 (37.67)
Blinding			
Double-blind	124 (55.61)	87 (62.59)	37 (44.05)
Single-blind	40 (17.94)	19 (13.67)	21 (25.00)
Not blinded	25 (11.21)	14 (10.07)	11 (13.10)
Not reported	34 (15.25)	19 (13.67)	15 (17.86)
Mean age			
< 50 years	98 (43.95)	57 (41.01)	41 (48.81)
≥ 50 years	125 (56.05)	82 (58.99)	43 (51.19)
Baseline health			
Healthy	65 (29.15)	29 (20.86)	36 (42.86)
At risk for CVD	127 (56.95)	91 (65.47)	36 (42.86)
Existing CVD	11 (4.93)	5 (3.60)	6 (7.14)
Mixed	11 (4.93)	6 (4.32)	5 (5.95)
Other	9 (4.04)	8 (5.76)	1 (1.19)
Study region			
Africa	2 (0.90)	0 (0.00)	2 (2.38)
Asia	29 (13.00)	28 (20.14)	1 (1.19)
Australia	12 (5.38)	6 (4.32)	6 (7.14)
Eastern Europe	5 (2.24)	5 (3.60)	0 (0.00)
Western Europe	86 (38.57)	38 (27.34)	48 (57.14)
Middle East	24 (10.76)	23 (16.55)	1 (1.19)
North America	59 (26.46)	34 (24.46)	25 (29.76)
South America	5 (2.24)	4 (2.88)	1 (1.19)
Mixed	1 (0.45)	1 (0.72)	0 (0.00)
Intervention			
Apple	4 (1.79)	1 (0.72)	3 (3.57)
Berries	36 (16.14)	24 (17.27)	12 (14.29)
Black tea	16 (7.17)	9 (6.47)	7 (8.33)
Chocolate	52 (23.21)	27 (19.42)	25 (29.76)
Cinnamon	18 (8.07)	17 (12.23)	1 (1.19)
monomers	9 (4.04)	6 (4.32)	3 (3.57)
polymers	1 (0.45)	1 (0.72)	0 (0.00)
Grape	7 (3.14)	4 (2.88)	3 (3.57)
Grape seed	13 (5.83)	9 (6.47)	4 (4.57)
Green tea	31 (13.90)	26 (18.71)	5 (5.95)
Mixed/other	3 (1.35)	3 (2.16)	0 (0.00)
Tea	8 (3.59)	4 (2.88)	4 (4.76)
Wine	25 (11.21)	8 (5.76)	17 (20.24)

*CVD* cardiovascular disease, *N* number, *RCT* randomized controlled trial

Mean total flavan-3-ol intake varied across interventions. It was highest for RCTs administering chocolate (618.7 mg/day), green tea (473.4 mg/day) or black tea (256.2 mg/day), apples (444.1 mg/day), flavan-3-ol monomers (350.7 mg/day), berries (250 mg/day), and red wine (123.7 mg/day). Across interventions, the average study duration ranged between 4 and 17 weeks. A detailed description of reported or estimated flavan-3-ols is tabulated in the Additional file 1.

#### Outcomes of interest

**Fig. 4:**
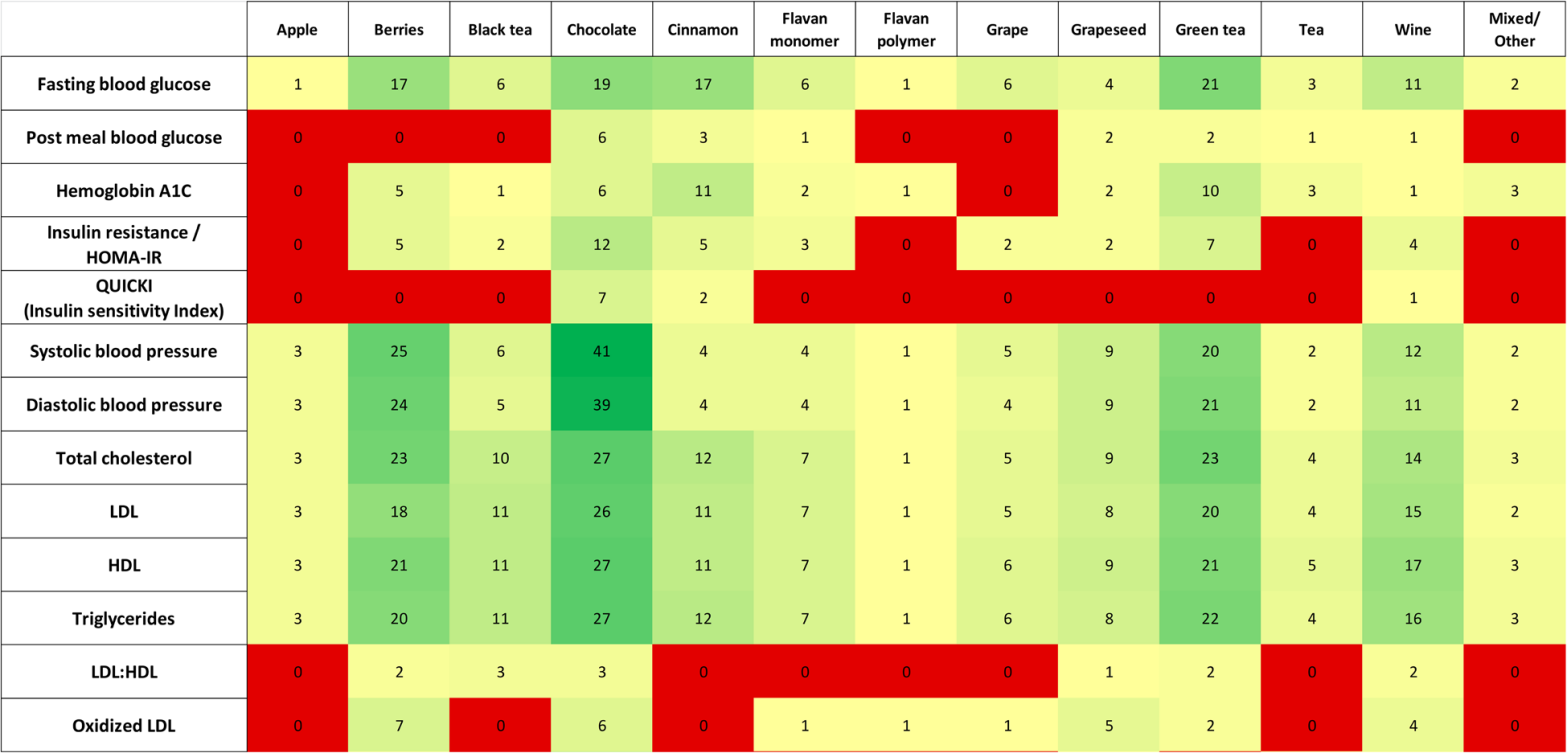
Heat map showing the distribution of RCTs by their intervention arms and most commonly evaluated outcomes. RCTs = randomized controlled trials

**Fig. 5:**
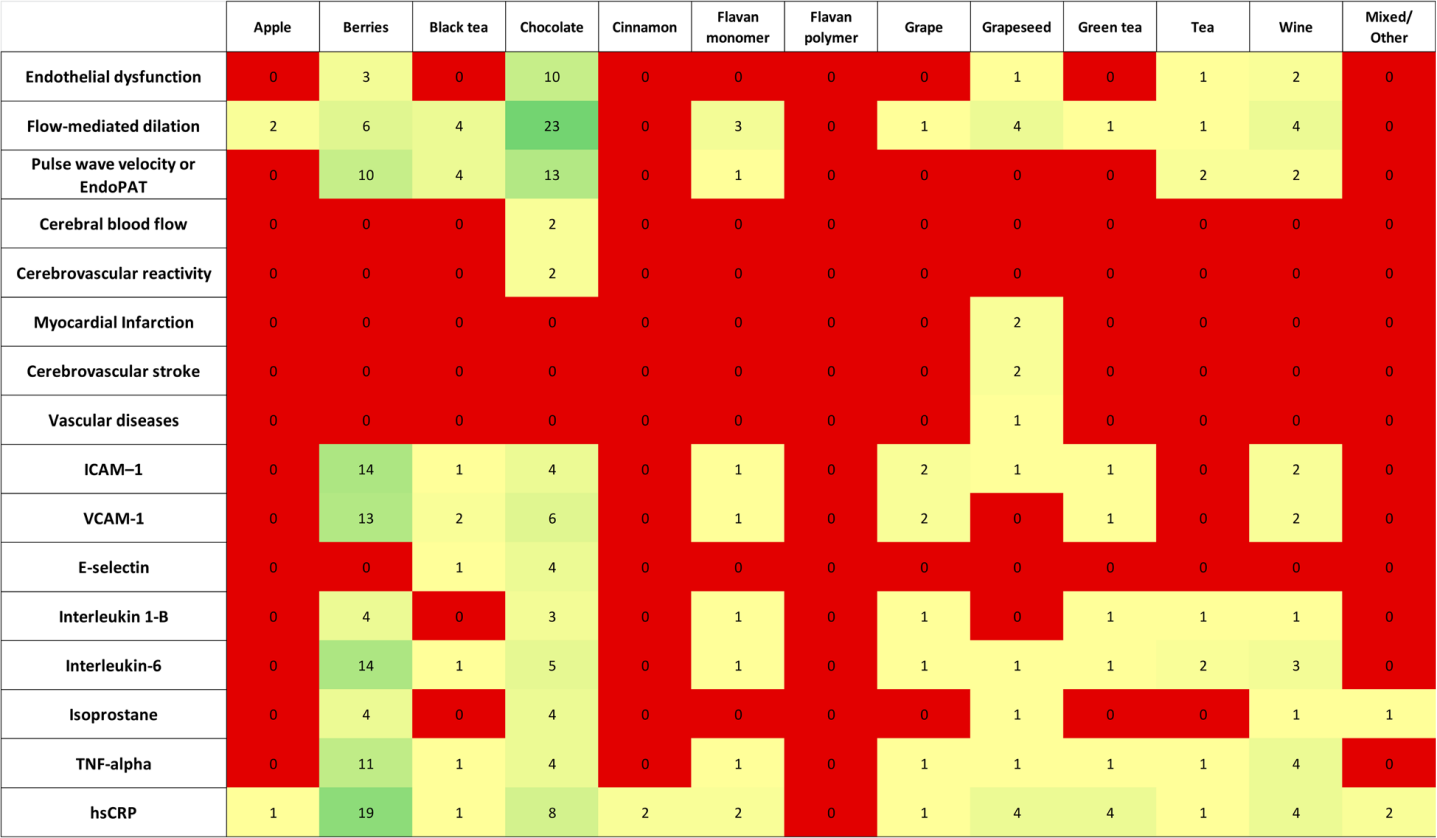
Heat map showing the distribution of RCTs by their intervention arms and less frequently evaluated outcomes. RCTs = randomized controlled trials

The most frequently reported outcomes in RCTs of berries, cocoa-based products, green tea, and wine were blood glucose (50.9%), blood pressure (BP) (50.8%), serum lipid levels (63.4%), flow-mediated dilation (FMD) (21.9%), and high-sensitivity C-reactive protein (hs-CRP) (21.9%) (Figs. 4 and 5). In addition, cinnamon trials frequently reported blood glucose and blood lipid outcomes, grape seed trials reported FMD and hs-CRP, and cranberry trials reported hs-CRP. The sample sizes per trial ranged from 42 to 70 subjects. Though trials overall were more likely to report improvements in evaluated outcomes after the intervention as compared with the control (Additional file 1), within each group (compared to baseline) statistical significance was reached in < 50% of the studies and between groups (intervention vs. control) statistical significance was reached in < 25% of the studies.

Descriptive data on additional outcomes included insulin resistance or homeostatic model assessment and insulin resistance (HOMA-IR) (18.9%), oxidized low-density lipoprotein (LDL) (12.1%), pulse-wave velocity (14.3%), intercellular adhesion molecule 1 (ICAM-1) (11.6%), vascular cell adhesion molecule 1 (VCAM-1) (12.1%), interleukin-6 (12.9%), tumor necrosis factor alpha (TNF-alpha) (11.2%), and other outcomes (< 10%).

### Description of cohort studies

The 39 prospective cohort studies (in 34 publications) represented 47 adjusted models. The cohort studies were conducted in Europe (16 studies), the USA (10 studies), Asia (7 studies), and Australia (1 study). The exposures examined included total dietary flavan-3-ol intake (9 models; 37,382 subjects); berries (6 models; 54,780 subjects); apples (5 models; 53,383 subjects); green tea (5 models; 25,420 subjects); cocoa-based products (4 models; 27,028 subjects); black tea (3 models; 42,699 subjects); monomer intake (3 models; 24,746 subjects); grape and raisins (3 models; 62,461 subjects); plums (3 models; 27,365 subjects); red wine (3 models; 5434 subjects); unspecified type of tea (2 models; 5399 subjects); and proanthocyanidin intake (1 model; 40,574 subjects). Across cohort studies, the average study duration ranged between 4.5 and 24 years. The associated cardiovascular outcomes examined included cardiovascular mortality, cerebrovascular stroke, and BP.

## Discussion

This evidence mapping on flavan-3-ol intakes identified that cocoa-based products, tea, berries, and red wine each have several published RCTs examining relevant CVD and diabetes outcomes of interest. Additionally, aside from an overall focus on total flavan-3-ol intake, berries, apples, green tea, and cocoa-based products were the predominant, habitual dietary sources.

### Description of existing literature

A number of meta-analyses have been published on the existing population-based evidence for some of these foods individually, but none has been conducted evaluating a broad range of topics with a specific focus toward total flavan-3-ol intake [4, 6]. A previous systematic review on chocolate, which included six cohort studies and one cross-sectional study, showed that higher intake was associated with a 37% (95% CI 10–46%) reduction in risk for CVD and 29% (2–48%) reduction in risk for stroke, compared with lower intake [4]. Largely based on the same studies, three other meta-analyses of population-based data reported relative risk (RR) reductions of between 13 and 25% for stroke [6] in the highest (median 62.9 g/week) versus the lowest (0 g/week) intake of chocolate. For tea, a recent meta-analysis of 22 prospective studies reported that a 3 cup/day increase in consumption was associated with a reduction in risk of incident heart disease (RR, 0.73; 95% CI 0.53–0.99), incident stroke (RR, 0.82; 95% CI 0.73–0.92), and total mortality (RR, 0.76; 95% CI 0.63–0.91), but had no significant effect on stroke mortality (RR, 0.93; 95% CI 0.83–1.05) [8].

Three meta-analyses of RCTs [7, 19, 20] that reviewed the impact of cocoa/chocolate consumption on cardiometabolic health from the available short duration (≤ 18 weeks) studies reported similar effects, despite minor differences in inclusion criteria in two RCTs [7, 19]. One meta-analysis reported a − 2.77 mmHg (95% CI − 4.72, − 0.82 mmHg, *p* = 0.005) decrease in systolic BP and − 2.20 mmHg (95% CI − 3.46, − 0.93 mmHg, *p* = 0.006) reduction in diastolic BP following cocoa intake (17). The other systematic reviews, which had minor differences in inclusion criteria, reported similar effects [7, 19]. The magnitude of associations are clinically relevant and of significant public health importance. For tea, one meta-analysis of 11 short-term RCTs (duration 1–26 weeks) observed reductions in systolic BP and diastolic BP [5], while another meta-analysis of 10 studies suggested that consumption of black tea significantly reduced LDL cholesterol concentrations but did not alter total or high density lipoprotein (HDL) cholesterol levels [21].

In relation to the specific associations between habitual intakes of flavan-3-ols and CVD, the most recent review of prospective cohort studies reported no association with coronary heart disease (CHD) mortality, CHD incidence, and stroke risk [22]. However, in one study, higher flavan-3-ol intake (monomers only) was associated with a 17% decrease in CVD mortality [23]. Two other studies, which included both monomers and other pro(antho)cyanidins, also reported a 66% decrease in atherosclerotic vascular disease mortality [24] and a 60% decrease in CVD events and mortality [25]. In relation to procyanidin intake alone, a higher intake was associated with a 10–13% decrease in CVD mortality in two studies [23, 26] and a 17% decrease in incident and fatal CHD in one study [27].

In relation to RCTs, to date, three systematic reviews have examined the potential effective dose of flavan-3-ols from the short-term trials on tea and cocoa. One observed a non-linear dose-response effect with a maximal effect observed at a “total polyphenol intake” of 500 mg [7]. Another suggested that intakes of epicatechin > 50 mg resulted in greater effects on systolic BP and diastolic BP, while FMD improved at all levels of epicatechin intake [1]. The most recent systematic review suggested that the acute and chronic FMD and BP responses for epicatechin, catechin, or procyanidin intakes were marginally greater for these individual constituents compared to total flavan-3-ol intake [28]. Furthermore, while there was no indication of a dose-response for BP, there were non-linear dose-response relationships observed for FMD, with a greater acute response observed at lower intake levels (< 1 g/day procyanidins, < 200 mg/day monomers) and a U-shaped response observed following chronic ingestion [28]. Several flavan-3-ol studies have also been conducted focusing on feeding chemically “pure” (-) – epicatechin to participants. Two recent studies administered 100 mg/day of epicatechin to participants for 4 weeks. One reported no change in FMD or BP but did report improvements in insulin resistance [29], while the second also reported favorable effects on insulin resistance and improvements in both inflammatory and lipid biomarkers [30].

### Role and value of evidence mapping

There is an increasing interest in these types of evidence mapping methods to create databases for identification of gaps or topics for future reviews [31, 32]. At the outset, we identified two experts from the flavan-3-ol research community and consulted with them throughout the review process. These experts assisted with the preliminary refinement of the PI(E)COS definitions, the analytic framework, and the review of evidence mapping results, and confirmed that the evidence mapping was thorough and comprehensive.

Prior to the review of abstracts and full-text articles, the review team received training on the screening process about how to identify relevant abstracts and articles using the pre-specified eligibility criteria. Key steps of this evidence mapping included (1) the determination of appropriate clinical and biochemical markers as outcomes of interest; (2) the use of appropriate duration of follow-up to assess each of the outcomes; (3) the review of abstracts and full-text articles for relevance from a broad range of topics; (4) the estimation of the flavan-3-ol content from relevant databases when they were not reported in individual articles; and (5) recommending whether there is sufficient evidence to evaluate the impact of intake of the flavan-3-ol content on cardiovascular health in a systematic review. An evidence map using pre-specified eligibility criteria is a robust and effective method to determine whether there is sufficient evidence on clinical outcomes, intermediate outcomes, or dose-response data to allow a formal re-examination of a particular broad topic.

The results of this evidence map suggest that there are sufficient data available to conduct a systematic review and meta-analysis of flavan-3-ol intake and CVD outcomes. Moreover, additional studies may have been completed since our last search date (April 2016). Most of the included RCTs reported the presence or absence of blinding and had few dropouts (enrolled vs. analyzed). Additionally, the body of evidence from RCTs and cohort studies examining flavan-3-ol interventions included a sufficient number of outcomes of interest to justify a future systematic review and meta-analyses. There will be challenges comparing different flavan-3-ol sources in a systematic review or meta-analysis as they will likely differ considerably in composition, bioaccessibility, and in the presence of other bioactive compounds. Flavan-3-ols from different sources may also vary in relation to both bioavailability and bioactivity. Nonetheless, the evidence from a full systematic review would bring credible weight to deliberations about the relative importance of flavan-3-ols per se (irrespective of source); may help guide authoritative bodies, such as the Institute of Medicine (IOM) on recommended intakes; and may assist the Dietary Guidelines Advisory Committee for 2020 guidelines. Currently, we are embarking on a full systematic review on this topic, and the literature search will be updated for additional studies published since our last search date.

## Conclusion

Our evidence mapping on flavan-3-ol intake and CVD uses a systematic, evidence-based approach to search for, identify, collect data on, and evaluate relevant studies examining the effects of foods, beverages, and supplements/extracts high in flavan-3-ols. This study on flavan-3-ol intake and CVD outcomes demonstrated that there is sufficient data relating to flavan-3-ol intake and biomarkers and clinical outcomes of CVD and diabetes to potentially conduct a systematic review and meta-analysis. It also highlighted as an evidence gap a lack of long-term RCTs. Additional gaps in evidence included fewer trials evaluating fruits such as apple, plums, and grapes; fewer trials conducted among healthy adults compared to at-risk populations; and except for green tea and cinnamon, fewer trials from regions other than Western Europe. The current evidence base highlights the distribution of available data that support the development of a future systematic review and meta-analysis. Additionally, the identification of evidence gaps can help prioritize and identify the key future research needs.

## Additional file

Additional file 1: **Table S1.** List of Technical expert panel members. **Table S2.** Search strategy. **Table S3.** The most frequently reported outcomes in evaluated RCTs. **Table S4.** Description of cohort studies by intervention. **Table S5.** Description of all RCTs by intervention. **Table S6.** Flavan-3ol content by intervention. **Table S7.** Description of outcomes reported in apple intervention studies. **Table S8.** Description of outcomes reported in berries intervention studies. **Table S9.** Description of outcomes reported in black tea intervention studies. **Table S10.** Description of outcomes reported in chocolate intervention studies. **Table S11.** Description of outcomes reported in cinnamon intervention studies. **Table S12.** Description of outcomes reported in cranberry intervention studies. **Table S13.** Description of outcomes reported in flavan monomer intervention studies. **Table S14.** Description of outcomes reported in flavan polymer intervention studies. **Table S15.** Description of outcomes reported in grape intervention studies. **Table S16.** Description of outcomes reported in grapeseed intervention studies. Table **S17.** Description of outcomes reported in green tea intervention studies. **Table S18.** Description of outcomes reported in mixed/other intervention studies. **Table S19.** Description of outcomes reported in plums intervention studies. **Table S20.** Description of outcomes reported in tea intervention studies. **Table S21.** Description of outcomes reported in wine intervention studies. (DOCX 245 kb)

## Abbreviations

(PI(E)COS): Populations, Interventions/Exposures, Comparators, Outcomes, and Study designs or Settings
BP: Blood pressure
CAB: Commonwealth Agricultural Bureau
CHD: Coronary heart disease
CI: Confidence interval
CVD: Cardiovascular disease
FMD: Flow-mediated dilation
HDL: High-density lipoprotein
HOMA-IR: Homeostatic model assessment-insulin resistance
hs-CRP: High-sensitivity C-reactive protein
ICAM-1: Intercellular Adhesion Molecule 1
ILSI: International Life Sciences Institute
IOM: Institute of Medicine
LDL: Low-density lipoprotein
MI: Myocardial infarction
RCTs: Randomized controlled trials
RR: Relative risk
SRDR: Systematic Review Data Repository
TEP: Technical expert panel
TNF-alpha: Tumor necrosis factor alpha
US: United States
USDA: United States Department of Agriculture
VCAM-1: Vascular cell adhesion molecule 1
