# Supplementary material for: Dietary intakes of flavan-3-ols and cardiovascular health: a field synopsis using evidence mapping of randomized trials and prospective cohort studies
Source: Syst Rev. 2018 Jul 18;7:100. doi: 10.1186/s13643-018-0764-z (PMC6052707; doi:10.1186/s13643-018-0764-z)
Supplement: Supplementary file 1 — Table S1. List of Technical expert panel members. Table S2. Search strategy. Table S3. The most frequently reported outcomes in evaluated RCTs. Table S4. Description of cohort studies by intervention. Table S5. Description of all RCTs by intervention. Table S6. Flavan-3ol content by intervention. Table S7. Description of outcomes reported in apple intervention studies. Table S8. Description of outcomes reported in berries intervention studies. Table S9. Description of outcomes reported in black tea intervention studies. Table S10. Description of outcomes reported in chocolate intervention studies. Table S11. Description of outcomes reported in cinnamon intervention studies. Table S12. Description of outcomes reported in cranberry intervention studies. Table S13. Description of outcomes reported in flavan monomer intervention studies. Table S14. Description of outcomes reported in flavan polymer intervention studies. Table S15. Description of outcomes reported in grape intervention studies. Table S16. Description of outcomes reported in grapeseed intervention studies. Table S17. Description of outcomes reported in green tea intervention studies. Table S18. Description of outcomes reported in mixed/other intervention studies. Table S19. Description of outcomes reported in plums intervention studies. Table S20. Description of outcomes reported in tea intervention studies. Table S21. Description of outcomes reported in wine intervention studies. (DOCX 245 kb) [file 13643_2018_764_MOESM1_ESM.docx]

Table S1 List of Technical Expert Panel members

| **Technical Expert** | **Affiliation** |
| --- | --- |
| **Nutrition with expertise/interest in Flavan-3ol** | |
| Chung-Yen (Oliver) Chen, Ph.D | Jean Mayer USDA Human Nutrition Center on Aging |
| Emilie Combet, Ph.D. | University of Glasgow |
| Mario Ferruzi, Ph.D. | Purdue University |
| Colin Kay, Ph. D. | Norwich Medical School, University of East Anglia |
| Joanne Spahn, M.S., R.D., F.A.D.A. | USDA Center for Nutrition Policy and Promotion |
| **Methodologist** | |
| Joseph Lau, M.D. | Brown University |
| **Cardiologist** | |
| Benjamin Wessler, M.D. | Tufts Medical Center |

Table S2 Search Strategy

| **Searches** | **Results** |
| --- | --- |
| 1 | (proanthocyanidin$ or pro-anthocyanidin$ or procyanidin$ or pro-cyanidin$).mp. |
| 2 | (catechin$ or epicatechin$ or epi-catechin$).mp. |
| 3 | (Flavanol$ or catechin$ or epicatechin$ or epigallocatechin$ or gallocatechin$ or epigallocatechin$ or procyanidin$ or proanthocyanidin$).mp. |
| 4 | (flavan-3-ol$ or 3-flavan-ol or theaflavin or theaflavin 3-3'-digallate or theaflavin 3'-gallate or theaflavin 3-gallate or theaflavin 3-3-digallate or thearubigin).mp. |
| 5 | expcatechin/ or expproanthocyanidins/ |
| 6 | (condensed and tannin$).mp. |
| 7 | or/1-6 |
| 8 | (chocolate$ or cocoa$ or cacao$).mp. |
| 9 | Cacao/ |
| 10 | exp Camellia sinensis/ or exp Camellia sinenses/ or expTheasinenses/ or expTheasinensis/ |
| 11 | exp tea/ or tea.mp. or (black adj1 tea).tw. or (green adj1 tea).tw. or (black and tea and extract).tw. or (green and tea and extract).tw. |
| 12 | exp wine/ or wine.mp. |
| 13 | expvitis/ or vitis.mp. or (grape or grapes).mp. or (grape and juice).mp. |
| 14 | expcinnamomumzeylanicum/ or (cinnamomum and zeylanicum).mp. or cinnamon.tw. |
| 15 | expmalus/ or malus.mp. or apple$.tw. or (apple and juice).tw. |
| 16 | exp blueberry plant/ or blueberr$.mp. |
| 17 | cranberr$.mp. |
| 18 | expvaccinium/ or vaccinium.mp. |
| 19 | (strawberr$ or raspberr$ or blackberr$ or chokeberr$ or currant$ or ribes).mp. |
| 20 | expfragaria/ or fragaria.mp. |
| 21 | exppyrus/ or pyrus.af. |
| 22 | (pear or pears or plum or plums or cherry or cherries or nectarine or nectarines).mp. |
| 23 | expprunus/ or prunus.mp. |
| 24 | exprubus/ or rubus.mp. |
| 25 | or/8-24 |
| 26 | 7 or 25 |
| 27 | exp cardiovascular diseases/ or cardiovascular.mp. |
| 28 | exp heart diseases/ or heart.mp. |
| 29 | exp myocardial infarction/ or myocardial infaction.mp. |
| 30 | exp stroke/ or stroke.mp. |
| 31 | exp cerebrovascular disorders/ or cerebrovascular.mp. |
| 32 | exp coronary artery disease/ or exp coronary heart disease/ or coronary.mp. |
| 33 | (heart attack$ or sudden death$).tw. |
| 34 | (arteriosclerosis or atherosclerosis).mp. |
| 35 | exp heart failure/ or heart failure.mp. |
| 36 | exp peripheral vascular diseases/ or (peripheral and vascular and diseases).mp. |
| 37 | (Blood pressure or hypertension).mp. or exp hypertension/ |
| 38 | exp diabetes mellitus/ or diabetes.mp. |
| 39 | exp blood glucose/ or (blood and glucose).mp. |
| 40 | exp vascular diseases/ or (vascular and disease$).mp. |
| 41 | exp atrial fibrillation/ or (atrial and fibrillation).mp. |
| 42 | (oxidized low density lipoprotein or LDL or HDL).mp. |
| 43 | exp insulin resistance/ or (insulin and resistance).mp. |
| 44 | (hsCRP or hs-CRP).tw. or (high and sensitivity and c-reactive protein).mp. |
| 45 | (IL-6 or interleukin-6).tw. or exp interleukin-6/ |
| 46 | (IL-1b or interleukin-1b).tw. or exp interleukin-1beta/ |
| 47 | exp pulse wave analysis/ or (pulse and wave and velocity).mp. or PWV.tw. |
| 48 | ((flow-mediated and dilation) or (flow and mediated and dilation) or (vascular and mediated and dilation)).mp. or FMD.tw. |
| 49 | (Endopat or (augmentation and index) or (pulse and amplitude) or pulse-amplitude or (endotheli$ adj1 function)).mp. |
| 50 | (exp endothelium/ or endotheli$/) and (function or dysfunction).mp. |
| 51 | exp acute coronary syndrome/ or acute coronary syndrome.mp. |
| 52 | exp angina pectoris/ or exp unstable angina/ or (unstable and angina).mp. orexp stable angina/ or (stable and angina).mp. |
| 53 | exp venous thromboembolism/ or (venous and thromboembolism).mp. |
| 54 | exp venous thrombosis/ or (venous and thrombosis).mp. |
| 55 | exp insulin resistance/ or (insulin and resistance).mp. or (HOMA or HOMA-IR).mp. |
| 56 | expnf-kappa b/ or (nf-kappa b or nfkappab).mp. |
| 57 | exp intercellular adhesion molecule-1/ or ((intercellular and adhesion and molecule-1) or intercellular adhesion molecule-1 or icam 1).mp. |
| 58 | exp vascular cell adhesion molecule-1/ or ((vascular and cell and adhesion and molecule-1) or vascular cell adhesion molecule-1 or vcam 1).mp. |
| 59 | exp tumor necrosis factor-alpha/ or ((tumor and necrosis and factor-alpha) or (tnf and alpha)).mp. |
| 60 | s-leptin.mp. |
| 61 | expisoprostanes/ or isoprostane$.mp. |
| 62 | exp glycosylated hemoglobin a/ or ((hemoglobin and A1C) or (haemoglobin and A1C) or HbA1C).mp. |
| 63 | or/27-62 |
| 64 | 26 and 63 |
| 65 | randomized controlled trial.pt. |
| 66 | controlled clinical trial.pt. |
| 67 | randomized controlled trials.sh. |
| 68 | Random Allocation.sh. |
| 69 | Double-blind Method.sh. |
| 70 | Single-Blind Method.sh. |
| 71 | clinical trial.pt. |
| 72 | Clinical Trials.mp. or exp Clinical Trials/ |
| 73 | (clinic$ adj25 trial$).tw. |
| 74 | ((singl$ or doubl$ or trebl$ or tripl$) adj (mask$ or blind$)).tw. |
| 75 | Placebos/ |
| 76 | placebo$.ti,ab. |
| 77 | random$.ti,ab. |
| 78 | trial$.tw. |
| 79 | (latinadj square).tw. |
| 80 | Prospective Studies/ |
| 81 | (control$ or prospectiv$ or volunteer$).tw. |
| 82 | Cross-Over Studies/ |
| 83 | ((cohort and prospective studies) or (prospective and longitudinal)).mp. |
| 84 | or/65-83 |
| 85 | 64 and 84 |
| 86 | Animal/ not Human/ |
| 87 | 85 not 86 |
| 88 | (addresses or bibliography or biography or case reports or clinical conference or congresses or consensus development conference or consensus development conference, nih or dictionary or directory or editorial or festschrift or government publications or interview or lectures or legal cases or legislation or letter or news or newspaper article or patient education handout or periodical index or "review of reported cases").pt. |
| 89 | review$.pt. or Cross-Sectional Studies/ or Retrospective Studies/ |
| 90 | 88 or 89 |
| 91 | 87 not 90 |
| 92 | remove duplicates from 91 |

Table S3 The Most Frequently Reported Outcomes in Evaluated RCTs

| **Outcome** | **N studies**  **(%)** | **Selected Flavan-3ols intervention***  **(number of RCTs )**** | **RCT design (number of studies)** | **Blinding (number of studies)** | **Mean Sample size (Mean analyzed)** | **Direction of association(number of studies)** | **Within-arm statistical significance** | **Between-arm statistical significance** |
| --- | --- | --- | --- | --- | --- | --- | --- | --- |
| Fasting blood glucose | 114 (51.1) | Berries (17), Chocolate (19), Cinnamon (17), Green tea (21), Wine (11) | Parallel Controlled Trial (86), Cross-over trial (28) | Double-blind (70), Single-blind (15), No blinding (15), Not reported (14) | 60 (55) | Improve (51), Deteriorate (17), Unchanged (40), Unclear (3) | Yes (26), No (63), Not Reported (25) | Yes (14), No (75), NR (25) |
| Hemoglobin A1C | 45 (20.2) | Berries (5), Chocolate (6), Cinnamon (11), Green tea (10), | Parallel Controlled Trial (42), Cross-over trial (3) | Double-blind (30), Single-blind (7), No blinding (4), Not reported (4) | 70 (64) | Improve (19), Deteriorate (8), Unchanged (17), Unclear (1) | Yes (12), No (27), Not Reported (6) | Yes (3), No (33), NR (9) |
| Systolic blood pressure | 133 (59.6) | Berries (25), Chocolate (41), Green tea (20), Wine (12), | Parallel Controlled Trial (83), Cross-over trial (51) | Double-blind (79), Single-blind (25), No blinding (17), Not reported (12) | 56 (51) | Improve (85), Deteriorate (13), Unchanged (32), Unclear (2) | Yes (38), No (68), Not Reported (27) | Yes (28), No (88), NR (17) |
| Diastolic blood pressure | 129 (58.3) | Berries (24), Chocolate (39), Green tea (21), Wine (11) | Parallel Controlled Trial (81), Cross-over trial (49) | Double-blind (77), Single-blind (23), No blinding (17), Not reported (12) | 54 (49) | Improve (73), Deteriorate (13), Unchanged (41), Unclear (2) | Yes (34), No (70), Not Reported (25) | Yes (29), No (86), NR (15) |
| Total cholesterol | 141 (63.2) | Berries (23), Black tea (10), Chocolate (27), Cinnamon (12), Green tea (23), Wine (14) | Parallel Controlled Trial (98), Cross-over trial (43) | Double-blind (80), Single-blind (18), No blinding (19), Not reported (24) | 58 (53) | Improve (72), Deteriorate (28), Unchanged (36), Unclear (5) | Yes (31), No (76), Not Reported (34) | Yes (22), No (93), NR (26) |
| LDL | 131 (58.7) | Berries (18), Black tea (11), Chocolate (26), Cinnamon (11), Green tea (20), Wine (15) | Parallel Controlled Trial (93), Cross-over trial (38) | Double-blind (73), Single-blind (16), No blinding (19), Not reported (23) | 62 (57) | Improve (70), Deteriorate (21), Unchanged (35), Unclear (5) | Yes (30), No (67), Not Reported (34) | Yes (22), No (88), NR (21) |
| HDL | 142 (63.7) | Berries (21), Black tea (11), Chocolate (27), Cinnamon (11), Green tea (21), Wine (17) | Parallel Controlled Trial (98), Cross-over trial (44) | Double-blind (78), Single-blind (23), No blinding (18), Not reported (23) | 60 (55) | Improve (59), Deteriorate (29), Unchanged (52), Unclear (2) | Yes (33), No (72), Not Reported (37) | Yes (20), No (99), NR (23) |
| Triglycerides | 140 (62.8) | Berries (20), Black tea (11), Chocolate (27), Cinnamon (12), Green tea (22), Wine (16) | Parallel Controlled Trial (97), Cross-over trial (43) | Double-blind (79), Single-blind (19), No blinding (19), Not reported (23) | 61 (56) | Improve (53), Deteriorate (31), Unchanged (54), Unclear (2) | Yes (28), No (78), Not Reported (34) | Yes (18), No (97), NR (25) |
| Flow-mediated dilation | 49 (21.9) | Berries (6), Black tea (4), Chocolate (23), Grapeseed (4), Wine (4), | Parallel Controlled Trial (16), Cross-over trial (33) | Double-blind (34), Single-blind (13), No blinding (1), Not reported (1) | 42 (38) | Improve (36), Deteriorate (0), Unchanged (13), Unclear (0) | Yes (22), No (12), Not Reported (15) | Yes (25), No (14), NR (10) |
| hs-CRP | 49 (21.9) | Berries (19), Chocolate (8),  Grape seed (4), Green tea (4), Red wine (4) | Crossover trial (14),  Parallel Controlled trial (35) | Double-blind (31), Single-blind (9), No blinding (5), Not reported (4) | 52 (47) | Improve (21), Deteriorate (8), Unchanged (18), Unclear (2) | Yes (12), No (25), Not reported (12) | Yes (10), No (34), NR (5) |

* Foods or extract or powder

** ~10% RCTs of a particular intervention that reported this outcome were selected

Table S4 Description of Cohort Studies by Intervention

| **Intervention** | **N studies** | **Outcomes** | **Mean Study Duration years** | **Mean Study Duration person-years** | **Baseline Health (number of studies)** | **Mean Age** | **Mean Sample size (Mean analyzed)** | **Region where study was conducted** | **Association data available** | **Dose response data available** | **Model Adjustments** | **Additional Adjusted models** |
| --- | --- | --- | --- | --- | --- | --- | --- | --- | --- | --- | --- | --- |
| Apple | 1(plus 4 additional models in 4 cohorts) | Acute coronary syndrome (1), | 7.7 | NR | Healthy (0),  At risk for CVD (0), Existing CVD (0), Mixed health status (0), Other (1) | 55.00 | 53383 (53383) | Asia (0), Australia (0), N.America (0),  W. Europe (1) | Yes (1), No (0) | Yes (0), No (0) | Age (0), Sex (0), CVD confounders (1), Diet (1), Not Adjusted (0) | None |
| Black tea | 3 | Coronary artery disease (1), Diabetes (1), Stroke [different types of stroke outcomes] (6) | 12.6 | 793948.0 | Healthy (0),  At risk for CVD (0), ExistingCVD (1), Mixed health status (0), Other (2) | 58.50 | 47081 (42699) | Asia (1), Australia (0), N.America(0),  W. Europe (2) | Yes (8), No (0) | Yes (0), No (0) | Age (8), Sex (7), CVD confounders (7), Diet (8), Not Adjusted (0) | Green tea (1), Other (1) |
| Chocolate | 4 | Any CVD (2), Coronary artery disease (1), Diabetes (1), Heart failure (1), Stroke (4) | 9.9 | NR | Healthy (1),  At risk for CVD (0), ExistingCVD (0), Mixed health status (0), Other (3) | 61.12 | 33136 (27028) | Asia (0), Australia (0), N.America (1),  W. Europe (3) | Yes (9), No (0) | Yes (0), No (0) | Age (9), Sex (4), CVD confounders (9), Diet (9), Not Adjusted (0) | None |
| Flavanol | 9 | Any CVD (3), Atherosclerosis (1), Cardiovascular disorder (1), Coronary artery disease (1), Diabetes (4), Diastolic blood pressure (1), Myocardial infarction (2), Stroke (2), Systolic blood pressure (1) | 11.2 | NR | Healthy (1),  At risk for CVD (2), ExistingCVD (0), Mixed health status (3), Other (3) | 57.97 | 57102 (37382) | Asia (0), Australia (1), N.America(5),  W. Europe (3) | Yes (15), No (1) | Yes (0), No (0) | Age (15), Sex (4), CVD confounders (11), Diet (13), Not Adjusted (0) | Apple (1), Tea (1), Other (10) |
| Flavanol monomers | 3 | Cardiovascular disorder (2), Myocardial infarction (3), Stroke (0), Systolic blood pressure (1) | 11.9 | NR | Healthy (0),  At risk for CVD (0), ExistingCVD (1), Mixed health status (1), Other (1) | 61.37 | 44809 (24746) | Asia (0), Australia (0), N.America (1),  W. Europe (2) | Yes (6), No (0) | Yes (0), No (0) | Age (2), Sex (1), CVD confounders (1), Diet (2), Not Adjusted (0) | None |
| Grape and raisins | 3 | Coronary artery disease (3), Systolic blood pressure (3) | 24.0 | 1035257.0 | Healthy (3),  At risk for CVD (0), ExistingCVD (0), Mixed health status (0), Other (0) | 46.09 | 96633 (62461) | Asia (0), Australia (0), N.America (3),  W. Europe (0) | Yes (6), No (0) | Yes (0), No (0) | Age (6), Sex (1), CVD confounders (6), Diet (6), Not Adjusted (0) | Apple (3), Berries [not cranberry] (3), Cranberry (3), Plums (3), Other (6) |
| Green tea | 5 | Any CVD (3), Cardiovascular disorder (1), Cerebrovascular reactivity (1), Coronary artery disease (3), Myocardial infarction (1), Stroke (4) | 9.7 | NR | Healthy (1),  At risk for CVD (0), ExistingCVD (0), Mixed health status (2), Other (2) | 60.09 | 30458 (25420) | Asia (5), Australia (0), N.America(0), W. Europe (0) | Yes (13), No (0) | Yes (0), No (0) | Age (13), Sex (7), CVD confounders (11), Diet (12), Not Adjusted (0) | Other (2) |
| Red wine | 3 | Metabolism syndrome (1), Myocardial infarction (1), Stroke (1), Systolic blood pressure (1), Vascular diseases (1) | 4.5 | NR | Healthy (1),  At risk for CVD (1), ExistingCVD (0), Mixed health status (1), Other (0) | 44.53 | 7851 (5434) | Asia (0), Australia (0), N.America(0),  W. Europe (3) | Yes (5), No (0) | Yes (0), No (0) | Age (5), Sex (5), CVD confounders (5), Diet (2), Not Adjusted (0) | None |
| Tea (type not specified) | 2 | Diabetes (2), Fasting blood glucose (1) | 7.6 | NR | Healthy (1),  At risk for CVD (0), ExistingCVD (0), Mixed health status (0), Other (1) | 43.85 | 5399 (5399) | Asia (1), Australia (0), N.America(0),  W. Europe (1) | Yes (3), No (0) | Yes (0), No (0) | Age (3), Sex (3), CVD confounders (1), Diet (3), Not Adjusted (0) | None |
| Polymers | 1 | Systolic blood pressure (1) | 13.8 | NR | Healthy (0),  At risk for CVD (1), ExistingCVD (0), Mixed health status (0), Other (0) | 51.62 | 98995 (40574) | Asia (0), Australia (0), N.America(0),  W. Europe (1) | Yes (1), No (0) | Yes (0), No (0) | Age (1), Sex (0), CVD confounders (1), Diet (1), Not Adjusted (0) | None |
| Berries [not cranberry] | 3 | Coronary artery disease (3), Systolic blood pressure (3) | 24.0 | 1035257.0 | Healthy (3),  At risk for CVD (0), ExistingCVD (0), Mixed health status (0), Other (0) | 46.09 | 96633 (62461) | Asia (0), Australia (0), N.America (3),  W. Europe (0) | Yes (6), No (0) | Yes (0), No (0) | Age (6), Sex (1), CVD confounders (6), Diet (6), Not Adjusted (0) | Apple (3), Grape and raisins (3), Cranberry (3), Plums (3), Other (6) |
| Cranberry | 3 | Coronary artery disease (3), Systolic blood pressure (3) | 24.0 | 1035257.0 | Healthy (3),  At risk for CVD (0), ExistingCVD (0), Mixed health status (0), Other (0) | 46.09 | 96633 (62461) | Asia (0), Australia (0), N.America (3),  W. Europe (0) | Yes (6), No (0) | Yes (0), No (0) | Age (6), Sex (1), CVD confounders (6), Diet (6), Not Adjusted (0) | Apple (3), Berries [not cranberry] (3), Grape and raisins (3), Plums (3), Other (6) |
| Plums | 3 | Coronary artery disease (3), Systolic blood pressure (3) | 24.0 | 1035257.0 | Healthy (3),  At risk for CVD (0), ExistingCVD (0), Mixed health status (0), Other (0) | 46.09 | 96633 (62461) | Asia (0), Australia (0), N.America (3),  W. Europe (0) | Yes (6), No (0) | Yes (0), No (0) | Age (6), Sex (1), CVD confounders (6), Diet (6), Not Adjusted (0) | Apple (3), Berries [not cranberry] (3), Cranberry (3), Grape and raisins (3), Other (6) |

Table S5 Description of all studies by Intervention

| **Intervention** | **N studies** | **Mean Study Duration weeks** | **RCT design (number of studies)** | **Blinding (number of studies)** | **Baseline Health (number of studies)** | **Mean Age** | **Mean Sample size (Mean analyzed)** | **Region where study was conducted** |
| --- | --- | --- | --- | --- | --- | --- | --- | --- |
| Apple | 4 | 4.0 | Parallel trial (1), Cross-over trial (3) | Double-blind (1), Single-blind (2), No blinding (1), | Healthy (2),  At risk for CVD (2) | 44.28 | 35 (32) | Australia (1), East West Europe (2), Middle East (1) |
| Berries | 27 | 6.2 | Parallel trial (17), Cross-over trial (10) | Double-blind (13), Single-blind (5), No blinding (5), Not reported (4) | Healthy (5),  At risk for CVD (17), ExistingCVD (2), Mixed health status (2), Other (1) | 49.23 | 41 (38) | Asia (3), East Europe (1), West Europe (10), Middle East (1), North America (12) |
| Black tea | 16 | 8.6 | Parallel trial (9), Cross-over trial (7) | Double-blind (7), Single-blind (3), No blinding (1), Not reported (5) | Healthy (4),  At risk for CVD (7), ExistingCVD (1), Mixed health status (2), Other (2) | 54.45 | 64 (55) | Africa (2), Asia (3), Australia (3), West Europe (3), Middle East (2), North America (3) |
| Chocolate | 52 | 6.8 | Parallel trial (27), Cross-over trial (25) | Double-blind (34), Single-blind (13), No blinding (2), Not reported (3) | Healthy (20),  At risk for CVD (25), ExistingCVD (5), Other (2) | 49.99 | 49 (45) | Asia (1), Australia (4), West Europe (28), Middle East (3), North America (16) |
| Cinnamon | 18 | 11.0 | Parallel trial (17), Cross-over trial (1) | Double-blind (14), Single-blind (2), No blinding (0), Not reported (2) | Healthy (1),  At risk for CVD (14), Mixed health status (2), Other (0) | 54.13 | 55 (52) | Asia (3), West Europe (3), Middle East (6), North America (5), Mixed (1) |
| Cranberry | 9 | 8.7 | Parallel trial (7), Cross-over trial (2) | Double-blind (7), Single-blind (0), No blinding (1), Not reported (1) | Healthy (1),  At risk for CVD (7), ExistingCVD (1) | 51.93 | 49 (46) | Asia (1), West Europe (1), Middle East (1), North America (5), South America (1) |
| Flavan monomer | 9 | 7.1 | Parallel trial (6), Cross-over trial (3) | Double-blind (7), Single-blind (0), No blinding (1), Not reported (1) | Healthy (2),  At risk for CVD (5), ExistingCVD (1), Mixed health status (1) | 53.68 | 69 (64) | Asia (2), West Europe (5), North America (2) |
| Flavan polymer | 1 | 12.0 | Parallel trial (1), Cross-over trial (0) | Double-blind (1), Single-blind (0), No blinding (0), Not reported (0) | Healthy (0),  At risk for CVD (0), ExistingCVD (0), Mixed health status (0), Other (1) | 51.00 | 61 (53) | Asia (1) |
| Grape | 7 | 8.5 | Parallel trial (4), Cross-over trial (3) | Double-blind (5), Single-blind (0), No blinding (0), Not reported (2) | Healthy (1),  At risk for CVD (5), ExistingCVD (0), Mixed health status (0), Other (1) | 40.07 | 54 (49) | Asia (1), West Europe (1), Middle East (1), North America (4) |
| Grapeseed | 13 | 16.6 | Parallel trial (9), Cross-over trial (4) | Double-blind (12), Single-blind (0), No blinding (1), Not reported (0) | Healthy (2),  At risk for CVD (9), ExistingCVD (0), Mixed health status (1), Other (1) | 52.30 | 70 (68) | Asia (2), West Europe (6), Middle East (2), North America (3) |
| Green Tea | 31 | 12.3 | Parallel trial (26), Cross-over trial (5) | Double-blind (17), Single-blind (6), No blinding (3), Not reported (5) | Healthy (8),  At risk for CVD (21), ExistingCVD (0), Mixed health status (0), Other (2) | 51.11 | 68 (62) | Africa (0), Asia (9), Australia (1), East Europe (3), West Europe (8), Middle East (3), North America (4), South America (3) |
| Mixed/Other | 3 | 13.5 | Parallel trial (3), Cross-over trial (0) | Double-blind (0), Single-blind (3), No blinding (0), Not reported (0) | Healthy (0),  At risk for CVD (3), ExistingCVD (0), Mixed health status (0), Other (0) | 51.11 | 128 (114) | Middle East (1), North America (1), South America (1) |
| Plums | 1 | 8.0 | Parallel trial (1), Cross-over trial (0) | Double-blind (0), Single-blind (0), No blinding (1) | Healthy (1),  At risk for CVD (0), ExistingCVD (0), Mixed health status (0), Other (0) | 43.00 | 259 (248) | Asia (1) |
| Tea | 8 | 6.4 | Parallel trial (4), Cross-over trial (4) | Double-blind (4), Single-blind 2), No blinding (1), Not reported (1) | Healthy (4),  At risk for CVD (4) | 50.91 | 39 (38) | Asia (2), West Europe (4), Middle East (1), North America (1) |
| Wine | 25 | 7.5 | Parallel trial (8), Cross-over trial (17) | Double-blind (2), Single-blind (4), No blinding (9) | Healthy (15),  At risk for CVD (8), ExistingCVD (1), Mixed health status (1) | 49.04 | 45 (43) | Asia (1), Australia (3), East Europe (1), West Europe (15), Middle East (2), North America (3) |

Table S6 Flavan-3ol content by Intervention

| **Intervention** | **N studies** | **Mean total Flavan-3ols (Range)** | **Mean total monomers (Range)** | **Mean total polymers (Range)** |
| --- | --- | --- | --- | --- |
| Apple | 4 | 444.08 (146.10- 1152.00) | 356.02 (26.97- 1152.00) | 271.27 (271.27- 271.27) |
| Berries | 27 | 233.1 (0.40- 661.22) | 9.45 (0- 29.00) | 253.28 (0.40- 637.81) |
| Black tea | 16 | 256.20 (19.30 – 550.98) | 227.43 (19.30 – 484.38) | 30.01 (0.00- 66.60) |
| Chocolate | 52 | 618.68 (2.70- 8301.81) | 99.30 (5.00- 602.70) | 425.84 (21.20- 1700.88) |
| Cinnamon | 18 | 156.39 (9.70- 970.11) | 0.00 (0.00- 0.00) | 151.74 (9.70- 970.11) |
| Cranberry | 9 | 300.50 (51.74- 1224.52) | 5.66 (2.83- 8.26) | 125.46 (48.91- 236.00) |
| Flavan monomer | 9 | 350.67 (100.00- 800.00) | 460.00 (300.00- 800.00) | 0.00 (0.00- 0.00) |
| Flavan polymer | 1 | 200.00 (200.00- 200.00) | 0.00 | 200.00 (200.00- 200.00) |
| Grape | 7 | 267.57 (2.92- 1230.00) | 27.62 (0.00- 170.00) | 279.94 (22.28- 1060.00) |
| Grapeseed | 13 | 76.09 (1.92- 200.00) | 15.06 (0.12- 30.00) | 93.93 (1.80- 170.00) |
| Green Tea | 31 | 473.39 (7.52- 1345.05) | 472.06 (27.00- 1345.05) | 34.80 (0.00-273.00) |
| Mixed/Other | 3 | 493.33 (425.80- 594.50) | 371.67 (190.93- 552.42) | 155.43 (42.08- 268.78) |
| Plums | 1 | Questionable (dried) |  |  |
| Tea | 8 | 250.42 (3.62- 579.36) | 233.65 (3.36- 509.33) | 23.12 (0.00- 70.03) |
| Wine | 25 | 123.73 (23.33- 254.31) | 31.89 (6.32- 69.25) | 84.36 (16.88- 185.06) |

Table S7 Description of outcomes reported in Apple Intervention studies

| **Outcomes in Apple intervention RCT** | **N studies** | **Association with outcome** | **With-in arm significance** | **Between arm significance** |
| --- | --- | --- | --- | --- |
| Diastolic blood pressure | 3 | Improve (2), Deteriorate (0), Unchanged (1), Unclear (0) | Yes (0), No (2), NR (1) | Yes (0), No (3), NR (0) |
| Fasting blood glucose | 1 | Improve (0), Deteriorate (0), Unchanged (1), Unclear (0) | Yes (0), No (1), NR (0) | Yes (0), No (1), NR (0) |
| Flow-mediated dilation (FMD) | 2 | Improve (1), Deteriorate (0), Unchanged (1), Unclear (0) | Yes (0), No (1), NR (1) | Yes (1), No (1), NR (0) |
| HDL | 3 | Improve (0), Deteriorate (1), Unchanged (2), Unclear (0) | Yes (0), No (2), NR (1) | Yes (0), No (3), NR (0) |
| LDL | 3 | Improve (1), Deteriorate (1), Unchanged (0), Unclear (1) | Yes (1), No (1), NR (1) | Yes (0), No (3), NR (0) |
| Systolic blood pressure | 3 | Improve (2), Deteriorate (0), Unchanged (1), Unclear (0) | Yes (0), No (2), NR (1) | Yes (1), No (2), NR (0) |
| Total Cholesterol | 3 | Improve (1), Deteriorate (1), Unchanged (0), Unclear (1) | Yes (1), No (1), NR (1) | Yes (0), No (3), NR (0) |
| Triglycerides | 3 | Improve (0), Deteriorate (1), Unchanged (2), Unclear (0) | Yes (0), No (2), NR (1) | Yes (1), No (2), NR (0) |
| hsCRP or hs-CRP | 1 | Improve (0), Deteriorate (0), Unchanged (1), Unclear (0) | Yes (0), No (1), NR (0) | Yes (0), No (1), NR (0) |

Table S8 Description of outcomes reported in Berries Intervention studies

| **Outcome** | **N studies** | **Association with outcome** | **With-in arm significance** | **Between arm significance** |
| --- | --- | --- | --- | --- |
| Diastolic blood pressure | 17 | Improve (11), Deteriorate (1), Unchanged (5), Unclear (0) | Yes (2), No (11), NR (4) | Yes (5), No (12), NR (0) |
| Endothelial dysfunction | 2 | Improve (1), Deteriorate (0), Unchanged (0), Unclear (1) | Yes (1), No (0), NR (1) | Yes (1), No (1), NR (0) |
| Fasting blood glucose | 12 | Improve (5), Deteriorate (2), Unchanged (5), Unclear (0) | Yes (0), No (8), NR (4) | Yes (0), No (11), NR (1) |
| Flow-mediated dilation (FMD) | 5 | Improve (4), Deteriorate (0), Unchanged (1), Unclear (0) | Yes (2), No (1), NR (2) | Yes (4), No (1), NR (0) |
| HDL | 15 | Improve (3), Deteriorate (5), Unchanged (7), Unclear (0) | Yes (0), No (9), NR (6) | Yes (1), No (13), NR (1) |
| HbA1C | 4 | Improve (1), Deteriorate (2), Unchanged (1), Unclear (0) | Yes (2), No (1), NR (1) | Yes (1), No (2), NR (1) |
| ICAM-1 | 10 | Improve (6), Deteriorate (2), Unchanged (2), Unclear (0) | Yes (0), No (7), NR (3) | Yes (1), No (8), NR (1) |
| Insulin resistance or HOMA-IR | 2 | Improve (2), Deteriorate (0), Unchanged (0), Unclear (0) | Yes (1), No (1), NR (0) | Yes (1), No (1), NR (0) |
| Interleukin-1beta (IL-1B) | 3 | Improve (1), Deteriorate (0), Unchanged (2), Unclear (0) | Yes (0), No (2), NR (1) | Yes (0), No (3), NR (0) |
| Interleukin-6 (IL-6) | 11 | Improve (7), Deteriorate (0), Unchanged (4), Unclear (0) | Yes (3), No (3), NR (5) | Yes (5), No (5), NR (1) |
| Isoprostane | 4 | Improve (3), Deteriorate (0), Unchanged (1), Unclear (0) | Yes (1), No (1), NR (2) | Yes (2), No (2), NR (0) |
| LDL | 14 | Improve (7), Deteriorate (3), Unchanged (4), Unclear (0) | Yes (2), No (7), NR (5) | Yes (3), No (10), NR (1) |
| LDL/HDL or LDL:HDL ratio | 2 | Improve (1), Deteriorate (0), Unchanged (1), Unclear (0) | Yes (0), No (2), NR (0) | Yes (0), No (2), NR (0) |
| Oxidized low density lipoprotein | 3 | Improve (2), Deteriorate (1), Unchanged (0), Unclear (0) | Yes (0), No (0), NR (3) | Yes (3), No (0), NR (0) |
| Pulse wave analysis or pulse wave velocity | 7 | Improve (3), Deteriorate (0), Unchanged (3), Unclear (1) | Yes (1), No (5), NR (1) | Yes (1), No (5), NR (1) |
| Systolic blood pressure | 18 | Improve (14), Deteriorate (1), Unchanged (3), Unclear (0) | Yes (3), No (10), NR (5) | Yes (4), No (13), NR (1) |
| Total Cholesterol | 17 | Improve (6), Deteriorate (3), Unchanged (8), Unclear (0) | Yes (2), No (10), NR (5) | Yes (5), No (11), NR (1) |
| Triglycerides | 15 | Improve (3), Deteriorate (5), Unchanged (7), Unclear (0) | Yes (0), No (10), NR (5) | Yes (1), No (13), NR (1) |
| Tumor necrosis factor-alpha (TNF-alpha) | 9 | Improve (7), Deteriorate (1), Unchanged (1), Unclear (0) | Yes (4), No (3), NR (2) | Yes (4), No (4), NR (1) |
| VCAM-1 | 10 | Improve (6), Deteriorate (4), Unchanged (0), Unclear (0) | Yes (2), No (4), NR (4) | Yes (2), No (7), NR (1) |
| hsCRP or hs-CRP | 12 | Improve (7), Deteriorate (3), Unchanged (2), Unclear (0) | Yes (2), No (6), NR (4) | Yes (4), No (8), NR (0) |

Table S9 Description of outcomes reported in Black tea Intervention studies

| **Outcome** | **N studies** | **Association with outcome** | **With-in arm significance** | **Between arm significance** |
| --- | --- | --- | --- | --- |
| Diastolic blood pressure | 5 | Improve (0), Deteriorate (0), Unchanged (5), Unclear (0) | Yes (0), No (4), NR (1) | Yes (1), No (4), NR (0) |
| E-selectin | 1 | Improve (0), Deteriorate (0), Unchanged (1), Unclear (0) | Yes (0), No (1), NR (0) | Yes (0), No (1), NR (0) |
| Fasting blood glucose | 6 | Improve (3), Deteriorate (1), Unchanged (2), Unclear (0) | Yes (2), No (2), NR (2) | Yes (0), No (5), NR (1) |
| Flow-mediated dilation (FMD) | 4 | Improve (4), Deteriorate (0), Unchanged (0), Unclear (0) | Yes (2), No (0), NR (2) | Yes (1), No (2), NR (1) |
| HDL | 11 | Improve (5), Deteriorate (2), Unchanged (4), Unclear (0) | Yes (2), No (4), NR (5) | Yes (0), No (10), NR (1) |
| HbA1C | 1 | Improve (1), Deteriorate (0), Unchanged (0), Unclear (0) | Yes (0), No (1), NR (0) | Yes (0), No (0), NR (1) |
| ICAM-1 | 1 | Improve (1), Deteriorate (0), Unchanged (0), Unclear (0) | Yes (0), No (0), NR (1) | Yes (0), No (1), NR (0) |
| Insulin resistance or HOMA-IR | 2 | Improve (2), Deteriorate (0), Unchanged (0), Unclear (0) | Yes (0), No (0), NR (2) | Yes (0), No (2), NR (0) |
| Interleukin-6 (IL-6) | 1 | Improve (1), Deteriorate (0), Unchanged (0), Unclear (0) | Yes (0), No (0), NR (1) | Yes (0), No (1), NR (0) |
| LDL | 11 | Improve (6), Deteriorate (2), Unchanged (3), Unclear (0) | Yes (1), No (5), NR (5) | Yes (2), No (8), NR (1) |
| LDL/HDL or LDL:HDL ratio | 3 | Improve (1), Deteriorate (0), Unchanged (2), Unclear (0) | Yes (1), No (0), NR (2) | Yes (0), No (2), NR (1) |
| Pulse wave analysis or pulse wave velocity | 4 | Improve (4), Deteriorate (0), Unchanged (0), Unclear (0) | Yes (3), No (1), NR (0) | Yes (2), No (1), NR (1) |
| Systolic blood pressure | 6 | Improve (2), Deteriorate (0), Unchanged (4), Unclear (0) | Yes (0), No (5), NR (1) | Yes (1), No (4), NR (1) |
| Total Cholesterol | 10 | Improve (3), Deteriorate (4), Unchanged (3), Unclear (0) | Yes (2), No (4), NR (4) | Yes (1), No (8), NR (1) |
| Triglycerides | 11 | Improve (5), Deteriorate (1), Unchanged (5), Unclear (0) | Yes (2), No (4), NR (5) | Yes (1), No (9), NR (1) |
| Tumor necrosis factor-alpha (TNF-alpha) | 1 | Improve (0), Deteriorate (1), Unchanged (0), Unclear (0) | Yes (0), No (0), NR (1) | Yes (0), No (1), NR (0) |
| VCAM-1 | 2 | Improve (0), Deteriorate (1), Unchanged (1), Unclear (0) | Yes (0), No (1), NR (1) | Yes (0), No (2), NR (0) |
| hsCRP or hs-CRP | 1 | Improve (0), Deteriorate (1), Unchanged (0), Unclear (0) | Yes (0), No (0), NR (1) | Yes (0), No (1), NR (0) |

Table S10 Description of outcomes reported in Chocolate Intervention studies

| **Outcome** | **N studies** | **Association with outcome** | **With-in arm significance** | **Between arm significance** |
| --- | --- | --- | --- | --- |
| Cerebral blood flow (CBF) | 2 | Improve (1), Deteriorate (0), Unchanged (1), Unclear (0) | Yes (0), No (2), NR (0) | Yes (0), No (2), NR (0) |
| Cerebrovascular reactivity (CVR) | 2 | Improve (1), Deteriorate (0), Unchanged (1), Unclear (0) | Yes (0), No (2), NR (0) | Yes (0), No (2), NR (0) |
| Diastolic blood pressure | 39 | Improve (24), Deteriorate (3), Unchanged (12), Unclear (0) | Yes (13), No (19), NR (7) | Yes (9), No (23), NR (7) |
| E-selectin | 4 | Improve (2), Deteriorate (0), Unchanged (2), Unclear (0) | Yes (1), No (2), NR (1) | Yes (0), No (3), NR (1) |
| Endothelial dysfunction | 10 | Improve (5), Deteriorate (0), Unchanged (5), Unclear (0) | Yes (4), No (6), NR (0) | Yes (4), No (6), NR (0) |
| Fasting blood glucose | 19 | Improve (7), Deteriorate (4), Unchanged (7), Unclear (1) | Yes (5), No (11), NR (3) | Yes (1), No (14), NR (4) |
| Flow-mediated dilation (FMD) | 23 | Improve (20), Deteriorate (0), Unchanged (3), Unclear (0) | Yes (14), No (4), NR (5) | Yes (16), No (4), NR (3) |
| HDL | 27 | Improve (13), Deteriorate (3), Unchanged (10), Unclear (1) | Yes (9), No (11), NR (7) | Yes (7), No (15), NR (5) |
| HbA1C | 6 | Improve (2), Deteriorate (1), Unchanged (3), Unclear (0) | Yes (2), No (4), NR (0) | Yes (0), No (5), NR (1) |
| ICAM-1 | 4 | Improve (2), Deteriorate (1), Unchanged (1), Unclear (0) | Yes (1), No (3), NR (0) | Yes (0), No (4), NR (0) |
| Insulin resistance or HOMA-IR | 12 | Improve (8), Deteriorate (0), Unchanged (4), Unclear (0) | Yes (6), No (5), NR (1) | Yes (6), No (4), NR (2) |
| Interleukin-1beta (IL-1B) | 3 | Improve (1), Deteriorate (0), Unchanged (2), Unclear (0) | Yes (0), No (3), NR (0) | Yes (0), No (3), NR (0) |
| Interleukin-6 (IL-6) | 5 | Improve (1), Deteriorate (1), Unchanged (3), Unclear (0) | Yes (0), No (4), NR (1) | Yes (0), No (5), NR (0) |
| Isoprostane | 4 | Improve (3), Deteriorate (0), Unchanged (1), Unclear (0) | Yes (2), No (2), NR (0) | Yes (2), No (2), NR (0) |
| LDL | 26 | Improve (12), Deteriorate (3), Unchanged (10), Unclear (1) | Yes (7), No (13), NR (6) | Yes (4), No (19), NR (3) |
| LDL/HDL or LDL:HDL ratio | 3 | Improve (0), Deteriorate (0), Unchanged (2), Unclear (1) | Yes (1), No (0), NR (2) | Yes (1), No (1), NR (1) |
| Oxidized low density lipoprotein | 6 | Improve (5), Deteriorate (0), Unchanged (1), Unclear (0) | Yes (2), No (1), NR (3) | Yes (2), No (3), NR (1) |
| Postprandial blood glucose | 6 | Improve (3), Deteriorate (0), Unchanged (3), Unclear (0) | Yes (2), No (3), NR (1) | Yes (1), No (4), NR (1) |
| Pulse wave analysis or pulse wave velocity | 13 | Improve (11), Deteriorate (1), Unchanged (1), Unclear (0) | Yes (9), No (2), NR (2) | Yes (10), No (1), NR (2) |
| Quantitative Insulin Sensitivity Check Index (QUICKI) | 7 | Improve (4), Deteriorate (1), Unchanged (2), Unclear (0) | Yes (3), No (3), NR (1) | Yes (2), No (3), NR (2) |
| Systolic blood pressure | 41 | Improve (28), Deteriorate (4), Unchanged (9), Unclear (0) | Yes (16), No (18), NR (7) | Yes (9), No (25), NR (9) |
| Total Cholesterol | 27 | Improve (13), Deteriorate (5), Unchanged (7), Unclear (2) | Yes (6), No (14), NR (7) | Yes (3), No (17), NR (7) |
| Triglycerides | 27 | Improve (9), Deteriorate (2), Unchanged (15), Unclear (1) | Yes (5), No (16), NR (6) | Yes (4), No (19), NR (4) |
| Tumor necrosis factor-alpha (TNF-alpha) | 4 | Improve (2), Deteriorate (1), Unchanged (1), Unclear (0) | Yes (1), No (2), NR (1) | Yes (1), No (3), NR (0) |
| VCAM-1 | 6 | Improve (2), Deteriorate (2), Unchanged (2), Unclear (0) | Yes (0), No (5), NR (1) | Yes (0), No (6), NR (0) |
| hsCRP or hs-CRP | 8 | Improve (4), Deteriorate (0), Unchanged (3), Unclear (1) | Yes (2), No (4), NR (2) | Yes (1), No (6), NR (1) |

Table S11 Description of outcomes reported in Cinnamon Intervention studies

| **Outcome** | **N studies** | **Association with outcome** | **With-in arm significance** | **Between arm significance** |
| --- | --- | --- | --- | --- |
| Diastolic blood pressure | 4 | Improve (1), Deteriorate (0), Unchanged (3), Unclear (0) | Yes (1), No (3), NR (0) | Yes (1), No (3), NR (0) |
| Fasting blood glucose | 17 | Improve (13), Deteriorate (1), Unchanged (3), Unclear (0) | Yes (10), No (4), NR (3) | Yes (5), No (8), NR (4) |
| HDL | 11 | Improve (2), Deteriorate (1), Unchanged (8), Unclear (0) | Yes (0), No (9), NR (2) | Yes (0), No (9), NR (2) |
| HbA1C | 11 | Improve (6), Deteriorate (1), Unchanged (4), Unclear (0) | Yes (5), No (5), NR (1) | Yes (2), No (8), NR (1) |
| Insulin resistance or HOMA-IR | 5 | Improve (5), Deteriorate (0), Unchanged (0), Unclear (0) | Yes (4), No (1), NR (0) | Yes (4), No (1), NR (0) |
| LDL | 11 | Improve (6), Deteriorate (3), Unchanged (2), Unclear (0) | Yes (2), No (7), NR (2) | Yes (1), No (7), NR (3) |
| Postprandial blood glucose | 3 | Improve (3), Deteriorate (0), Unchanged (0), Unclear (0) | Yes (3), No (0), NR (0) | Yes (2), No (0), NR (1) |
| Quantitative Insulin Sensitivity Check Index (QUICKI) | 2 | Improve (1), Deteriorate (0), Unchanged (1), Unclear (0) | Yes (2), No (0), NR (0) | Yes (2), No (0), NR (0) |
| Systolic blood pressure | 4 | Improve (3), Deteriorate (0), Unchanged (1), Unclear (0) | Yes (1), No (2), NR (1) | Yes (2), No (2), NR (0) |
| Total Cholesterol | 12 | Improve (7), Deteriorate (3), Unchanged (2), Unclear (0) | Yes (2), No (7), NR (3) | Yes (1), No (9), NR (2) |
| Triglycerides | 12 | Improve (6), Deteriorate (2), Unchanged (4), Unclear (0) | Yes (4), No (5), NR (1) | Yes (1), No (9), NR (2) |
| hsCRP or hs-CRP | 2 | Improve (2), Deteriorate (0), Unchanged (0), Unclear (0) | Yes (2), No (0), NR (0) | Yes (2), No (0), NR (0) |

Table S12 Description of outcomes reported in Cranberry Intervention studies

| **Outcome** | **N studies** | **Association with outcome** | **With-in arm significance** | **Between arm significance** |
| --- | --- | --- | --- | --- |
| Diastolic blood pressure | 7 | Improve (5), Deteriorate (1), Unchanged (1), Unclear (0) | Yes (0), No (3), NR (4) | Yes (1), No (6), NR (0) |
| Endothelial dysfunction | 1 | Improve (1), Deteriorate (0), Unchanged (0), Unclear (0) | Yes (0), No (1), NR (0) | Yes (0), No (1), NR (0) |
| Fasting blood glucose | 5 | Improve (3), Deteriorate (1), Unchanged (1), Unclear (0) | Yes (1), No (2), NR (2) | Yes (2), No (3), NR (0) |
| Flow-mediated dilation (FMD) | 1 | Improve (0), Deteriorate (0), Unchanged (1), Unclear (0) | Yes (0), No (0), NR (1) | Yes (0), No (1), NR (0) |
| HDL | 6 | Improve (2), Deteriorate (1), Unchanged (3), Unclear (0) | Yes (0), No (3), NR (3) | Yes (1), No (5), NR (0) |
| HbA1C | 1 | Improve (1), Deteriorate (0), Unchanged (0), Unclear (0) | Yes (0), No (1), NR (0) | Yes (0), No (1), NR (0) |
| ICAM-1 | 4 | Improve (2), Deteriorate (1), Unchanged (1), Unclear (0) | Yes (0), No (2), NR (2) | Yes (0), No (4), NR (0) |
| Insulin resistance or HOMA-IR | 3 | Improve (0), Deteriorate (0), Unchanged (3), Unclear (0) | Yes (0), No (1), NR (2) | Yes (1), No (2), NR (0) |
| Interleukin-1beta (IL-1B) | 1 | Improve (0), Deteriorate (0), Unchanged (1), Unclear (0) | Yes (0), No (1), NR (0) | Yes (0), No (1), NR (0) |
| Interleukin-6 (IL-6) | 3 | Improve (0), Deteriorate (1), Unchanged (2), Unclear (0) | Yes (0), No (3), NR (0) | Yes (0), No (3), NR (0) |
| LDL | 4 | Improve (2), Deteriorate (1), Unchanged (1), Unclear (0) | Yes (1), No (1), NR (2) | Yes (1), No (3), NR (0) |
| Oxidized low density lipoprotein | 4 | Improve (2), Deteriorate (0), Unchanged (2), Unclear (0) | Yes (0), No (3), NR (1) | Yes (1), No (3), NR (0) |
| Pulse wave analysis or pulse wave velocity | 3 | Improve (1), Deteriorate (0), Unchanged (1), Unclear (1) | Yes (0), No (1), NR (2) | Yes (1), No (2), NR (0) |
| Systolic blood pressure | 7 | Improve (5), Deteriorate (1), Unchanged (1), Unclear (0) | Yes (0), No (4), NR (3) | Yes (0), No (7), NR (0) |
| Total Cholesterol | 6 | Improve (4), Deteriorate (0), Unchanged (2), Unclear (0) | Yes (1), No (2), NR (3) | Yes (1), No (5), NR (0) |
| Triglycerides | 5 | Improve (4), Deteriorate (0), Unchanged (1), Unclear (0) | Yes (0), No (3), NR (2) | Yes (1), No (4), NR (0) |
| Tumor necrosis factor-alpha (TNF-alpha) | 2 | Improve (0), Deteriorate (0), Unchanged (2), Unclear (0) | Yes (0), No (2), NR (0) | Yes (0), No (2), NR (0) |
| VCAM-1 | 3 | Improve (1), Deteriorate (2), Unchanged (0), Unclear (0) | Yes (1), No (1), NR (1) | Yes (0), No (3), NR (0) |
| hsCRP or hs-CRP | 7 | Improve (2), Deteriorate (0), Unchanged (4), Unclear (1) | Yes (0), No (0), NR (0) | Yes (1), No (6), NR (0) |

Table S13 Description of outcomes reported in Flavan monomer Intervention studies

| **Outcome** | **N studies** | **Association with outcome** | **With-in arm significance** | **Between arm significance** |
| --- | --- | --- | --- | --- |
| Diastolic blood pressure | 4 | Improve (2), Deteriorate (0), Unchanged (2), Unclear (0) | Yes (0), No (3), NR (1) | Yes (1), No (2), NR (1) |
| Fasting blood glucose | 6 | Improve (3), Deteriorate (1), Unchanged (2), Unclear (0) | Yes (0), No (5), NR (1) | Yes (1), No (4), NR (1) |
| Flow-mediated dilation (FMD) | 3 | Improve (2), Deteriorate (0), Unchanged (1), Unclear (0) | Yes (0), No (3), NR (0) | Yes (0), No (1), NR (2) |
| HDL | 7 | Improve (3), Deteriorate (3), Unchanged (1), Unclear (0) | Yes (2), No (4), NR (1) | Yes (0), No (6), NR (1) |
| HbA1C | 2 | Improve (1), Deteriorate (1), Unchanged (0), Unclear (0) | Yes (0), No (1), NR (1) | Yes (0), No (2), NR (0) |
| ICAM-1 | 1 | Improve (0), Deteriorate (0), Unchanged (1), Unclear (0) | Yes (0), No (1), NR (0) | Yes (0), No (1), NR (0) |
| Insulin resistance or HOMA-IR | 3 | Improve (2), Deteriorate (1), Unchanged (0), Unclear (0) | Yes (1), No (1), NR (1) | Yes (0), No (3), NR (0) |
| Interleukin-1beta (IL-1B) | 1 | Improve (0), Deteriorate (0), Unchanged (1), Unclear (0) | Yes (0), No (1), NR (0) | Yes (0), No (1), NR (0) |
| Interleukin-6 (IL-6) | 1 | Improve (0), Deteriorate (0), Unchanged (1), Unclear (0) | Yes (0), No (1), NR (0) | Yes (0), No (1), NR (0) |
| LDL | 7 | Improve (4), Deteriorate (1), Unchanged (2), Unclear (0) | Yes (3), No (3), NR (1) | Yes (1), No (5), NR (1) |
| Oxidized low density lipoprotein | 1 | Improve (1), Deteriorate (0), Unchanged (0), Unclear (0) | Yes (1), No (0), NR (0) | Yes (0), No (0), NR (1) |
| Postprandial blood glucose | 1 | Improve (1), Deteriorate (0), Unchanged (0), Unclear (0) | Yes (0), No (0), NR (1) | Yes (0), No (1), NR (0) |
| Pulse wave analysis or pulse wave velocity | 1 | Improve (0), Deteriorate (0), Unchanged (1), Unclear (0) | Yes (0), No (1), NR (0) | Yes (0), No (1), NR (0) |
| Systolic blood pressure | 4 | Improve (2), Deteriorate (0), Unchanged (2), Unclear (0) | Yes (0), No (3), NR (1) | Yes (0), No (3), NR (1) |
| Total Cholesterol | 7 | Improve (4), Deteriorate (1), Unchanged (2), Unclear (0) | Yes (3), No (3), NR (1) | Yes (0), No (5), NR (2) |
| Triglycerides | 7 | Improve (2), Deteriorate (3), Unchanged (2), Unclear (0) | Yes (2), No (4), NR (1) | Yes (0), No (5), NR (2) |
| Tumor necrosis factor-alpha (TNF-alpha) | 1 | Improve (0), Deteriorate (0), Unchanged (1), Unclear (0) | Yes (0), No (1), NR (0) | Yes (0), No (1), NR (0) |
| VCAM-1 | 1 | Improve (0), Deteriorate (0), Unchanged (1), Unclear (0) | Yes (0), No (1), NR (0) | Yes (0), No (1), NR (0) |
| hsCRP or hs-CRP | 2 | Improve (0), Deteriorate (0), Unchanged (2), Unclear (0) | Yes (0), No (2), NR (0) | Yes (0), No (2), NR (0) |

Table S14 Description of outcomes reported in Flavan polymer Intervention studies

| **Outcome** | **N studies** | **Association with outcome** | **With-in arm significance** | **Between arm significance** |
| --- | --- | --- | --- | --- |
| Diastolic blood pressure | 1 | Improve (0), Deteriorate (1), Unchanged (0), Unclear (0) | Yes (0), No (0), NR (1) | Yes (0), No (1), NR (0) |
| Fasting blood glucose | 1 | Improve (0), Deteriorate (1), Unchanged (0), Unclear (0) | Yes (0), No (0), NR (1) | Yes (0), No (0), NR (1) |
| HDL | 1 | Improve (1), Deteriorate (0), Unchanged (0), Unclear (0) | Yes (0), No (0), NR (0) | Yes (0), No (1), NR (0) |
| HbA1C | 1 | Improve (0), Deteriorate (0), Unchanged (1), Unclear (0) | Yes (0), No (1), NR (0) | Yes (0), No (1), NR (0) |
| LDL | 1 | Improve (0), Deteriorate (1), Unchanged (0), Unclear (0) | Yes (0), No (1), NR (0) | Yes (0), No (1), NR (0) |
| Oxidized low density lipoprotein | 1 | Improve (1), Deteriorate (0), Unchanged (0), Unclear (0) | Yes (1), No (0), NR (0) | Yes (1), No (0), NR (0) |
| Systolic blood pressure | 1 | Improve (0), Deteriorate (1), Unchanged (0), Unclear (0) | Yes (0), No (0), NR (1) | Yes (0), No (1), NR (0) |
| Total Cholesterol | 1 | Improve (0), Deteriorate (1), Unchanged (0), Unclear (0) | Yes (0), No (1), NR (0) | Yes (0), No (1), NR (0) |
| Triglycerides | 1 | Improve (1), Deteriorate (0), Unchanged (0), Unclear (0) | Yes (0), No (1), NR (0) | Yes (0), No (1), NR (0) |

Table S15 Description of outcomes reported in Grape Intervention studies

| **Outcome** | **N studies** | **Association with outcome** | **With-in arm significance** | **Between arm significance** |
| --- | --- | --- | --- | --- |
| ICAM-1 | 2 | Improve (1), Deteriorate (1), Unchanged (0), Unclear (0) | Yes (0), No (1), NR (1) | Yes (1), No (1), NR (0) |
| Diastolic blood pressure | 4 | Improve (3), Deteriorate (0), Unchanged (1), Unclear (0) | Yes (2), No (1), NR (1) | Yes (0), No (2), NR (2) |
| Fasting blood glucose | 6 | Improve (2), Deteriorate (3), Unchanged (1), Unclear (0) | Yes (1), No (3), NR (2) | Yes (1), No (4), NR (1) |
| Flow-mediated dilation (FMD) | 1 | Improve (1), Deteriorate (0), Unchanged (0), Unclear (0) | Yes (0), No (0), NR (1) | Yes (1), No (0), NR (0) |
| HDL | 6 | Improve (2), Deteriorate (2), Unchanged (2), Unclear (0) | Yes (0), No (4), NR (2) | Yes (0), No (4), NR (2) |
| Insulin resistance or HOMA-IR | 2 | Improve (0), Deteriorate (1), Unchanged (1), Unclear (0) | Yes (0), No (2), NR (0) | Yes (0), No (2), NR (0) |
| Interleukin-1beta (IL-1B) | 1 | Improve (0), Deteriorate (0), Unchanged (1), Unclear (0) | Yes (0), No (1), NR (0) | Yes (0), No (1), NR (0) |
| Interleukin-6 (IL-6) | 1 | Improve (0), Deteriorate (1), Unchanged (0), Unclear (0) | Yes (0), No (1), NR (0) | Yes (0), No (1), NR (0) |
| LDL | 5 | Improve (3), Deteriorate (1), Unchanged (1), Unclear (0) | Yes (1), No (3), NR (1) | Yes (1), No (2), NR (2) |
| Oxidized low density lipoprotein | 1 | Improve (0), Deteriorate (0), Unchanged (1), Unclear (0) | Yes (0), No (1), NR (0) | Yes (0), No (1), NR (0) |
| Systolic blood pressure | 5 | Improve (4), Deteriorate (0), Unchanged (1), Unclear (0) | Yes (2), No (1), NR (2) | Yes (1), No (2), NR (2) |
| Total Cholesterol | 5 | Improve (3), Deteriorate (2), Unchanged (0), Unclear (0) | Yes (2), No (2), NR (1) | Yes (1), No (2), NR (2) |
| Triglycerides | 6 | Improve (2), Deteriorate (3), Unchanged (1), Unclear (0) | Yes (1), No (4), NR (1) | Yes (0), No (4), NR (2) |
| Tumor necrosis factor-alpha (TNF-alpha) | 1 | Improve (1), Deteriorate (0), Unchanged (0), Unclear (0) | Yes (0), No (1), NR (0) | Yes (0), No (1), NR (0) |
| VCAM-1 | 2 | Improve (0), Deteriorate (1), Unchanged (1), Unclear (0) | Yes (0), No (1), NR (1) | Yes (0), No (2), NR (0) |
| hsCRP or hs-CRP | 1 | Improve (0), Deteriorate (1), Unchanged (0), Unclear (0) | Yes (0), No (1), NR (0) | Yes (0), No (1), NR (0) |

Table S16 Description of outcomes reported in Grapeseed Intervention studies

| **Outcome** | **N studies** | **Association with outcome** | **With-in arm significance** | **Between arm significance** |
| --- | --- | --- | --- | --- |
| Diastolic blood pressure | 9 | Improve (4), Deteriorate (1), Unchanged (3), Unclear (0) | Yes (4), No (5), NR (0) | Yes (4), No (5), NR (0) |
| Endothelial dysfunction | 1 | Improve (0), Deteriorate (0), Unchanged (1), Unclear (0) | Yes (0), No (1), NR (0) | Yes (0), No (1), NR (0) |
| Fasting blood glucose | 4 | Improve (1), Deteriorate (0), Unchanged (3), Unclear (0) | Yes (0), No (3), NR (1) | Yes (0), No (3), NR (1) |
| Flow-mediated dilation (FMD) | 4 | Improve (1), Deteriorate (0), Unchanged (3), Unclear (0) | Yes (1), No (3), NR (0) | Yes (1), No (3), NR (0) |
| HDL | 9 | Improve (3), Deteriorate (2), Unchanged (4), Unclear (0) | Yes (1), No (8), NR (0) | Yes (1), No (8), NR (0) |
| HbA1C | 2 | Improve (0), Deteriorate (0), Unchanged (2), Unclear (0) | Yes (0), No (2), NR (0) | Yes (0), No (2), NR (0) |
| ICAM-1 | 1 | Improve (0), Deteriorate (0), Unchanged (1), Unclear (0) | Yes (0), No (1), NR (0) | Yes (0), No (1), NR (0) |
| Insulin resistance or HOMA-IR | 2 | Improve (1), Deteriorate (0), Unchanged (1), Unclear (0) | Yes (0), No (2), NR (0) | Yes (0), No (2), NR (0) |
| Interleukin-6 (IL-6) | 1 | Improve (0), Deteriorate (0), Unchanged (1), Unclear (0) | Yes (0), No (1), NR (0) | Yes (0), No (1), NR (0) |
| Isoprostane | 1 | Improve (0), Deteriorate (0), Unchanged (1), Unclear (0) | Yes (0), No (1), NR (0) | Yes (0), No (1), NR (0) |
| LDL | 8 | Improve (5), Deteriorate (1), Unchanged (2), Unclear (0) | Yes (3), No (5), NR (0) | Yes (3), No (5), NR (0) |
| LDL/HDL or LDL:HDL ratio | 1 | Improve (1), Deteriorate (0), Unchanged (0), Unclear (0) | Yes (0), No (0), NR (1) | Yes (0), No (0), NR (1) |
| Myocardial infarction | 2 | Improve (0), Deteriorate (0), Unchanged (2), Unclear (0) | Yes (0), No (2), NR (0) | Yes (0), No (2), NR (0) |
| Oxidized low density lipoprotein | 5 | Improve (4), Deteriorate (0), Unchanged (1), Unclear (0) | Yes (1), No (3), NR (1) | Yes (1), No (3), NR (1) |
| Postprandial blood glucose | 2 | Improve (2), Deteriorate (0), Unchanged (0), Unclear (0) | Yes (0), No (1), NR (1) | Yes (0), No (1), NR (1) |
| Stroke | 2 | Improve (0), Deteriorate (0), Unchanged (2), Unclear (0) | Yes (0), No (1), NR (1) | Yes (0), No (1), NR (1) |
| Systolic blood pressure | 9 | Improve (3), Deteriorate (1), Unchanged (4), Unclear (0) | Yes (3), No (6), NR (0) | Yes (3), No (6), NR (0) |
| Total Cholesterol | 9 | Improve (6), Deteriorate (0), Unchanged (3), Unclear (0) | Yes (2), No (7), NR (0) | Yes (2), No (7), NR (0) |
| Triglycerides | 8 | Improve (3), Deteriorate (1), Unchanged (4), Unclear (0) | Yes (0), No (8), NR (0) | Yes (0), No (8), NR (0) |
| Tumor necrosis factor-alpha (TNF-alpha) | 1 | Improve (1), Deteriorate (0), Unchanged (0), Unclear (0) | Yes (0), No (0), NR (1) | Yes (0), No (0), NR (1) |
| Vascular diseases | 1 | Improve (1), Deteriorate (0), Unchanged (0), Unclear (0) | Yes (0), No (0), NR (1) | Yes (0), No (0), NR (1) |
| hsCRP or hs-CRP | 4 | Improve (2), Deteriorate (0), Unchanged (2), Unclear (0) | Yes (2), No (2), NR (0) | Yes (2), No (2), NR (0) |

Table S17 Description of outcomes reported in Green tea Intervention studies

| **Outcome** | **N studies** | **Association with outcome** | **With-in arm significance** | **Between arm significance** |
| --- | --- | --- | --- | --- |
| ICAM-1 | 1 | Improve (0), Deteriorate (1), Unchanged (0), Unclear (0) | Yes (0), No (0), NR (1) | Yes (0), No (1), NR (0) |
| Diastolic blood pressure | 22 | Improve (14), Deteriorate (4), Unchanged (4), Unclear (0) | Yes (7), No (12), NR (3) | Yes (3), No (17), NR (2) |
| Fasting blood glucose | 22 | Improve (11), Deteriorate (2), Unchanged (8), Unclear (1) | Yes (3), No (15), NR (4) | Yes (1), No (16), NR (5) |
| Flow-mediated dilation (FMD) | 1 | Improve (1), Deteriorate (0), Unchanged (0), Unclear (0) | Yes (1), No (0), NR (0) | Yes (1), No (0), NR (0) |
| HDL | 22 | Improve (12), Deteriorate (6), Unchanged (3), Unclear (1) | Yes (6), No (9), NR (6) | Yes (3), No (17), NR (3) |
| HbA1C | 10 | Improve (4), Deteriorate (3), Unchanged (3), Unclear (1) | Yes (2), No (6), NR (3) | Yes (0), No (8), NR (3) |
| Insulin resistance or HOMA-IR | 7 | Improve (6), Deteriorate (0), Unchanged (1), Unclear (0) | Yes (3), No (4), NR (0) | Yes (1), No (5), NR (1) |
| Interleukin-1beta (IL-1B) | 1 | Improve (0), Deteriorate (1), Unchanged (0), Unclear (0) | Yes (0), No (0), NR (1) | Yes (0), No (1), NR (0) |
| Interleukin-6 (IL-6) | 1 | Improve (1), Deteriorate (0), Unchanged (0), Unclear (0) | Yes (0), No (0), NR (1) | Yes (0), No (1), NR (0) |
| LDL | 21 | Improve (15), Deteriorate (2), Unchanged (2), Unclear (2) | Yes (5), No (10), NR (5) | Yes (4), No (14), NR (4) |
| LDL/HDL or LDL:HDL ratio | 2 | Improve (2), Deteriorate (0), Unchanged (0), Unclear (0) | Yes (1), No (1), NR (0) | Yes (0), No (2), NR (0) |
| Oxidized low density lipoprotein | 2 | Improve (2), Deteriorate (0), Unchanged (0), Unclear (0) | Yes (0), No (1), NR (1) | Yes (1), No (1), NR (0) |
| Postprandial blood glucose | 2 | Improve (0), Deteriorate (0), Unchanged (2), Unclear (0) | Yes (0), No (2), NR (0) | Yes (0), No (1), NR (1) |
| Systolic blood pressure | 21 | Improve (15), Deteriorate (3), Unchanged (3), Unclear (0) | Yes (7), No (11), NR (3) | Yes (4), No (15), NR (2) |
| Total Cholesterol | 24 | Improve (17), Deteriorate (1), Unchanged (5), Unclear (1) | Yes (6), No (13), NR (5) | Yes (4), No (17), NR (3) |
| Triglycerides | 23 | Improve (12), Deteriorate (6), Unchanged (5), Unclear (0) | Yes (8), No (11), NR (4) | Yes (5), No (14), NR (4) |
| Tumor necrosis factor-alpha (TNF-alpha) | 1 | Improve (1), Deteriorate (0), Unchanged (0), Unclear (0) | Yes (1), No (0), NR (0) | Yes (1), No (0), NR (0) |
| VCAM-1 | 1 | Improve (0), Deteriorate (1), Unchanged (0), Unclear (0) | Yes (0), No (0), NR (1) | Yes (0), No (1), NR (0) |
| hsCRP or hs-CRP | 4 | Improve (1), Deteriorate (2), Unchanged (1), Unclear (0) | Yes (2), No (0), NR (2) | Yes (1), No (3), NR (0) |

Table S18 Description of outcomes reported in Mixed/other Intervention studies

| **Outcome** | **N studies** | **Association with outcome** | **With-in arm significance** | **Between arm significance** |
| --- | --- | --- | --- | --- |
| Diastolic blood pressure | 1 | Improve (1), Deteriorate (0), Unchanged (0), Unclear (0) | Yes (1), No (0), NR (0) | Yes (1), No (0), NR (0) |
| Fasting blood glucose | 1 | Improve (1), Deteriorate (0), Unchanged (0), Unclear (0) | Yes (1), No (0), NR (0) | Yes (0), No (1), NR (0) |
| HDL | 2 | Improve (1), Deteriorate (0), Unchanged (1), Unclear (0) | Yes (1), No (1), NR (0) | Yes (1), No (1), NR (0) |
| HbA1C | 2 | Improve (1), Deteriorate (0), Unchanged (1), Unclear (0) | Yes (0), No (2), NR (0) | Yes (0), No (2), NR (0) |
| Isoprostane | 1 | Improve (0), Deteriorate (0), Unchanged (1), Unclear (0) | Yes (0), No (1), NR (0) | Yes (0), No (1), NR (0) |
| LDL | 1 | Improve (0), Deteriorate (0), Unchanged (1), Unclear (0) | Yes (0), No (1), NR (0) | Yes (1), No (0), NR (0) |
| Systolic blood pressure | 1 | Improve (1), Deteriorate (0), Unchanged (0), Unclear (0) | Yes (1), No (0), NR (0) | Yes (1), No (0), NR (0) |
| Total Cholesterol | 2 | Improve (2), Deteriorate (0), Unchanged (0), Unclear (0) | Yes (1), No (1), NR (0) | Yes (2), No (0), NR (0) |
| Triglycerides | 2 | Improve (2), Deteriorate (0), Unchanged (0), Unclear (0) | Yes (2), No (0), NR (0) | Yes (1), No (1), NR (0) |
| hsCRP or hs-CRP | 2 | Improve (1), Deteriorate (0), Unchanged (1), Unclear (0) | Yes (2), No (0), NR (0) | Yes (0), No (2), NR (0) |

Table S19 Description of outcomes reported in Plums Intervention studies

| **Outcome** | **N studies** | **Association with outcome** | **With-in arm significance** | **Between arm significance** |
| --- | --- | --- | --- | --- |
| Diastolic blood pressure | 1 | Improve (1), Deteriorate (0), Unchanged (0), Unclear (0) | Yes (0), No (1), NR (0) | Yes (0), No (0), NR (1) |
| HDL | 1 | Improve (1), Deteriorate (0), Unchanged (0), Unclear (0) | Yes (1), No (0), NR (0) | Yes (0), No (0), NR (1) |
| LDL | 1 | Improve (1), Deteriorate (0), Unchanged (0), Unclear (0) | Yes (1), No (0), NR (0) | Yes (0), No (0), NR (1) |
| Systolic blood pressure | 1 | Improve (1), Deteriorate (0), Unchanged (0), Unclear (0) | Yes (1), No (0), NR (0) | Yes (0), No (0), NR (1) |
| Total Cholesterol | 1 | Improve (1), Deteriorate (0), Unchanged (0), Unclear (0) | Yes (1), No (0), NR (0) | Yes (0), No (0), NR (1) |
| Triglycerides | 1 | Improve (0), Deteriorate (0), Unchanged (1), Unclear (0) | Yes (0), No (1), NR (0) | Yes (0), No (0), NR (1) |

Table S20 Description of outcomes reported in Tea Intervention studies

| **Outcome** | **N studies** | **Association with outcome** | **With-in arm significance** | **Between arm significance** |
| --- | --- | --- | --- | --- |
| Diastolic blood pressure | 2 | Improve (1), Deteriorate (0), Unchanged (1), Unclear (0) | Yes (0), No (2), NR (0) | Yes (0), No (2), NR (0) |
| Endothelial dysfunction | 1 | Improve (1), Deteriorate (0), Unchanged (0), Unclear (0) | Yes (1), No (0), NR (0) | Yes (1), No (0), NR (0) |
| Fasting blood glucose | 3 | Improve (2), Deteriorate (0), Unchanged (1), Unclear (0) | Yes (1), No (2), NR (0) | Yes (1), No (0), NR (2) |
| Flow-mediated dilation (FMD) | 1 | Improve (1), Deteriorate (0), Unchanged (0), Unclear (0) | Yes (1), No (0), NR (0) | Yes (1), No (0), NR (0) |
| HDL | 5 | Improve (1), Deteriorate (1), Unchanged (3), Unclear (0) | Yes (2), No (3), NR (0) | Yes (1), No (2), NR (2) |
| HbA1C | 3 | Improve (2), Deteriorate (0), Unchanged (1), Unclear (0) | Yes (1), No (2), NR (0) | Yes (0), No (1), NR (2) |
| Interleukin-1beta (IL-1B) | 1 | Improve (1), Deteriorate (0), Unchanged (0), Unclear (0) | Yes (0), No (1), NR (0) | Yes (0), No (1), NR (0) |
| Interleukin-6 (IL-6) | 2 | Improve (2), Deteriorate (0), Unchanged (0), Unclear (0) | Yes (1), No (1), NR (0) | Yes (1), No (1), NR (0) |
| Isoprostane | 0 | Improve (0), Deteriorate (0), Unchanged (0), Unclear (0) | Yes (0), No (0), NR (0) | Yes (0), No (0), NR (0) |
| LDL | 4 | Improve (2), Deteriorate (0), Unchanged (2), Unclear (0) | Yes (1), No (3), NR (0) | Yes (1), No (1), NR (2) |
| Postprandial blood glucose | 1 | Improve (1), Deteriorate (0), Unchanged (0), Unclear (0) | Yes (1), No (0), NR (0) | Yes (1), No (0), NR (0) |
| Pulse wave analysis or pulse wave velocity | 2 | Improve (0), Deteriorate (0), Unchanged (1), Unclear (1) | Yes (0), No (2), NR (0) | Yes (0), No (2), NR (0) |
| Systolic blood pressure | 2 | Improve (1), Deteriorate (1), Unchanged (0), Unclear (0) | Yes (1), No (1), NR (0) | Yes (0), No (1), NR (1) |
| Total Cholesterol | 4 | Improve (2), Deteriorate (0), Unchanged (2), Unclear (0) | Yes (1), No (3), NR (0) | Yes (1), No (1), NR (2) |
| Triglycerides | 4 | Improve (1), Deteriorate (0), Unchanged (3), Unclear (0) | Yes (1), No (3), NR (0) | Yes (1), No (1), NR (2) |
| Tumor necrosis factor-alpha (TNF-alpha) | 1 | Improve (1), Deteriorate (0), Unchanged (0), Unclear (0) | Yes (1), No (0), NR (0) | Yes (1), No (0), NR (0) |
| hsCRP or hs-CRP | 1 | Improve (0), Deteriorate (1), Unchanged (0), Unclear (0) | Yes (0), No (1), NR (0) | Yes (0), No (1), NR (0) |

Table S21 Description of outcomes reported in Wine Intervention studies

| **Outcome** | **N studies** | **Association with outcome** | **With-in arm significance** | **Between arm significance** |
| --- | --- | --- | --- | --- |
| Diastolic blood pressure | 11 | Improve (5), Deteriorate (2), Unchanged (3), Unclear (1) | Yes (4), No (5), NR (2) | Yes (4), No (5), NR (2) |
| Endothelial dysfunction | 2 | Improve (1), Deteriorate (0), Unchanged (1), Unclear (0) | Yes (0), No (1), NR (1) | Yes (0), No (1), NR (1) |
| Fasting blood glucose | 11 | Improve (3), Deteriorate (1), Unchanged (6), Unclear (1) | Yes (1), No (7), NR (3) | Yes (2), No (5), NR (4) |
| Flow-mediated dilation (FMD) | 4 | Improve (1), Deteriorate (0), Unchanged (3), Unclear (0) | Yes (1), No (0), NR (3) | Yes (0), No (0), NR (4) |
| HDL | 17 | Improve (11), Deteriorate (2), Unchanged (4), Unclear (0) | Yes (8), No (5), NR (4) | Yes (7), No (5), NR (5) |
| HbA1C | 1 | Improve (0), Deteriorate (0), Unchanged (0), Unclear (0) | Yes (0), No (1), NR (0) | Yes (0), No (1), NR (0) |
| ICAM-1 | 2 | Improve (1), Deteriorate (1), Unchanged (0), Unclear (0) | Yes (1), No (1), NR (0) | Yes (1), No (0), NR (1) |
| Insulin resistance or HOMA-IR | 4 | Improve (2), Deteriorate (0), Unchanged (2), Unclear (0) | Yes (1), No (1), NR (2) | Yes (2), No (1), NR (1) |
| Interleukin-6 (IL-6) | 3 | Improve (2), Deteriorate (1), Unchanged (0), Unclear (0) | Yes (2), No (1), NR (0) | Yes (2), No (0), NR (1) |
| Isoprostane | 1 | Improve (0), Deteriorate (1), Unchanged (0), Unclear (0) | Yes (0), No (1), NR (0) | Yes (0), No (0), NR (1) |
| LDL | 15 | Improve (7), Deteriorate (2), Unchanged (5), Unclear (1) | Yes (2), No (7), NR (6) | Yes (3), No (8), NR (4) |
| LDL/HDL or LDL:HDL ratio | 2 | Improve (2), Deteriorate (0), Unchanged (0), Unclear (0) | Yes (2), No (0), NR (0) | Yes (0), No (2), NR (0) |
| Oxidized low density lipoprotein | 4 | Improve (1), Deteriorate (0), Unchanged (1), Unclear (1) | Yes (1), No (3), NR (0) | Yes (1), No (2), NR (1) |
| Postprandial blood glucose | 1 | Improve (0), Deteriorate (0), Unchanged (1), Unclear (0) | Yes (0), No (1), NR (0) | Yes (0), No (1), NR (0) |
| Pulse wave analysis or pulse wave velocity | 2 | Improve (0), Deteriorate (0), Unchanged (2), Unclear (0) | Yes (0), No (2), NR (0) | Yes (0), No (2), NR (0) |
| Quantitative Insulin Sensitivity Check Index (QUICKI) | 1 | Improve (0), Deteriorate (0), Unchanged (1), Unclear (0) | Yes (0), No (1), NR (0) | Yes (0), No (1), NR (0) |
| Systolic blood pressure | 11 | Improve (5), Deteriorate (2), Unchanged (3), Unclear (1) | Yes (4), No (5), NR (2) | Yes (4), No (5), NR (2) |
| Total Cholesterol | 14 | Improve (4), Deteriorate (7), Unchanged (2), Unclear (1) | Yes (2), No (8), NR (4) | Yes (1), No (8), NR (5) |
| Triglycerides | 16 | Improve (4), Deteriorate (7), Unchanged (4), Unclear (1) | Yes (3), No (7), NR (6) | Yes (2), No (8), NR (6) |
| Tumor necrosis factor-alpha (TNF-alpha) | 4 | Improve (1), Deteriorate (1), Unchanged (1), Unclear (1) | Yes (0), No (4), NR (0) | Yes (1), No (1), NR (2) |
| VCAM-1 | 2 | Improve (1), Deteriorate (1), Unchanged (0), Unclear (0) | Yes (1), No (1), NR (0) | Yes (0), No (1), NR (1) |
| hsCRP or hs-CRP | 4 | Improve (2), Deteriorate (0), Unchanged (2), Unclear (0) | Yes (0), No (4), NR (0) | Yes (1), No (0), NR (3) |

Reference List of Included Randomized Controlled Trials

|  | **Authors** | **Title** | **Journal** | **Pubmed id** |
| --- | --- | --- | --- | --- |
| 1 | Ahmed,T. and Sadia,H. and Batool,S. and Janjua,A. and Shuja,F. | Use of prunes as a control of hypertension | J Ayub Med Coll Abbottabad. 2010 Jan-Mar;22(1):28-31. | 21409897 |
| 2 | Akilen,R. and Tsiami,A. and Devendra,D. and Robinson,N. | Glycated haemoglobin and blood pressure-lowering effect of cinnamon in multi-ethnic Type 2 diabetic patients in the UK: a randomized, placebo-controlled, double-blind clinical trial | Diabet Med. 2010 Oct;27(10):1159-67. | 20854384 |
| 3 | Alexopoulos,N. and Vlachopoulos,C. and Aznaouridis,K. and Baou,K. and Vasiliadou,C. and Pietri,P. and Xaplanteris,P. and Stefanadi,E. and Stefanadis,C. | The acute effect of green tea consumption on endothelial function in healthy individuals | Eur J Cardiovasc Prev Rehabil. 2008 Jun;15(3):300-5. | 18525384 |
| 4 | Almoosawi S and Tsang C and Ostertag LM and Fyfe L and Al-Dujaili EA | Differential effect of polyphenol-rich dark chocolate on biomarkers of glucose metabolism and cardiovascular risk factors in healthy, overweight and obese subjects: a randomized clinical trial. | Food Funct. 2012 Oct;3(10):1035-43. Epub 2012 Jul 16. | 22796902 |
| 5 | Almoosawi,S. and Fyfe,L. and Ho,C. and Al-Dujaili,E. | The effect of polyphenol-rich dark chocolate on fasting capillary whole blood glucose, total cholesterol, blood pressure and glucocorticoids in healthy overweight and obese subjects | Br J Nutr. 2010 Mar;103(6):842-50. | 19825207 |
| 6 | Aro,A. and Kostiainen,E. and Huttunen,J.K. and Seppala,E. and Vapaatalo,H. | Effects of coffee and tea on lipoproteins and prostanoids | Atherosclerosis. 1985 Oct;57(1):123-8. | 4074461 |
| 7 | Asher GN and Viera AJ and Weaver MA and Dominik R and Caughey M and Hinderliter AL | Effect of hawthorn standardized extract on flow mediated dilation in prehypertensive and mildly hypertensive adults: a randomized, controlled cross-over trial. | BMC Complement Altern Med. 2012 Mar 29;12:26. | 22458601 |
| 8 | Askari F and Rashidkhani B and Hekmatdoost A | Cinnamon may have therapeutic benefits on lipid profile, liver enzymes, insulin resistance, and high-sensitivity C-reactive protein in nonalcoholic fatty liver disease patients. | Nutr Res. 2014 Feb;34(2):143-8. | 24461315 |
| 9 | Auclair,S. and Chironi,G. and Milenkovic,D. and Hollman,P.C. and Renard,C.M. and Megnien,J.L. and Gariepy,J. and Paul,J.L. and Simon,A. and Scalbert,A. | The regular consumption of a polyphenol-rich apple does not influence endothelial function: a randomised double-blind trial in hypercholesterolemic adults | Eur J Clin Nutr. 2010 Oct;64(10):1158-65. | 20683465 |
| 10 | Azimi,P. and Ghiasvand,R. and Feizi,A. and Hariri,M. and Abbasi,B. | Effects of Cinnamon, Cardamom, Saffron, and Ginger Consumption on Markers of Glycemic Control, Lipid Profile, Oxidative Stress, and Inflammation in Type 2 Diabetes Patients | Rev Diabet Stud. 2014 Fall-Winter;11(3-4):258-66. | 26177486 |
| 11 | Baba,S. and Natsume,M. and Yasuda,A. and Nakamura,Y. and Tamura,T. and Osakabe,N. and Kanegae,M. and Kondo,K. | Plasma LDL and HDL cholesterol and oxidized LDL concentrations are altered in normo- and hypercholesterolemic humans after intake of different levels of cocoa powder | J Nutr. 2007 Jun;137(6):1436-41. | 17513403 |
| 12 | Bahorun,T. and Luximon-Ramma,A. and Neergheen-Bhujun,V.S. and Gunness,T.K. and Googoolye,K. and Auger,C. and Crozier,A. and Aruoma,O.I. | The effect of black tea on risk factors of cardiovascular disease in a normal population | Prev Med. 2012 May;54 Suppl:S98-102. | 22198621 |
| 13 | Balzer,J. and Rassaf,T. and Heiss,C. and Kleinbongard,P. and Lauer,T. and Merx,M. and Heussen,N. and Gross,H.B. and Keen,C.L. and Schroeter,H. and Kelm,M. | Sustained benefits in vascular function through flavanol-containing cocoa in medicated diabetic patients a double-masked, randomized, controlled trial | J Am Coll Cardiol. 2008 Jun 3;51(22):2141-9. | 18510961 |
| 14 | Banach,J. and Zekanowska,E. and Bujak,R. and Gilewski,W. and Blazejewski,J. and Karasek,D. and Balak,W. and Pietrzak,J. and Sinkiewicz,W. | Short-term alcohol consumption may have detrimental effect on fibrinolysis and endothelial function: preliminary report of prospective randomised study | Kardiol Pol. 2013;71(11):1161-7. | 23633272 |
| 15 | Barden,A.E. and Croft,K.D. and Beilin,L.J. and Phillips,M. and Ledowski,T. and Puddey,I.B. | Acute effects of red wine on cytochrome P450 eicosanoids and blood pressure in men | J Hypertens. 2013 Nov;31(11):2195-202; discussion 2202. | 24096258 |
| 16 | Barona J and Aristizabal JC and Blesso CN and Volek JS and Fernandez ML | Grape polyphenols reduce blood pressure and increase flow-mediated vasodilation in men with metabolic syndrome. | J Nutr. 2012 Sep;142(9):1626-32. | 22810991 |
| 17 | Basu,A. and Betts,N.M. and Nguyen,A. and Newman,E.D. and Fu,D. and Lyons,T.J. | Freeze-dried strawberries lower serum cholesterol and lipid peroxidation in adults with abdominal adiposity and elevated serum lipids | J Nutr. 2014 Jun;144(6):830-7. | 24670970 |
| 18 | Basu,A. and Betts,N.M. and Ortiz,J. and Simmons,B. and Wu,M. and Lyons,T.J. | Low-energy cranberry juice decreases lipid oxidation and increases plasma antioxidant capacity in women with metabolic syndrome | Nutr Res. 2011 Mar;31(3):190-6. | 21481712 |
| 19 | Basu,A. and Du,M. and Leyva,M.J. and Sanchez,K. and Betts,N.M. and Wu,M. and Aston,C.E. and Lyons,T.J. | Blueberries decrease cardiovascular risk factors in obese men and women with metabolic syndrome | J Nutr. 2010 Sep;140(9):1582-7. | 20660279 |
| 20 | Basu,A. and Du,M. and Sanchez,K. and Leyva,M.J. and Betts,N.M. and Blevins,S. and Wu,M. and Aston,C.E. and Lyons,T.J. | Green tea minimally affects biomarkers of inflammation in obese subjects with metabolic syndrome | Nutrition. 2011 Feb;27(2):206-13. | 20605696 |
| 21 | Basu,A. and Fu,D.X. and Wilkinson,M. and Simmons,B. and Wu,M. and Betts,N.M. and Du,M. and Lyons,T.J. | Strawberries decrease atherosclerotic markers in subjects with metabolic syndrome | Nutr Res. 2010 Jul;30(7):462-9. | 20797478 |
| 22 | Basu,A. and Sanchez,K. and Leyva,M.J. and Wu,M. and Betts,N.M. and Aston,C.E. and Lyons,T.J. | Green tea supplementation affects body weight, lipids, and lipid peroxidation in obese subjects with metabolic syndrome | J Am Coll Nutr. 2010 Feb;29(1):31-40. | 20595643 |
| 23 | Batista,Gde A. and Cunha,C.L. and Scartezini,M. and von der,Heyde R. and Bitencourt,M.G. and Melo,S.F. | Prospective double-blind crossover study of Camellia sinensis (green tea) in dyslipidemias | Arq Bras Cardiol. 2009 Aug;93(2):128-34. | 19838489 |
| 24 | Belcaro G, Ledda A, Hu S, Cesarone MR, Feragalli B, Dugall M. | Grape seed procyanidins in pre- and mild hypertension: a registry study. | Evid Based Complement Alternat Med. 2013;2013:313142. | 24171039 |
| 25 | Belcaro G, Ledda A, Hu S, Cesarone MR, Feragalli B, Dugall M. | Greenselect phytosome for borderline metabolic syndrome | Evid Based Complement Alternat Med. 2013;2013:869061. | 24348726 |
| 26 | Bertipaglia de,Santana M. and Mandarino,M.G. and Cardoso,J.R. and Dichi,I. and Dichi,J.B. and Camargo,A.E. and Fabris,B.A. and Rodrigues,R.J. and Fatel,E.C. and Nixdorf,S.L. and Simao,A.N. and Cecchini,R. and Barbosa,D.S. | Association between soy and green tea (Camellia sinensis) diminishes hypercholesterolemia and increases total plasma antioxidant potential in dyslipidemic subjects | Nutrition. 2008 Jun;24(6):562-8. | 18455656 |
| 27 | Bingham,S.A. and Vorster,H. and Jerling,J.C. and Magee,E. and Mulligan,A. and Runswick,S.A. and Cummings,J.H. | Effect of black tea drinking on blood lipids, blood pressure and aspects of bowel habit | Br J Nutr. 1997 Jul;78(1):41-55. | 9292758 |
| 28 | Blevins,S.M. and Leyva,M.J. and Brown,J. and Wright,J. and Scofield,R.H. and Aston,C.E. | Effect of cinnamon on glucose and lipid levels in non insulin-dependent type 2 diabetes | Diabetes Care. 2007 Sep;30(9):2236-7. Epub 2007 Jun 11. | 17563345 |
| 29 | Bogdanski,P. and Suliburska,J. and Szulinska,M. and Stepien,M. and Pupek-Musialik,D. and Jablecka,A. | Green tea extract reduces blood pressure, inflammatory biomarkers, and oxidative stress and improves parameters associated with insulin resistance in obese, hypertensive patients | Nutr Res. 2012 Jun;32(6):421-7. | 22749178 |
| 30 | Bohn,S.K. and Croft,K.D. and Burrows,S. and Puddey,I.B. and Mulder,T.P. and Fuchs,D. and Woodman,R.J. and Hodgson,J.M. | Effects of black tea on body composition and metabolic outcomes related to cardiovascular disease risk: a randomized controlled trial | Food Funct. 2014 Jul 25;5(7):1613-20. | 24889137 |
| 31 | Bondonno,C.P. and Yang,X. and Croft,K.D. and Considine,M.J. and Ward,N.C. and Rich,L. and Puddey,I.B. and Swinny,E. and Mubarak,A. and Hodgson,J.M. | Flavonoid-rich apples and nitrate-rich spinach augment nitric oxide status and improve endothelial function in healthy men and women: a randomized controlled trial | Free Radic Biol Med. 2012 Jan 1;52(1):95-102. | 22019438 |
| 32 | Brown,A.L. and Lane,J. and Coverly,J. and Stocks,J. and Jackson,S. and Stephen,A. and Bluck,L. and Coward,A. and Hendrickx,H. | Effects of dietary supplementation with the green tea polyphenol epigallocatechin-3-gallate on insulin resistance and associated metabolic risk factors: randomized controlled trial | Br J Nutr. 2009 Mar;101(6):886-94. | 18710606 |
| 33 | Brown,A.L. and Lane,J. and Holyoak,C. and Nicol,B. and Mayes,A.E. and Dadd,T. | Health effects of green tea catechins in overweight and obese men: a randomised controlled cross-over trial | Br J Nutr. 2011 Dec;106(12):1880-9. | 21736785 |
| 34 | Bulut,D. and Jelich,U. and Dacanay-Schwarz,R. and Mugge,A. | Red wine ingestion prevents microparticle formation after a single high-fat meal--a crossover study in healthy humans | J Cardiovasc Pharmacol. 2013 Jun;61(6):489-94. | 23429592 |
| 35 | Burton-Freeman,B. and Linares,A. and Hyson,D. and Kappagoda,T. | Strawberry modulates LDL oxidation and postprandial lipemia in response to high-fat meal in overweight hyperlipidemic men and women | J Am Coll Nutr. 2010 Feb;29(1):46-54. | 20595645 |
| 36 | Cao,A.H. and Wang,J. and Gao,H.Q. and Zhang,P. and Qiu,J. | Beneficial clinical effects of grape seed proanthocyanidin extract on the progression of carotid atherosclerotic plaques | doi: 10.11909/j.issn.1671-5411.2015.04.014. | 26345394 |
| 37 | Chiva-Blanch,G. and Urpi-Sarda,M. and Llorach,R. and Rotches-Ribalta,M. and Guillen,M. and Casas,R. and Arranz,S. and Valderas-Martinez,P. and Portoles,O. and Corella,D. and Tinahones,F. and Lamuela-Raventos,R.M. and Andres-Lacueva,C. and Estruch,R. | Differential effects of polyphenols and alcohol of red wine on the expression of adhesion molecules and inflammatory cytokines related to atherosclerosis: a randomized clinical trial.[Erratum appears in Am J Clin Nutr. 2012 Jun;95(6):1506] | Am J Clin Nutr. 2012 Feb;95(2):326-34. | 22205309 |
| 38 | Chiva-Blanch,G. and Urpi-Sarda,M. and Ros,E. and Arranz,S. and Valderas-Martinez,P. and Casas,R. and Sacanella,E. and Llorach,R. and Lamuela-Raventos,R.M. and Andres-Lacueva,C. and Estruch,R. | Dealcoholized red wine decreases systolic and diastolic blood pressure and increases plasma nitric oxide: short communication | Circ Res. 2012 Sep 28;111(8):1065-8. Epub 2012 Sep 6. | 22955728 |
| 39 | Chiva-Blanch,G. and Urpi-Sarda,M. and Ros,E. and Valderas-Martinez,P. and Casas,R. and Arranz,S. and Guillen,M. and Lamuela-Raventos,R.M. and Llorach,R. and Andres-Lacueva,C. and Estruch,R. | Effects of red wine polyphenols and alcohol on glucose metabolism and the lipid profile: a randomized clinical trial | Clin Nutr. 2013 Apr;32(2):200-6. | 22999066 |
| 40 | Chu,S.L. and Fu,H. and Yang,J.X. and Liu,G.X. and Dou,P. and Zhang,L. and Tu,P.F. and Wang,X.M. | A randomized double-blind placebo-controlled study of Pu'er tea extract on the regulation of metabolic syndrome | Chin J Integr Med. 2011 Jul;17(7):492-8. | 21725873 |
| 41 | Cordain,L. and Melby,C.L. and Hamamoto,A.E. and O'Neill,D.S. and Cornier,M.A. and Barakat,H.A. and Israel,R.G. and Hill,J.O. | Influence of moderate chronic wine consumption on insulin sensitivity and other correlates of syndrome X in moderately obese women | Metabolism. 2000 Nov;49(11):1473-8. | 11092514 |
| 42 | Crawford,P. | Effectiveness of cinnamon for lowering hemoglobin A1C in patients with type 2 diabetes: a randomized, controlled trial | J Am Board Fam Med. 2009 Sep-Oct;22(5):507-12. | 19734396 |
| 43 | Crews,W.D.,Jr. and Harrison,D.W. and Wright,J.W. | A double-blind, placebo-controlled, randomized trial of the effects of dark chocolate and cocoa on variables associated with neuropsychological functioning and cardiovascular health: clinical findings from a sample of healthy, cognitively intact older adults | Am J Clin Nutr. 2008 Apr;87(4):872-80. | 18400709 |
| 44 | Curtis,P.J. and Potter,J. and Kroon,P.A. and Wilson,P. and Dhatariya,K. and Sampson,M. and Cassidy,A. | Vascular function and atherosclerosis progression after 1 y of flavonoid intake in statin-treated postmenopausal women with type 2 diabetes: a double-blind randomized controlled trial | Am J Clin Nutr. 2013 May;97(5):936-42. | 23553151 |
| 45 | Curtis,P.J. and Sampson,M. and Potter,J. and Dhatariya,K. and Kroon,P.A. and Cassidy,A. | Chronic ingestion of flavan-3-ols and isoflavones improves insulin sensitivity and lipoprotein status and attenuates estimated 10-year CVD risk in medicated postmenopausal women with type 2 diabetes: a 1-year, double-blind, randomized, controlled trial | Diabetes Care. 2012 Feb;35(2):226-32. | 22250063 |
| 46 | Davison,K. and Berry,N.M. and Misan,G. and Coates,A.M. and Buckley,J.D. and Howe,P.R. | Dose-related effects of flavanol-rich cocoa on blood pressure | J Hum Hypertens. 2010 Sep;24(9):568-76. | 20090776 |
| 47 | Davison,K. and Coates,A.M. and Buckley,J.D. and Howe,P.R. | Effect of cocoa flavanols and exercise on cardiometabolic risk factors in overweight and obese subjects | Int J Obes (Lond). 2008 Aug;32(8):1289-96. | 18504447 |
| 48 | de Jesus Romero-Prado MM and Curiel-Beltran,J.A. and Miramontes-Espino,M.V. and Cardona-Munoz,E.G. and Rios-Arellano,A. and Balam-Salazar,L.B. | Dietary flavonoids added to pharmacological antihypertensive therapy are effective in improving blood pressure | Basic Clin Pharmacol Toxicol. 2015 Jul;117(1):57-64. | 25441094 |
| 49 | de Rijke,Y.B. and Demacker,P.N. and Assen,N.A. and Sloots,L.M. and Katan,M.B. and Stalenhoef,A.F. | Red wine consumption does not affect oxidizability of low-density lipoproteins in volunteers | Am J Clin Nutr. 1996 Mar;63(3):329-34. | 8602588 |
| 50 | Del,Bo C. and Riso,P. and Campolo,J. and Moller,P. and Loft,S. and Klimis-Zacas,D. and Brambilla,A. and Rizzolo,A. and Porrini,M. | A single portion of blueberry (Vaccinium corymbosum L) improves protection against DNA damage but not vascular function in healthy male volunteers | Nutr Res. 2013 Mar;33(3):220-7. | 23507228 |
| 51 | Desch,S. and Kobler,D. and Schmidt,J. and Sonnabend,M. and Adams,V. and Sareban,M. and Eitel,I. and Bluher,M. and Schuler,G. and Thiele,H. | Low vs. higher-dose dark chocolate and blood pressure in cardiovascular high-risk patients | Am J Hypertens. 2010 Jun;23(6):694-700. | 20203627 |
| 52 | Di Renzo L and Carraro A and Valente R and Iacopino L and Colica C and De Lorenzo A | Intake of red wine in different meals modulates oxidized LDL level, oxidative and inflammatory gene expression in healthy people: a randomized crossover trial. | Oxid Med Cell Longev. 2014;2014:681318. | 24876915 |
| 53 | Di,Pierro F. and Menghi,A.B. and Barreca,A. and Lucarelli,M. and Calandrelli,A. | Greenselect Phytosome as an adjunct to a low-calorie diet for treatment of obesity: a clinical trial | Altern Med Rev. 2009 Jun;14(2):154-60. | 19594224 |
| 54 | Djurovic,S. and Berge,K.E. and Birkenes,B. and Braaten,O. and Retterstol,L. | The effect of red wine on plasma leptin levels and vasoactive factors from adipose tissue: a randomized crossover trial | Alcohol Alcohol. 2007 Nov-Dec;42(6):525-8. Epub 2007 Aug 1. | 17670801 |
| 55 | Dohadwala,M.M. and Hamburg,N.M. and Holbrook,M. and Kim,B.H. and Duess,M.A. and Levit,A. and Titas,M. and Chung,W.B. and Vincent,F.B. and Caiano,T.L. and Frame,A.A. and Keaney,J.F.,Jr. and Vita,J.A. | Effects of Concord grape juice on ambulatory blood pressure in prehypertension and stage 1 hypertension | Am J Clin Nutr. 2010 Nov;92(5):1052-9. | 20844075 |
| 56 | Dohadwala,M.M. and Holbrook,M. and Hamburg,N.M. and Shenouda,S.M. and Chung,W.B. and Titas,M. and Kluge,M.A. and Wang,N. and Palmisano,J. and Milbury,P.E. and Blumberg,J.B. and Vita,J.A. | Effects of cranberry juice consumption on vascular function in patients with coronary artery disease | Am J Clin Nutr. 2011 May;93(5):934-40. | 21411615 |
| 57 | Dower,J.I. and Geleijnse,J.M. and Gijsbers,L. and Schalkwijk,C. and Kromhout,D. and Hollman,P.C. | Supplementation of the Pure Flavonoids Epicatechin and Quercetin Affects Some Biomarkers of Endothelial Dysfunction and Inflammation in (Pre)Hypertensive Adults: A Randomized Double-Blind, Placebo-Controlled, Crossover Trial | J Nutr. 2015 Jul;145(7):1459-63. | 25972527 |
| 58 | Dower,J.I. and Geleijnse,J.M. and Gijsbers,L. and Zock,P.L. and Kromhout,D. and Hollman,P.C. | Effects of the pure flavonoids epicatechin and quercetin on vascular function and cardiometabolic health: a randomized, double-blind, placebo-controlled, crossover trial | Am J Clin Nutr. 2015 May;101(5):914-21. | 25934864 |
| 59 | Draijer,R. and de,Graaf Y. and Slettenaar,M. and de,Groot E. and Wright,C.I. | Consumption of a polyphenol-rich grape-wine extract lowers ambulatory blood pressure in mildly hypertensive subjects | Nutrients. 2015 Apr 30;7(5):3138-53. | 25942487 |
| 60 | Duffy,S.J. and Keaney,J.F.,Jr. and Holbrook,M. and Gokce,N. and Swerdloff,P.L. and Frei,B. and Vita,J.A. | Short- and long-term black tea consumption reverses endothelial dysfunction in patients with coronary artery disease | Circulation. 2001 Jul 10;104(2):151-6. | 11447078 |
| 61 | Ellis,C.L. and Edirisinghe,I. and Kappagoda,T. and Burton-Freeman,B. | Attenuation of meal-induced inflammatory and thrombotic responses in overweight men and women after 6-week daily strawberry (Fragaria) intake. A randomized placebo-controlled trial | J Atheroscler Thromb. 2011;18(4):318-27. Epub 2011 Jan 13. | 21242652 |
| 62 | Engler MB1, Engler MM, Chen CY, Malloy MJ, Browne A, Chiu EY, Kwak HK, Milbury P, Paul SM, Blumberg J, Mietus-Snyder ML. | Flavonoid-rich dark chocolate improves endothelial function and increases plasma epicatechin concentrations in healthy adults. | J Am Coll Nutr. 2004 Jun;23(3):197-204. | 15190043 |
| 63 | Erlund,I. and Koli,R. and Alfthan,G. and Marniemi,J. and Puukka,P. and Mustonen,P. and Mattila,P. and Jula,A. | Favorable effects of berry consumption on platelet function, blood pressure, and HDL cholesterol | Am J Clin Nutr. 2008 Feb;87(2):323-31. | 18258621 |
| 64 | Esser,D. and Mars,M. and Oosterink,E. and Stalmach,A. and Muller,M. and Afman,L.A. | Dark chocolate consumption improves leukocyte adhesion factors and vascular function in overweight men | FASEB J. 2014 Mar;28(3):1464-73. | 24302679 |
| 65 | Estruch R and Sacanella E and Mota F and Chiva-Blanch G and Antunez E and Casals E and Deulofeu R and Rotilio D and Andres-Lacueva C and Lamuela-Raventos RM and de Gaetano G and Urbano-Marquez A | Moderate consumption of red wine, but not gin, decreases erythrocyte superoxide dismutase activity: a randomised cross-over trial. | Nutr Metab Cardiovasc Dis. 2011 Jan;21(1):46-53. | 19819677 |
| 66 | Faridi,Z. and Njike,V.Y. and Dutta,S. and Ali,A. and Katz,D.L. | Acute dark chocolate and cocoa ingestion and endothelial function: a randomized controlled crossover trial | Am J Clin Nutr. 2008 Jul;88(1):58-63. | 18614724 |
| 67 | Farouque HM1, Leung M, Hope SA, Baldi M, Schechter C, Cameron JD, Meredith IT. | Acute and chronic effects of flavanol-rich cocoa on vascular function in subjects with coronary artery disease: a randomized double-blind placebo-controlled study. | Clin Sci (Lond). 2006 Jul;111(1):71-80. | 16551272 |
| 68 | Flammer,A.J. and Martin,E.A. and Gossl,M. and Widmer,R.J. and Lennon,R.J. and Sexton,J.A. and Loeffler,D. and Khosla,S. and Lerman,L.O. and Lerman,A. | Polyphenol-rich cranberry juice has a neutral effect on endothelial function but decreases the fraction of osteocalcin-expressing endothelial progenitor cells | Eur J Nutr. 2013 Feb;52(1):289-96. | 22382203 |
| 69 | Flammer,A.J. and Sudano,I. and Wolfrum,M. and Thomas,R. and Enseleit,F. and Periat,D. and Kaiser,P. and Hirt,A. and Hermann,M. and Serafini,M. and Leveques,A. and Luscher,T.F. and Ruschitzka,F. and Noll,G. and Corti,R. | Cardiovascular effects of flavanol-rich chocolate in patients with heart failure | Eur Heart J. 2012 Sep;33(17):2172-80. | 22173910 |
| 70 | Frank,J. and George,T.W. and Lodge,J.K. and Rodriguez-Mateos,A.M. and Spencer,J.P. and Minihane,A.M. and Rimbach,G. | Daily consumption of an aqueous green tea extract supplement does not impair liver function or alter cardiovascular disease risk biomarkers in healthy men | J Nutr. 2009 Jan;139(1):58-62. | 19056646 |
| 71 | Freese,R. and Basu,S. and Hietanen,E. and Nair,J. and Nakachi,K. and Bartsch,H. and Mutanen,M. | Green tea extract decreases plasma malondialdehyde concentration but does not affect other indicators of oxidative stress, nitric oxide production, or hemostatic factors during a high-linoleic acid diet in healthy females | Eur J Nutr. 1999 Jun;38(3):149-57. | 10443337 |
| 72 | Fuchs D and de Graaf Y and van Kerckhoven R and Draijer R | Effect of tea theaflavins and catechins on microvascular function. | Nutrients. 2014 Dec 11;6(12):5772-85. | 25514559 |
| 73 | Fujita,H. and Yamagami,T. | Antihypercholesterolemic effect of Chinese black tea extract in human subjects with borderline hypercholesterolemia | Nutr Res. 2008 Jul;28(7):450-6. | 19083445 |
| 74 | Fukino,Y. and Ikeda,A. and Maruyama,K. and Aoki,N. and Okubo,T. and Iso,H. | Randomized controlled trial for an effect of green tea-extract powder supplementation on glucose abnormalities | Eur J Clin Nutr. 2008 Aug;62(8):953-60. Epub 2007 Jun 6. | 17554248 |
| 75 | Gepner,Y. and Golan,R. and Harman-Boehm,I. and Henkin,Y. and Schwarzfuchs,D. and Shelef,I. and Durst,R. and Kovsan,J. and Bolotin,A. and Leitersdorf,E. and Shpitzen,S. and Balag,S. and Shemesh,E. and Witkow,S. and Tangi-Rosental,O. and Chassidim,Y. and Liberty,I.F. and Sarusi,B. and Ben-Avraham,S. and Helander,A. and Ceglarek,U. and Stumvoll,M. and Bluher,M. and Thiery,J. and Rudich,A. and Stampfer,M.J. and Shai,I. | Effects of Initiating Moderate Alcohol Intake on Cardiometabolic Risk in Adults With Type 2 Diabetes: A 2-Year Randomized, Controlled Trial.[Summary for patients in Ann Intern Med. 2015 Oct 20;163(8):I-34; PMID: 26457408] | Ann Intern Med. 2015 Oct 20;163(8):569-79. | 26458258 |
| 76 | Goldberg,D.M. and Garovic-Kocic,V. and Diamandis,E.P. and Pace-Asciak,C.R. | Wine: does the colour count? | Clin Chim Acta. 1996 Mar 15;246(1-2):183-93. | 8814966 |
| 77 | Grassi D and Draijer R and Desideri G and Mulder T and Ferri C | Black tea lowers blood pressure and wave reflections in fasted and postprandial conditions in hypertensive patients: a randomised study. | Nutrients. 2015 Feb 4;7(2):1037-51. | 25658240 |
| 78 | Grassi D, Necozione S, Lippi C, Croce G, Valeri L, Pasqualetti P, Desideri G, Blumberg JB, Ferri C. | Cocoa reduces blood pressure and insulin resistance and improves endothelium-dependent vasodilation in hypertensives. | Hypertension. 2005 Aug;46(2):398-405. Epub 2005 Jul 18. | 16027246 |
| 79 | Grassi,D. and Desideri,G. and Necozione,S. and di,Giosia P. and Barnabei,R. and Allegaert,L. and Bernaert,H. and Ferri,C. | Cocoa consumption dose-dependently improves flow-mediated dilation and arterial stiffness decreasing blood pressure in healthy individuals | J Hypertens. 2015 Feb;33(2):294-303. | 25380152 |
| 80 | Grassi,D. and Desideri,G. and Necozione,S. and Lippi,C. and Casale,R. and Properzi,G. and Blumberg,J.B. and Ferri,C. | Blood pressure is reduced and insulin sensitivity increased in glucose-intolerant, hypertensive subjects after 15 days of consuming high-polyphenol dark chocolate | J Nutr. 2008 Sep;138(9):1671-6. | 18716168 |
| 81 | Grassi,D. and Desideri,G. and Necozione,S. and Ruggieri,F. and Blumberg,J.B. and Stornello,M. and Ferri,C. | Protective effects of flavanol-rich dark chocolate on endothelial function and wave reflection during acute hyperglycemia | Hypertension. 2012 Sep;60(3):827-32. | 22851734 |
| 82 | Hammer,A. and Koppensteiner,R. and Steiner,S. and Niessner,A. and Goliasch,G. and Gschwandtner,M. and Hoke,M. | Dark chocolate and vascular function in patients with peripheral artery disease: a randomized, controlled cross-over trial | Clin Hemorheol Microcirc. 2015;59(2):145-53. | 24531123 |
| 83 | Hasanzade,F. and Toliat,M. and Emami,S.A. and Emamimoghaadam,Z. | The Effect of Cinnamon on Glucose of Type II Diabetes Patients | J Tradit Complement Med. 2013 Jul;3(3):171-4. | 24716174 |
| 84 | Heiss,C. and Jahn,S. and Taylor,M. and Real,W.M. and Angeli,F.S. and Wong,M.L. and Amabile,N. and Prasad,M. and Rassaf,T. and Ottaviani,J.I. and Mihardja,S. and Keen,C.L. and Springer,M.L. and Boyle,A. and Grossman,W. and Glantz,S.A. and Schroeter,H. and Yeghiazarians,Y. | Improvement of endothelial function with dietary flavanols is associated with mobilization of circulating angiogenic cells in patients with coronary artery disease | J Am Coll Cardiol. 2010 Jul 13;56(3):218-24. | 20620742 |
| 85 | Heiss,C. and Sansone,R. and Karimi,H. and Krabbe,M. and Schuler,D. and Rodriguez-Mateos,A. and Kraemer,T. and Cortese-Krott,M.M. and Kuhnle,G.G. and Spencer,J.P. and Schroeter,H. and Merx,M.W. and Kelm,M. and FLAVIOLA Consortium, | Impact of cocoa flavanol intake on age-dependent vascular stiffness in healthy men: a randomized, controlled, double-masked trial | Age (Dordr). 2015 Jun;37(3):9794. | 26013912 |
| 86 | Hodgson,J.M. and Croft,K.D. and Woodman,R.J. and Puddey,I.B. and Fuchs,D. and Draijer,R. and Lukoshkova,E. and Head,G.A. | Black tea lowers the rate of blood pressure variation: a randomized controlled trial | Am J Clin Nutr. 2013 May;97(5):943-50. | 23553154 |
| 87 | Hodgson,J.M. and Puddey,I.B. and Burke,V. and Beilin,L.J. and Jordan,N. | Effects on blood pressure of drinking green and black tea | J Hypertens. 1999 Apr;17(4):457-63. | 10404946 |
| 88 | Hodgson,J.M. and Puddey,I.B. and Mori,T.A. and Burke,V. and Baker,R.I. and Beilin,L.J. | Effects of regular ingestion of black tea on haemostasis and cell adhesion molecules in humans | Eur J Clin Nutr. 2001 Oct;55(10):881-6. | 11593350 |
| 89 | Hodgson,J.M. and Woodman,R.J. and Puddey,I.B. and Mulder,T. and Fuchs,D. and Croft,K.D. | Short-term effects of polyphenol-rich black tea on blood pressure in men and women | Food Funct. 2013 Jan;4(1):111-5. | 23038021 |
| 90 | Hoehn,A.N. and Stockert,A.L. | The Effects of Cinnamomum Cassia on Blood Glucose Values are Greater than those of Dietary Changes Alone | Nutr Metab Insights. 2012 Dec 13;5:77-83. | 23882151 |
| 91 | Hokayem M and Blond E and Vidal H and Lambert K and Meugnier E and Feillet-Coudray C and Coudray C and Pesenti S and Luyton C and Lambert-Porcheron S and Sauvinet V and Fedou C and Brun JF and Rieusset J and Bisbal C and Sultan A and Mercier J and Goudable J and Dupuy AM and Cristol JP and Laville M and Avignon A | Grape polyphenols prevent fructose-induced oxidative stress and insulin resistance in first-degree relatives of type 2 diabetic patients. | Diabetes Care. 2013 Jun;36(6):1454-61. | 23275372 |
| 92 | Hollis,J.H. and Houchins,J.A. and Blumberg,J.B. and Mattes,R.D. | Effects of concord grape juice on appetite, diet, body weight, lipid profile, and antioxidant status of adults | J Am Coll Nutr. 2009 Oct;28(5):574-82. | 20439553 |
| 93 | Hsu,C.H. and Liao,Y.L. and Lin,S.C. and Tsai,T.H. and Huang,C.J. and Chou,P. | Does supplementation with green tea extract improve insulin resistance in obese type 2 diabetics? A randomized, double-blind, and placebo-controlled clinical trial | Altern Med Rev. 2011 Jun;16(2):157-63. | 21649457 |
| 94 | Hsu,C.H. and Tsai,T.H. and Kao,Y.H. and Hwang,K.C. and Tseng,T.Y. and Chou,P. | Effect of green tea extract on obese women: a randomized, double-blind, placebo-controlled clinical trial | Clin Nutr. 2008 Jun;27(3):363-70. | 18468736 |
| 95 | Huang,P.H. and Chen,Y.H. and Tsai,H.Y. and Chen,J.S. and Wu,T.C. and Lin,F.Y. and Sata,M. and Chen,J.W. and Lin,S.J. | Intake of red wine increases the number and functional capacity of circulating endothelial progenitor cells by enhancing nitric oxide bioavailability | Arterioscler Thromb Vasc Biol. 2010 Apr;30(4):869-77. | 20093623 |
| 96 | Ibero-Baraibar,I. and Abete,I. and Navas-Carretero,S. and Massis-Zaid,A. and Martinez,J.A. and Zulet,M.A. | Oxidised LDL levels decreases after the consumption of ready-to-eat meals supplemented with cocoa extract within a hypocaloric diet | Nutr Metab Cardiovasc Dis. 2014 Apr;24(4):416-22. | 24462367 |
| 97 | Inami,S. and Takano,M. and Yamamoto,M. and Murakami,D. and Tajika,K. and Yodogawa,K. and Yokoyama,S. and Ohno,N. and Ohba,T. and Sano,J. and Ibuki,C. and Seino,Y. and Mizuno,K. | Tea catechin consumption reduces circulating oxidized low-density lipoprotein | Int Heart J. 2007 Nov;48(6):725-32. | 18160764 |
| 98 | Jenkins,D.J. and Nguyen,T.H. and Kendall,C.W. and Faulkner,D.A. and Bashyam,B. and Kim,I.J. and Ireland,C. and Patel,D. and Vidgen,E. and Josse,A.R. and Sesso,H.D. and Burton-Freeman,B. and Josse,R.G. and Leiter,L.A. and Singer,W. | The effect of strawberries in a cholesterol-lowering dietary portfolio | Metabolism. 2008 Dec;57(12):1636-44. | 19013285 |
| 99 | Jensen,G.S. and Beaman,J.L. and He,Y. and Guo,Z. and Sun,H. | Reduction of body fat and improved lipid profile associated with daily consumption of a Puer tea extract in a hyperlipidemic population: a randomized placebo-controlled trial | Clin Interv Aging. 2016 Mar 24;11:367-76. | 27069360 |
| 100 | Jeong,H.S. and Hong,S.J. and Cho,J.Y. and Lee,T.B. and Kwon,J.W. and Joo,H.J. and Park,J.H. and Yu,C.W. and Lim,D.S. | Effects of Rubus occidentalis extract on blood pressure in patients with prehypertension: Randomized, double-blinded, placebo-controlled clinical trial | Nutrition. 2016 Apr;32(4):461-7. | 26740254 |
| 101 | Jeong,H.S. and Hong,S.J. and Lee,T.B. and Kwon,J.W. and Jeong,J.T. and Joo,H.J. and Park,J.H. and Ahn,C.M. and Yu,C.W. and Lim,D.S. | Effects of black raspberry on lipid profiles and vascular endothelial function in patients with metabolic syndrome | Phytother Res. 2014 Oct;28(10):1492-8. | 24706588 |
| 102 | Jeong,H.S. and Kim,S. and Hong,S.J. and Choi,S.C. and Choi,J.H. and Kim,J.H. and Park,C.Y. and Cho,J.Y. and Lee,T.B. and Kwon,J.W. and Joo,H.J. and Park,J.H. and Yu,C.W. and Lim,D.S. | Black Raspberry Extract Increased Circulating Endothelial Progenitor Cells and Improved Arterial Stiffness in Patients with Metabolic Syndrome: A Randomized Controlled Trial | J Med Food. 2016 Apr;19(4):346-52. | 26891216 |
| 103 | Jimenez,J.P. and Serrano,J. and Tabernero,M. and Arranz,S. and Diaz-Rubio,M.E. and Garcia-Diz,L. and Goni,I. and Saura-Calixto,F. | Effects of grape antioxidant dietary fiber in cardiovascular disease risk factors | Nutrition. 2008 Jul-Aug;24(7-8):646-53. | 18485668 |
| 104 | Jochmann,N. and Lorenz,M. and Krosigk,Av and Martus,P. and Bohm,V. and Baumann,G. and Stangl,K. and Stangl,V. | The efficacy of black tea in ameliorating endothelial function is equivalent to that of green tea | Br J Nutr. 2008 Apr;99(4):863-8. Epub 2007 Oct 5. | 17916273 |
| 105 | Johnson,S.A. and Figueroa,A. and Navaei,N. and Wong,A. and Kalfon,R. and Ormsbee,L.T. and Feresin,R.G. and Elam,M.L. and Hooshmand,S. and Payton,M.E. and Arjmandi,B.H. | Daily blueberry consumption improves blood pressure and arterial stiffness in postmenopausal women with pre- and stage 1-hypertension: a randomized, double-blind, placebo-controlled clinical trial | J Acad Nutr Diet. 2015 Mar;115(3):369-77. | 25578927 |
| 106 | Kar,P. and Laight,D. and Rooprai,H.K. and Shaw,K.M. and Cummings,M. | Effects of grape seed extract in Type 2 diabetic subjects at high cardiovascular risk: a double blind randomized placebo controlled trial examining metabolic markers, vascular tone, inflammation, oxidative stress and insulin sensitivity | Diabet Med. 2009 May;26(5):526-31. | 19646193 |
| 107 | Karlsen,A. and Paur,I. and Bohn,S.K. and Sakhi,A.K. and Borge,G.I. and Serafini,M. and Erlund,I. and Laake,P. and Tonstad,S. and Blomhoff,R. | Bilberry juice modulates plasma concentration of NF-kappaB related inflammatory markers in subjects at increased risk of CVD | Eur J Nutr. 2010 Sep;49(6):345-55. | 20119859 |
| 108 | Kaul,S. and Belcik,T. and Kalvaitis,S. and Jayaweera,A.R. and Choi,S.W. and Wei,K. | Effect of modest alcohol consumption over 1-2 weeks on the coronary microcirculation of normal subjects | Eur J Echocardiogr. 2010 Sep;11(8):683-9. | 20378684 |
| 109 | Kechagias,S. and Zanjani,S. and Gjellan,S. and Leinhard,O.D. and Kihlberg,J. and Smedby,O. and Johansson,L. and Kullberg,J. and Ahlstrom,H. and Lindstrom,T. and Nystrom,F.H. | Effects of moderate red wine consumption on liver fat and blood lipids: a prospective randomized study | Ann Med. 2011 Nov;43(7):545-54. | 21599573 |
| 110 | Khan A, Khattak KN, Safdar M, Anderson RA, Ali Khan MM | Cinnamon improves glucose and lipids of people with type 2 diabetes | Diabetes Care. 2003 Dec;26(12):3215-8. | 14633804 |
| 111 | Khan,N. and Monagas,M. and Andres-Lacueva,C. and Casas,R. and Urpi-Sarda,M. and Lamuela-Raventos,R.M. and Estruch,R. | Regular consumption of cocoa powder with milk increases HDL cholesterol and reduces oxidized LDL levels in subjects at high-risk of cardiovascular disease | Nutr Metab Cardiovasc Dis. 2012 Dec;22(12):1046-53. | 21550218 |
| 112 | Klein,G.A. and Stefanuto,A. and Boaventura,B.C. and de Morais,E.C. and Cavalcante,Lda S. and de,Andrade F. and Wazlawik,E. and Di Pietro,P.F. and Maraschin,M. and da Silva,E.L. | Mate tea (Ilex paraguariensis) improves glycemic and lipid profiles of type 2 diabetes and pre-diabetes individuals: a pilot study | J Am Coll Nutr. 2011 Oct;30(5):320-32. | 22081618 |
| 113 | Kolehmainen,M. and Mykkanen,O. and Kirjavainen,P.V. and Leppanen,T. and Moilanen,E. and Adriaens,M. and Laaksonen,D.E. and Hallikainen,M. and Puupponen-Pimia,R. and Pulkkinen,L. and Mykkanen,H. and Gylling,H. and Poutanen,K. and Torronen,R. | Bilberries reduce low-grade inflammation in individuals with features of metabolic syndrome | Mol Nutr Food Res. 2012 Oct;56(10):1501-10. | 22961907 |
| 114 | Koli,R. and Kohler,K. and Tonteri,E. and Peltonen,J. and Tikkanen,H. and Fogelholm,M. | Dark chocolate and reduced snack consumption in mildly hypertensive adults: an intervention study | Nutr J. 2015 Aug 22;14:84. | 26296850 |
| 115 | Lasaite L and Spadiene A and Savickiene N and Skesters A and Silova A | The effect of Ginkgo biloba and Camellia sinensis extracts on psychological state and glycemic control in patients with type 2 diabetes mellitus. | Nat Prod Commun. 2014 Sep;9(9):1345-50. | 25918808 |
| 116 | Lavy,A. and Fuhrman,B. and Markel,A. and Dankner,G. and Ben-Amotz,A. and Presser,D. and Aviram,M. | Effect of dietary supplementation of red or white wine on human blood chemistry, hematology and coagulation: favorable effect of red wine on plasma high-density lipoprotein | Ann Nutr Metab. 1994;38(5):287-94. | 7535990 |
| 117 | Lee,I.T. and Chan,Y.C. and Lin,C.W. and Lee,W.J. and Sheu,W.H. | Effect of cranberry extracts on lipid profiles in subjects with Type 2 diabetes | Diabet Med. 2008 Dec;25(12):1473-7. | 19046248 |
| 118 | Lehtonen,H.M. and Suomela,J.P. and Tahvonen,R. and Yang,B. and Venojarvi,M. and Viikari,J. and Kallio,H. | Different berries and berry fractions have various but slightly positive effects on the associated variables of metabolic diseases on overweight and obese women | Eur J Clin Nutr. 2011 Mar;65(3):394-401. | 21224867 |
| 119 | Liu,C.Y. and Huang,C.J. and Huang,L.H. and Chen,I.J. and Chiu,J.P. and Hsu,C.H. | Effects of green tea extract on insulin resistance and glucagon-like peptide 1 in patients with type 2 diabetes and lipid abnormalities: a randomized, double-blinded, and placebo-controlled trial | PLoS One. 2014 Mar 10;9(3):e91163. | 24614112 |
| 120 | Loffredo,L. and Perri,L. and Catasca,E. and Pignatelli,P. and Brancorsini,M. and Nocella,C. and De,Falco E. and Bartimoccia,S. and Frati,G. and Carnevale,R. and Violi,F. | Dark chocolate acutely improves walking autonomy in patients with peripheral artery disease | J Am Heart Assoc. 2014 Jul 2;3(4). pii: e001072. | 24990275 |
| 121 | Loktionov,A. and Bingham,S.A. and Vorster,H. and Jerling,J.C. and Runswick,S.A. and Cummings,J.H. | Apolipoprotein E genotype modulates the effect of black tea drinking on blood lipids and blood coagulation factors: a pilot study | Br J Nutr. 1998 Feb;79(2):133-9. | 9536857 |
| 122 | Lu,T. and Sheng,H. and Wu,J. and Cheng,Y. and Zhu,J. and Chen,Y. | Cinnamon extract improves fasting blood glucose and glycosylated hemoglobin level in Chinese patients with type 2 diabetes | Nutr Res. 2012 Jun;32(6):408-12. | 22749176 |
| 123 | Lynn,A. and Mathew,S. and Moore,C.T. and Russell,J. and Robinson,E. and Soumpasi,V. and Barker,M.E. | Effect of a tart cherry juice supplement on arterial stiffness and inflammation in healthy adults: a randomised controlled trial | Plant Foods Hum Nutr. 2014 Jun;69(2):122-7. | 24570273 |
| 124 | MacKenzie,T. and Leary,L. and Brooks,W.B. | The effect of an extract of green and black tea on glucose control in adults with type 2 diabetes mellitus: double-blind randomized study | Metabolism. 2007 Oct;56(10):1340-4. | 17884442 |
| 125 | Mahmoud,F. and Haines,D. and Al-Ozairi,E. and Dashti,A. | Effect of Black Tea Consumption on Intracellular Cytokines, Regulatory T Cells and Metabolic Biomarkers in Type 2 Diabetes Patients | Phytother Res. 2016 Mar;30(3):454-62. | 26692322 |
| 126 | Mang B, Wolters M, Schmitt B, et al. | Effects of a cinnamon extract on plasma glucose, HbA1c, and serum lipids in diabetes mellitus type 2. | Eur J Clin Invest. 2006 May;36(5):340-4. | 16634838 |
| 127 | Martinez-Lopez,S. and Sarria,B. and Sierra-Cinos,J.L. and Goya,L. and Mateos,R. and Bravo,L. | Realistic intake of a flavanol-rich soluble cocoa product increases HDL-cholesterol without inducing anthropometric changes in healthy and moderately hypercholesterolemic subjects | Food Funct. 2014 Feb;5(2):364-74. | 24394704 |
| 128 | Massee,L.A. and Ried,K. and Pase,M. and Travica,N. and Yoganathan,J. and Scholey,A. and Macpherson,H. and Kennedy,G. and Sali,A. and Pipingas,A. | The acute and sub-chronic effects of cocoa flavanols on mood, cognitive and cardiovascular health in young healthy adults: a randomized, controlled trial | Front Pharmacol. 2015 May 20;6:93. | 26042037 |
| 129 | Mastroiacovo,D. and Kwik-Uribe,C. and Grassi,D. and Necozione,S. and Raffaele,A. and Pistacchio,L. and Righetti,R. and Bocale,R. and Lechiara,M.C. and Marini,C. and Ferri,C. and Desideri,G. | Cocoa flavanol consumption improves cognitive function, blood pressure control, and metabolic profile in elderly subjects: the Cocoa, Cognition, and Aging (CoCoA) Study--a randomized controlled trial | Am J Clin Nutr. 2015 Mar;101(3):538-48. | 25733639 |
| 130 | McAnulty,L.S. and Collier,S.R. and Landram,M.J. and Whittaker,D.S. and Isaacs,S.E. and Klemka,J.M. and Cheek,S.L. and Arms,J.C. and McAnulty,S.R. | Six weeks daily ingestion of whole blueberry powder increases natural killer cell counts and reduces arterial stiffness in sedentary males and females | Nutr Res. 2014 Jul;34(7):577-84. | 25150116 |
| 131 | McFarlin,B.K. and Venable,A.S. and Henning,A.L. and Prado,E.A. and Best Sampson,J.N. and Vingren,J.L. and Hill,D.W. | Natural cocoa consumption: Potential to reduce atherogenic factors? | J Nutr Biochem. 2015 Jun;26(6):626-32. | 25769436 |
| 132 | Mellen,P.B. and Daniel,K.R. and Brosnihan,K.B. and Hansen,K.J. and Herrington,D.M. | Effect of muscadine grape seed supplementation on vascular function in subjects with or at risk for cardiovascular disease: a randomized crossover trial | J Am Coll Nutr. 2010 Oct;29(5):469-75. | 21504973 |
| 133 | Mellor,D.D. and Sathyapalan,T. and Kilpatrick,E.S. and Beckett,S. and Atkin,S.L. | High-cocoa polyphenol-rich chocolate improves HDL cholesterol in Type 2 diabetes patients | Diabet Med. 2010 Nov;27(11):1318-21. | 20968113 |
| 134 | Mielgo-Ayuso,J. and Barrenechea,L. and Alcorta,P. and Larrarte,E. and Margareto,J. and Labayen,I. | Effects of dietary supplementation with epigallocatechin-3-gallate on weight loss, energy homeostasis, cardiometabolic risk factors and liver function in obese women: randomised, double-blind, placebo-controlled clinical trial | Br J Nutr. 2014 Apr 14;111(7):1263-71. | 24299662 |
| 135 | Mirfeizi,M. and Mirfeizi,S. and MehdizadehTourzani,Z. and AsghariJafarabadi,M. | Controlling diabetes mellitus type 2 with herbal medicines: A triple blind, randomized clinical trial of efficacy and safety | J Diabetes. 2016 Sep;8(5):647-56. | 26362826 |
| 136 | Mirzaei,K. and Hossein-Nezhad,A. and Karimi,M. and Hosseinzadeh-Attar,M.J. and Jafari,N. and Najmafshar,A. and Larijani,B. | Effect of green tea extract on bone turnover markers in type 2 diabetic patients; A double- Blind, placebo-controlled clinical trial study | DARU. 2009; 17 (Suppl. 1) | None |
| 137 | Miyazaki,R. and Kotani,K. and Ayabe,M. and Tsuzaki,K. and Shimada,J. and Sakane,N. and Takase,H. and Ichikawa,H. and Yonei,Y. and Ishii,K. | Minor effects of green tea catechin supplementation on cardiovascular risk markers in active older people: a randomized controlled trial | Geriatr Gerontol Int. 2013 Jul;13(3):622-9. | 23035743 |
| 138 | Moazen,S. and Amani,R. and Homayouni,Rad A. and Shahbazian,H. and Ahmadi,K. and Taha,Jalali M. | Effects of freeze-dried strawberry supplementation on metabolic biomarkers of atherosclerosis in subjects with type 2 diabetes: a randomized double-blind controlled trial | Ann Nutr Metab. 2013;63(3):256-64. | 24334868 |
| 139 | Monagas,M. and Khan,N. and Andres-Lacueva,C. and Casas,R. and Urpi-Sarda,M. and Llorach,R. and Lamuela-Raventos,R.M. and Estruch,R. | Effect of cocoa powder on the modulation of inflammatory biomarkers in patients at high risk of cardiovascular disease | Am J Clin Nutr. 2009 Nov;90(5):1144-50. | 19776136 |
| 140 | Monahan,K.D. and Feehan,R.P. and Kunselman,A.R. and Preston,A.G. and Miller,D.L. and Lott,M.E. | Dose-dependent increases in flow-mediated dilation following acute cocoa ingestion in healthy older adults | J Appl Physiol (1985). 2011 Dec;111(6):1568-74. | 21903881 |
| 141 | Mori,T.A. and Burke,V. and Beilin,L.J. and Puddey,I.B. | Randomized Controlled Intervention of the Effects of Alcohol on Blood Pressure in Premenopausal Women | Hypertension. 2015 Sep;66(3):517-23. | 26123682 |
| 142 | Mori,T.A. and Burke,V. and Zilkens,R.R. and Hodgson,J.M. and Beilin,L.J. and Puddey,I.B. | The effects of alcohol on ambulatory blood pressure and other cardiovascular risk factors in type 2 diabetes: a randomized intervention | J Hypertens. 2016 Mar;34(3):421-8; discussion 428. | 26734954 |
| 143 | Mousavi,A. and Vafa,M. and Neyestani,T. and Khamseh,M. and Hoseini,F. | The effects of green tea consumption on metabolic and anthropometric indices in patients with Type 2 diabetes | J Res Med Sci. 2013 Dec;18(12):1080-6. | 24523800 |
| 144 | Mozaffari-Khosravi H and Ahadi Z and Barzegar K | The effect of green tea and sour tea on blood pressure of patients with type 2 diabetes: a randomized clinical trial. | J Diet Suppl. 2013 Jun;10(2):105-15. | 23725524 |
| 145 | Mozaffari-Khosravi,H. and Jalali-Khanabadi,B.A. and Afkhami-Ardekani,M. and Fatehi,F. | Effects of sour tea (Hibiscus sabdariffa) on lipid profile and lipoproteins in patients with type II diabetes | J Altern Complement Med. 2009 Aug;15(8):899-903. | 19678781 |
| 146 | Mukamal,K.J. and MacDermott,K. and Vinson,J.A. and Oyama,N. and Manning,W.J. and Mittleman,M.A. | A 6-month randomized pilot study of black tea and cardiovascular risk factors | Am Heart J. 2007 Oct;154(4):724.e1-6. | 17892999 |
| 147 | Munguia,L. and Gutierrez-Salmean,G. and Hernandez,M. and Ortiz,A. and Sanchez,M.E. and Najera,N. and Meaney,E. and Rubio-Gayosso,I. and Ceballos,G. | Beneficial effects of a flavanol-enriched cacao beverage on anthropometric and cardiometabolic risk profile in overweight subjects | Rev. Mex. Cardiol. 2015, 26 (2):78-86. | None |
| 148 | Muniyappa,R. and Hall,G. and Kolodziej,T.L. and Karne,R.J. and Crandon,S.K. and Quon,M.J. | Cocoa consumption for 2 wk enhances insulin-mediated vasodilatation without improving blood pressure or insulin resistance in essential hypertension | Am J Clin Nutr. 2008 Dec;88(6):1685-96. | 19064532 |
| 149 | Nagao,T. and Hase,T. and Tokimitsu,I. | A green tea extract high in catechins reduces body fat and cardiovascular risks in humans | Obesity (Silver Spring). 2007 Jun;15(6):1473-83. | 17557985 |
| 150 | Nagao,T. and Meguro,S. and Hase,T. and Otsuka,K. and Komikado,M. and Tokimitsu,I. and Yamamoto,T. and Yamamoto,K. | A catechin-rich beverage improves obesity and blood glucose control in patients with type 2 diabetes | Obesity (Silver Spring). 2009 Feb;17(2):310-7. | 19008868 |
| 151 | Naruszewicz,M. and Laniewska,I. and Millo,B. and Dluzniewski,M. | Combination therapy of statin with flavonoids rich extract from chokeberry fruits enhanced reduction in cardiovascular risk markers in patients after myocardial infraction (MI) | Atherosclerosis. 2007 Oct;194(2):e179-84. Epub 2007 Feb 21. | 17320090 |
| 152 | Neufingerl,N. and Zebregs,Y.E. and Schuring,E.A. and Trautwein,E.A. | Effect of cocoa and theobromine consumption on serum HDL-cholesterol concentrations: a randomized controlled trial | Am J Clin Nutr. 2013 Jun;97(6):1201-9. | 23595874 |
| 153 | Neyestani,T.R. and Shariatzade,N. and Kalayi,A. and Gharavi,A. and Khalaji,N. and Dadkhah,M. and Zowghi,T. and Haidari,H. and Shab-bidar,S. | Regular daily intake of black tea improves oxidative stress biomarkers and decreases serum C-reactive protein levels in type 2 diabetic patients | Ann Nutr Metab. 2010;57(1):40-9. | 20668372 |
| 154 | Nickols-Richardson,S.M. and Piehowski,K.E. and Metzgar,C.J. and Miller,D.L. and Preston,A.G. | Changes in body weight, blood pressure and selected metabolic biomarkers with an energy-restricted diet including twice daily sweet snacks and once daily sugar-free beverage | Nutr Res Pract. 2014 Dec;8(6):695-704. | 25489410 |
| 155 | Njike,V.Y. and Faridi,Z. and Shuval,K. and Dutta,S. and Kay,C.D. and West,S.G. and Kris-Etherton,P.M. and Katz,D.L. | Effects of sugar-sweetened and sugar-free cocoa on endothelial function in overweight adults | Int J Cardiol. 2011 May 19;149(1):83-8. | 20036019 |
| 156 | Novotny,J.A. and Baer,D.J. and Khoo,C. and Gebauer,S.K. and Charron,C.S. | Cranberry juice consumption lowers markers of cardiometabolic risk, including blood pressure and circulating C-reactive protein, triglyceride, and glucose concentrations in adults | J Nutr. 2015 Jun;145(6):1185-93. | 25904733 |
| 157 | Ottaviani,J.I. and Balz,M. and Kimball,J. and Ensunsa,J.L. and Fong,R. and Momma,T.Y. and Kwik-Uribe,C. and Schroeter,H. and Keen,C.L. | Safety and efficacy of cocoa flavanol intake in healthy adults: a randomized, controlled, double-masked trial | Am J Clin Nutr. 2015 Dec;102(6):1425-35. | 26537937 |
| 158 | Park,E. and Edirisinghe,I. and Choy,Y.Y. and Waterhouse,A. and Burton-Freeman,B. | Effects of grape seed extract beverage on blood pressure and metabolic indices in individuals with pre-hypertension: a randomised, double-blinded, two-arm, parallel, placebo-controlled trial | Br J Nutr. 2016 Jan 28;115(2):226-38. | 26568249 |
| 159 | Park,Y.K. and Lee,S.H. and Park,E. and Kim,J.S. and Kang,M.H. | Changes in antioxidant status, blood pressure, and lymphocyte DNA damage from grape juice supplementation | Ann N Y Acad Sci. 2009 Aug;1171:385-90. | 19723080 |
| 160 | Parsaeyan,N. and Mozaffari-Khosravi,H. and Absalan,A. and Mozayan,M.R. | Beneficial effects of cocoa on lipid peroxidation and inflammatory markers in type 2 diabetic patients and investigation of probable interactions of cocoa active ingredients with prostaglandin synthase-2 (PTGS-2/COX-2) using virtual analysis | J Diabetes Metab Disord. 2014 Feb 4;13(1):30. | 24495354 |
| 161 | Pereira,T. and Maldonado,J. and Laranjeiro,M. and Coutinho,R. and Cardoso,E. and Andrade,I. and Conde,J. | Central arterial hemodynamic effects of dark chocolate ingestion in young healthy people: a randomized and controlled trial | Cardiol Res Pract. 2014;2014:945951. | 24982813 |
| 162 | Puupponen-Pimia,R. and Seppanen-Laakso,T. and Kankainen,M. and Maukonen,J. and Torronen,R. and Kolehmainen,M. and Leppanen,T. and Moilanen,E. and Nohynek,L. and Aura,A.M. and Poutanen,K. and Tomas-Barberan,F.A. and Espin,J.C. and Oksman-Caldentey,K.M. | Effects of ellagitannin-rich berries on blood lipids, gut microbiota, and urolithin production in human subjects with symptoms of metabolic syndrome | Mol Nutr Food Res. 2013 Dec;57(12):2258-63. | 23934737 |
| 163 | Queipo-Ortuno MI and Boto-Ordonez M and Murri M and Gomez-Zumaquero JM and Clemente-Postigo M and Estruch R and Cardona Diaz F and Andres-Lacueva C and Tinahones FJ | Influence of red wine polyphenols and ethanol on the gut microbiota ecology and biochemical biomarkers. | Am J Clin Nutr. 2012 Jun;95(6):1323-34. | 22552027 |
| 164 | Rahbar,A.R. and Mahmoudabadi,M.M. and Islam,M.S. | Comparative effects of red and white grapes on oxidative markers and lipidemic parameters in adult hypercholesterolemic humans | Food Funct. 2015 Jun;6(6):1992-8. | 26007320 |
| 165 | Ras,R.T. and Zock,P.L. and Zebregs,Y.E. and Johnston,N.R. and Webb,D.J. and Draijer,R. | Effect of polyphenol-rich grape seed extract on ambulatory blood pressure in subjects with pre- and stage I hypertension | Br J Nutr. 2013 Dec;110(12):2234-41. | 23702253 |
| 166 | Ravn-Haren,G. and Dragsted,L.O. and Buch-Andersen,T. and Jensen,E.N. and Jensen,R.I. and Nemeth-Balogh,M. and Paulovicsova,B. and Bergstrom,A. and Wilcks,A. and Licht,T.R. and Markowski,J. and Bugel,S. | Intake of whole apples or clear apple juice has contrasting effects on plasma lipids in healthy volunteers | Eur J Nutr. 2013 Dec;52(8):1875-89. | 23271615 |
| 167 | Razavi,S.M. and Gholamin,S. and Eskandari,A. and Mohsenian,N. and Ghorbanihaghjo,A. and Delazar,A. and Rashtchizadeh,N. and Keshtkar-Jahromi,M. and Argani,H. | Red grape seed extract improves lipid profiles and decreases oxidized low-density lipoprotein in patients with mild hyperlipidemia | J Med Food. 2013 Mar;16(3):255-8. | 23437789 |
| 168 | Ried,K. and Frank,O.R. and Stocks,N.P. | Dark chocolate or tomato extract for prehypertension: a randomised controlled trial | BMC Complement Altern Med. 2009 Jul 8;9:22. | 19583878 |
| 169 | Rifler,J.P. and Lorcerie,F. and Durand,P. and Delmas,D. and Ragot,K. and Limagne,E. and Mazue,F. and Riedinger,J.M. and d'Athis,P. and Hudelot,B. and Prost,M. and Lizard,G. and Latruffe,N. | A moderate red wine intake improves blood lipid parameters and erythrocytes membrane fluidity in post myocardial infarct patients | Mol Nutr Food Res. 2012 Feb;56(2):345-51. | 22419533 |
| 170 | Riso,P. and Klimis-Zacas,D. and Del,Bo' C. and Martini,D. and Campolo,J. and Vendrame,S. and Moller,P. and Loft,S. and De,Maria R. and Porrini,M. | Effect of a wild blueberry (Vaccinium angustifolium) drink intervention on markers of oxidative stress, inflammation and endothelial function in humans with cardiovascular risk factors | Eur J Nutr. 2013 Apr;52(3):949-61. | 22733001 |
| 171 | Rodriguez-Mateos A and Del Pino-Garcia R and George TW and Vidal-Diez A and Heiss C and Spencer JP | Impact of processing on the bioavailability and vascular effects of blueberry (poly)phenols. | Mol Nutr Food Res. 2014 Oct;58(10):1952-61. | 25044909 |
| 172 | Rodriguez-Mateos,A. and Rendeiro,C. and Bergillos-Meca,T. and Tabatabaee,S. and George,T.W. and Heiss,C. and Spencer,J.P. | Intake and time dependence of blueberry flavonoid-induced improvements in vascular function: a randomized, controlled, double-blind, crossover intervention study with mechanistic insights into biological activity | Am J Clin Nutr. 2013 Nov;98(5):1179-91. | 24004888 |
| 173 | Rostami,A. and Khalili,M. and Haghighat,N. and Eghtesadi,S. and Shidfar,F. and Heidari,I. and Ebrahimpour-Koujan,S. and Eghtesadi,M. | High-cocoa polyphenol-rich chocolate improves blood pressure in patients with diabetes and hypertension | ARYA Atheroscler. 2015 Jan;11(1):21-9. | 26089927 |
| 174 | Roussel,A.M. and Hininger,I. and Benaraba,R. and Ziegenfuss,T.N. and Anderson,R.A. | Antioxidant effects of a cinnamon extract in people with impaired fasting glucose that are overweight or obese | J Am Coll Nutr. 2009 Feb;28(1):16-21. | 19571155 |
| 175 | Ruel,G. and Lapointe,A. and Pomerleau,S. and Couture,P. and Lemieux,S. and Lamarche,B. and Couillard,C. | Evidence that cranberry juice may improve augmentation index in overweight men | Nutr Res. 2013 Jan;33(1):41-9. | 23351409 |
| 176 | Sahib,A.S. | Anti-diabetic and antioxidant effect of cinnamon in poorly controlled type-2 diabetic Iraqi patients: A randomized, placebo-controlled clinical trial | J Intercult Ethnopharmacol. 2016 Feb 21;5(2):108-13 | 27104030 |
| 177 | Sano,A. and Uchida,R. and Saito,M. and Shioya,N. and Komori,Y. and Tho,Y. and Hashizume,N. | Beneficial effects of grape seed extract on malondialdehyde-modified LDL | J Nutr Sci Vitaminol (Tokyo). 2007 Apr;53(2):174-82. | 17616006 |
| 178 | Sansone,R. and Rodriguez-Mateos,A. and Heuel,J. and Falk,D. and Schuler,D. and Wagstaff,R. and Kuhnle,G.G. and Spencer,J.P. and Schroeter,H. and Merx,M.W. and Kelm,M. and Heiss,C. and FLAVIOLA Consortium, | Cocoa flavanol intake improves endothelial function and Framingham Risk Score in healthy men and women: a randomised, controlled, double-masked trial: the Flaviola Health Study | Br J Nutr. 2015 Oct 28;114(8):1246-55. | 26348767 |
| 179 | Schreuder,T.H. and Eijsvogels,T.M. and Greyling,A. and Draijer,R. and Hopman,M.T. and Thijssen,D.H. | Effect of black tea consumption on brachial artery flow-mediated dilation and ischaemia-reperfusion in humans | Appl Physiol Nutr Metab. 2014 Feb;39(2):145-51. | 24476469 |
| 180 | Shidfar,F. and Heydari,I. and Hajimiresmaiel,S.J. and Hosseini,S. and Shidfar,S. and Amiri,F. | The effects of cranberry juice on serum glucose, apoB, apoA-I, Lp(a), and Paraoxonase-1 activity in type 2 diabetic male patients | J Res Med Sci. 2012 Apr;17(4):355-60. | 23267397 |
| 181 | Simao,T.N. and Lozovoy,M.A. and Simao,A.N. and Oliveira,S.R. and Venturini,D. and Morimoto,H.K. and Miglioranza,L.H. and Dichi,I. | Reduced-energy cranberry juice increases folic acid and adiponectin and reduces homocysteine and oxidative stress in patients with the metabolic syndrome | Br J Nutr. 2013 Nov;110(10):1885-94. | 23750500 |
| 182 | Sivaprakasapillai,B. and Edirisinghe,I. and Randolph,J. and Steinberg,F. and Kappagoda,T. | Effect of grape seed extract on blood pressure in subjects with the metabolic syndrome | Metabolism. 2009 Dec;58(12):1743-6. | 19608210 |
| 183 | Sone,T. and Kuriyama,S. and Nakaya,N. and Hozawa,A. and Shimazu,T. and Nomura,K. and Rikimaru,S. and Tsuji,I. | Randomized controlled trial for an effect of catechin-enriched green tea consumption on adiponectin and cardiovascular disease risk factors | Food Nutr Res. 2011;55. | 22144918 |
| 184 | Sorond,F.A. and Lipsitz,L.A. and Hollenberg,N.K. and Fisher,N.D. | Cerebral blood flow response to flavanol-rich cocoa in healthy elderly humans | Neuropsychiatr Dis Treat. 2008 Apr;4(2):433-40. | 18728792 |
| 185 | Stendell-Hollis NR and Thomson CA and Thompson PA and Bea JW and Cussler EC and Hakim IA | Green tea improves metabolic biomarkers, not weight or body composition: a pilot study in overweight breast cancer survivors. | J Hum Nutr Diet. 2010 Dec;23(6):590-600. | 20807303 |
| 186 | Stull,A.J. and Cash,K.C. and Champagne,C.M. and Gupta,A.K. and Boston,R. and Beyl,R.A. and Johnson,W.D. and Cefalu,W.T. | Blueberries improve endothelial function, but not blood pressure, in adults with metabolic syndrome: a randomized, double-blind, placebo-controlled clinical trial | Nutrients. 2015 May 27;7(6):4107-23. | 26024297 |
| 187 | Stull,A.J. and Cash,K.C. and Johnson,W.D. and Champagne,C.M. and Cefalu,W.T. | Bioactives in blueberries improve insulin sensitivity in obese, insulin-resistant men and women | J Nutr. 2010 Oct;140(10):1764-8. | 20724487 |
| 188 | Suliburska,J. and Bogdanski,P. and Szulinska,M. and Stepien,M. and Pupek-Musialik,D. and Jablecka,A. | Effects of green tea supplementation on elements, total antioxidants, lipids, and glucose values in the serum of obese patients | Biol Trace Elem Res. 2012 Dec;149(3):315-22. | 22581111 |
| 189 | Takahashi,M. and Miyashita,M. and Suzuki,K. and Bae,S.R. and Kim,H.K. and Wakisaka,T. and Matsui,Y. and Takeshita,M. and Yasunaga,K. | Acute ingestion of catechin-rich green tea improves postprandial glucose status and increases serum thioredoxin concentrations in postmenopausal women | Br J Nutr. 2014 Nov 14;112(9):1542-50. | 25230741 |
| 190 | Tang,M. and Larson-Meyer,D.E. and Liebman,M. | Effect of cinnamon and turmeric on urinary oxalate excretion, plasma lipids, and plasma glucose in healthy subjects | Am J Clin Nutr. 2008 May;87(5):1262-7. | 18469248 |
| 191 | Tangvarasittichai,S. and Sanguanwong,S. and Sengsuk,C. and Tangvarasittichai,O. | Effect of cinnamon supplementation on oxidative stress, inflammation and insulin resistance in patients with type 2 diabetes mellitus | IJTPR. 2015, 7(4): 1-7. | None |
| 192 | Taubert,D. and Roesen,R. and Lehmann,C. and Jung,N. and Schomig,E. | Effects of low habitual cocoa intake on blood pressure and bioactive nitric oxide: a randomized controlled trial | JAMA. 2007 Jul 4;298(1):49-60. | 17609490 |
| 193 | Terauchi,M. and Horiguchi,N. and Kajiyama,A. and Akiyoshi,M. and Owa,Y. and Kato,K. and Kubota,T. | Effects of grape seed proanthocyanidin extract on menopausal symptoms, body composition, and cardiovascular parameters in middle-aged women: a randomized, double-blind, placebo-controlled pilot study | Menopause. 2014 Sep;21(9):990-6. | 24518152 |
| 194 | Tinahones,F.J. and Rubio,M.A. and Garrido-Sanchez,L. and Ruiz,C. and Gordillo,E. and Cabrerizo,L. and Cardona,F. | Green tea reduces LDL oxidability and improves vascular function | J Am Coll Nutr. 2008 Apr;27(2):209-13. | 18689551 |
| 195 | Tome-Carneiro,J. and Gonzalvez,M. and Larrosa,M. and Garcia-Almagro,F.J. and Aviles-Plaza,F. and Parra,S. and Yanez-Gascon,M.J. and Ruiz-Ros,J.A. and Garcia-Conesa,M.T. and Tomas-Barberan,F.A. and Espin,J.C. | Consumption of a grape extract supplement containing resveratrol decreases oxidized LDL and ApoB in patients undergoing primary prevention of cardiovascular disease: a triple-blind, 6-month follow-up, placebo-controlled, randomized trial | Mol Nutr Food Res. 2012 May;56(5):810-21. | 22648627 |
| 196 | Toolsee,N.A. and Aruoma,O.I. and Gunness,T.K. and Kowlessur,S. and Dambala,V. and Murad,F. and Googoolye,K. and Daus,D. and Indelicato,J. and Rondeau,P. and Bourdon,E. and Bahorun,T. | Effectiveness of green tea in a randomized human cohort: relevance to diabetes and its complications | Biomed Res Int. 2013;2013:412379. | 24102055 |
| 197 | Torres,A. and Cachofeiro,V. and Millan,J. and Lahera,V. and Nieto,M.L. and Martin,R. and Bello,E. and Alvarez-Sala,L.A. | Red wine intake but not other alcoholic beverages increases total antioxidant capacity and improves pro-inflammatory profile after an oral fat diet in healthy volunteers | Rev Clin Esp. 2015 Dec;215(9):486-94. | 26297333 |
| 198 | Trautwein,E.A. and Du,Y. and Meynen,E. and Yan,X. and Wen,Y. and Wang,H. and Molhuizen,H.O. | Purified black tea theaflavins and theaflavins/catechin supplements did not affect serum lipids in healthy individuals with mildly to moderately elevated cholesterol concentrations | Eur J Nutr. 2010 Feb;49(1):27-35. | 19639377 |
| 199 | Troup,R. and Hayes,J.H. and Raatz,S.K. and Thyagarajan,B. and Khaliq,W. and Jacobs,D.R.,Jr. and Key,N.S. and Morawski,B.M. and Kaiser,D. and Bank,A.J. and Gross,M. | Effect of black tea intake on blood cholesterol concentrations in individuals with mild hypercholesterolemia: a diet-controlled randomized trial | J Acad Nutr Diet. 2015 Feb;115(2):264-71.e2. | 25266246 |
| 200 | Tzounis,X. and Rodriguez-Mateos,A. and Vulevic,J. and Gibson,G.R. and Kwik-Uribe,C. and Spencer,J.P. | Prebiotic evaluation of cocoa-derived flavanols in healthy humans by using a randomized, controlled, double-blind, crossover intervention study | Am J Clin Nutr. 2011 Jan;93(1):62-72. | 21068351 |
| 201 | Vafa,M. and Mohammadi,F. and Shidfar,F. and Sormaghi,M.S. and Heidari,I. and Golestan,B. and Amiri,F. | Effects of cinnamon consumption on glycemic status, lipid profile and body composition in type 2 diabetic patients | Int J Prev Med. 2012 Aug;3(8):531-6. | 22973482 |
| 202 | Vafa,M.R. and Haghighatjoo,E. and Shidfar,F. and Afshari,S. and Gohari,M.R. and Ziaee,A. | Effects of apple consumption on lipid profile of hyperlipidemic and overweight men | Int J Prev Med. 2011 Apr;2(2):94-100. | 21603015 |
| 203 | Vaisman,N. and Niv,E. | Daily consumption of red grape cell powder in a dietary dose improves cardiovascular parameters: a double blind, placebo-controlled, randomized study | Int J Food Sci Nutr. 2015 May;66(3):342-9. | 25666417 |
| 204 | van den Bogaard,B. and Draijer,R. and Westerhof,B.E. and van den Meiracker,A.H. and van Montfrans,G.A. and van den Born,B.J. | Effects on peripheral and central blood pressure of cocoa with natural or high-dose theobromine: a randomized, double-blind crossover trial | Hypertension. 2010 Nov;56(5):839-46. | 20823377 |
| 205 | van der Gaag,M.S. and van,Tol A. and Scheek,L.M. and James,R.W. and Urgert,R. and Schaafsma,G. and Hendriks,H.F. | Daily moderate alcohol consumption increases serum paraoxonase activity; a diet-controlled, randomised intervention study in middle-aged men | Atherosclerosis. 1999 Dec;147(2):405-10. | 10559527 |
| 206 | van der Gaag,M.S. and van,Tol A. and Vermunt,S.H. and Scheek,L.M. and Schaafsma,G. and Hendriks,H.F. | Alcohol consumption stimulates early steps in reverse cholesterol transport | J Lipid Res. 2001 Dec;42(12):2077-83. | 11734581 |
| 207 | van het Hof,K.H. and de Boer,H.S. and Wiseman,S.A. and Lien,N. and Westrate,J.A. and Tijburg,L.B. | Consumption of green or black tea does not increase resistance of low-density lipoprotein to oxidation in humans | Am J Clin Nutr. 1997 Nov;66(5):1125-32. | 9356529 |
| 208 | van Mierlo,L.A. and Zock,P.L. and van der Knaap,H.C. and Draijer,R. | Grape polyphenols do not affect vascular function in healthy men | J Nutr. 2010 Oct;140(10):1769-73. | 20702747 |
| 209 | Vauzour,D. and Houseman,E.J. and George,T.W. and Corona,G. and Garnotel,R. and Jackson,K.G. and Sellier,C. and Gillery,P. and Kennedy,O.B. and Lovegrove,J.A. and Spencer,J.P. | Moderate Champagne consumption promotes an acute improvement in acute endothelial-independent vascular function in healthy human volunteers | Br J Nutr. 2010 Apr;103(8):1168-78. | 19943984 |
| 210 | Vieira Senger,A.E. and Schwanke,C.H. and Gomes,I. and Valle Gottlieb,M.G. | Effect of green tea (Camellia sinensis) consumption on the components of metabolic syndrome in elderly | J Nutr Health Aging. 2012;16(9):738-42. | 23131813 |
| 211 | Vlachopoulos C, Aznaouridis K, Alexopoulos N, Economou E, Andreadou I, Stefanadis C. | Effect of dark chocolate on arterial function in healthy individuals. | Am J Hypertens. 2005 Jun;18(6):785-91. | 15925737 |
| 212 | Wainstein,J. and Stern,N. and Heller,S. and Boaz,M. | Dietary cinnamon supplementation and changes in systolic blood pressure in subjects with type 2 diabetes | J Med Food. 2011 Dec;14(12):1505-10. | 21861719 |
| 213 | Wan,Y. and Vinson,J.A. and Etherton,T.D. and Proch,J. and Lazarus,S.A. and Kris-Etherton,P.M. | Effects of cocoa powder and dark chocolate on LDL oxidative susceptibility and prostaglandin concentrations in humans | Am J Clin Nutr. 2001 Nov;74(5):596-602. | 11684527 |
| 214 | West,S.G. and McIntyre,M.D. and Piotrowski,M.J. and Poupin,N. and Miller,D.L. and Preston,A.G. and Wagner,P. and Groves,L.F. and Skulas-Ray,A.C. | Effects of dark chocolate and cocoa consumption on endothelial function and arterial stiffness in overweight adults | Br J Nutr. 2014 Feb;111(4):653-61. | 24274771 |
| 215 | Westphal,S. and Luley,C. | Flavanol-rich cocoa ameliorates lipemia-induced endothelial dysfunction | Heart Vessels. 2011 Sep;26(5):511-5. | 21140269 |
| 216 | Wickenberg,J. and Lindstedt,S. and Nilsson,J. and Hlebowicz,J. | Cassia cinnamon does not change the insulin sensitivity or the liver enzymes in subjects with impaired glucose tolerance | Nutr J. 2014 Sep 24;13:96. | 25249415 |
| 217 | Widlansky,M.E. and Hamburg,N.M. and Anter,E. and Holbrook,M. and Kahn,D.F. and Elliott,J.G. and Keaney,J.F.,Jr. and Vita,J.A. | Acute EGCG supplementation reverses endothelial dysfunction in patients with coronary artery disease | J Am Coll Nutr. 2007 Apr;26(2):95-102. | 17536120 |
| 218 | Wu,A.H. and Spicer,D. and Stanczyk,F.Z. and Tseng,C.C. and Yang,C.S. and Pike,M.C. | Effect of 2-month controlled green tea intervention on lipoprotein cholesterol, glucose, and hormone levels in healthy postmenopausal women | Cancer Prev Res (Phila). 2012 Mar;5(3):393-402. | 22246619 |
| 219 | Yang,T.Y. and Chou,J.I. and Ueng,K.C. and Chou,M.Y. and Yang,J.J. and Lin-Shiau,S.Y. and Hu,M.E. and Lin,J.K. | Weight reduction effect of Puerh tea in male patients with metabolic syndrome | Phytother Res. 2014 Jul;28(7):1096-101. | 24399768 |
| 220 | Yubero,N. and Sanz-Buenhombre,M. and Guadarrama,A. and Villanueva,S. and Carrion,J.M. and Larrarte,E. and Moro,C. | LDL cholesterol-lowering effects of grape extract used as a dietary supplement on healthy volunteers | Int J Food Sci Nutr. 2013 Jun;64(4):400-6. | 23249415 |
| 221 | Ziegenfuss,T.N. and Hofheins,J.E. and Mendel,R.W. and Landis,J. and Anderson,R.A. | Effects of a water-soluble cinnamon extract on body composition and features of the metabolic syndrome in pre-diabetic men and women | J Int Soc Sports Nutr. 2006 Dec 28;3:45-53. | 18500972 |
| 222 | Zunino,S.J. and Parelman,M.A. and Freytag,T.L. and Stephensen,C.B. and Kelley,D.S. and Mackey,B.E. and Woodhouse,L.R. and Bonnel,E.L. | Effects of dietary strawberry powder on blood lipids and inflammatory markers in obese human subjects | Br J Nutr. 2012 Sep;108(5):900-9. | 22068016 |
| 223 | Zunino,S.J. and Peerson,J.M. and Freytag,T.L. and Breksa,A.P. and Bonnel,E.L. and Woodhouse,L.R. and Storms,D.H. | Dietary grape powder increases IL-1beta and IL-6 production by lipopolysaccharide-activated monocytes and reduces plasma concentrations of large LDL and large LDL-cholesterol particles in obese humans | Br J Nutr. 2014 Aug 14;112(3):369-80. | 24832727 |

Reference List of Included Prospective Observational Studies

|  | **Authors** | **Title** | **Journal** | **Pubmed id** |
| --- | --- | --- | --- | --- |
| 1 | Arts,I.C. and Hollman,P.C. and Feskens,E.J. and Bueno de Mesquita,H.B. and Kromhout,D. | Catechin intake might explain the inverse relation between tea consumption and ischemic heart disease: the Zutphen Elderly Study | Am J Clin Nutr. 2001 Aug;74(2):227-32. | 11470725 |
| 2 | Arts,I.C. and Jacobs,D.R.,Jr. and Harnack,L.J. and Gross,M. and Folsom,A.R. | Dietary catechins in relation to coronary heart disease death among postmenopausal women | Epidemiology. 2001 Nov;12(6):668-75. | 11679795 |
| 3 | Baik,I. and Cho,N.H. and Kim,S.H. and Shin,C. | Dietary information improves cardiovascular disease risk prediction models | Eur J Clin Nutr. 2013 Jan;67(1):25-30. | 23149979 |
| 4 | Barrio-Lopez,M.T. and Bes-Rastrollo,M. and Sayon-Orea,C. and Garcia-Lopez,M. and Fernandez-Montero,A. and Gea,A. and Martinez-Gonzalez,M.A. | Different types of alcoholic beverages and incidence of metabolic syndrome and its components in a Mediterranean cohort | Clin Nutr. 2013 Oct;32(5):797-804. | 23305606 |
| 5 | Beulens,J.W. and Algra,A. and Soedamah-Muthu,S.S. and Visseren,F.L. and Grobbee,D.E. and van der Graaf,Y. and SMART Study Group | Alcohol consumption and risk of recurrent cardiovascular events and mortality in patients with clinically manifest vascular disease and diabetes mellitus: the Second Manifestations of ARTerial (SMART) disease study | Atherosclerosis. 2010 Sep;212(1):281-6. | 20537650 |
| 6 | Borgi,L. and Muraki,I. and Satija,A. and Willett,W.C. and Rimm,E.B. and Forman,J.P. | Fruit and Vegetable Consumption and the Incidence of Hypertension in Three Prospective Cohort Studies | Hypertension. 2016 Feb;67(2):288-93. | 26644239 |
| 7 | Cassidy A and Mukamal KJ and Liu L and Franz M and Eliassen AH and Rimm EB | High anthocyanin intake is associated with a reduced risk of myocardial infarction in young and middle-aged women. | Circulation. 2013 Jan 15;127(2):188-96. | 23319811 |
| 8 | Cassidy,A. and O'Reilly,E.J. and Kay,C. and Sampson,L. and Franz,M. and Forman,J.P. and Curhan,G. and Rimm,E.B. | Habitual intake of flavonoid subclasses and incident hypertension in adults | Am J Clin Nutr. 2011 Feb;93(2):338-47. | 21106916 |
| 9 | Hamer,M. and Witte,D.R. and Mosdol,A. and Marmot,M.G. and Brunner,E.J. | Prospective study of coffee and tea consumption in relation to risk of type 2 diabetes mellitus among men and women: the Whitehall II study | Br J Nutr. 2008 Nov;100(5):1046-53. | 18315891 |
| 10 | Hansen,L. and Dragsted,L.O. and Olsen,A. and Christensen,J. and Tjonneland,A. and Schmidt,E.B. and Overvad,K. | Fruit and vegetable intake and risk of acute coronary syndrome | Br J Nutr. 2010 Jul;104(2):248-55. | 20178672 |
| 11 | Hayashino,Y. and Fukuhara,S. and Okamura,T. and Tanaka,T. and Ueshima,H. and HIPOP-OHP Research Group | High oolong tea consumption predicts future risk of diabetes among Japanese male workers: a prospective cohort study | Diabet Med. 2011 Jul;28(7):805-10. | 21244473 |
| 12 | Ivey,K.L. and Lewis,J.R. and Prince,R.L. and Hodgson,J.M. | Tea and non-tea flavonol intakes in relation to atherosclerotic vascular disease mortality in older women | Br J Nutr. 2013 Nov 14;110(9):1648-55. | 23628082 |
| 13 | Jacques,P.F. and Cassidy,A. and Rogers,G. and Peterson,J.J. and Meigs,J.B. and Dwyer,J.T. | Higher dietary flavonol intake is associated with lower incidence of type 2 diabetes | J Nutr. 2013 Sep;143(9):1474-80. | 23902957 |
| 14 | Keli,S.O. and Hertog,M.G. and Feskens,E.J. and Kromhout,D. | Dietary flavonoids, antioxidant vitamins, and incidence of stroke: the Zutphen study | Arch Intern Med. 1996 Mar 25;156(6):637-42. | 8629875 |
| 15 | Kokubo,Y. and Iso,H. and Saito,I. and Yamagishi,K. and Yatsuya,H. and Ishihara,J. and Inoue,M. and Tsugane,S. | The impact of green tea and coffee consumption on the reduced risk of stroke incidence in Japanese population: the Japan public health center-based study cohort | Stroke. 2013 May;44(5):1369-74. | 23493733 |
| 16 | Kwok,C.S. and Boekholdt,S.M. and Lentjes,M.A. and Loke,Y.K. and Luben,R.N. and Yeong,J.K. and Wareham,N.J. and Myint,P.K. and Khaw,K.T. | Habitual chocolate consumption and risk of cardiovascular disease among healthy men and women | Heart. 2015 Aug;101(16):1279-87. | 26076934 |
| 17 | Lajous,M. and Rossignol,E. and Fagherazzi,G. and Perquier,F. and Scalbert,A. and Clavel-Chapelon,F. and Boutron-Ruault,M.C. | Flavonoid intake and incident hypertension in women | Am J Clin Nutr. 2016 Apr;103(4):1091-8. | 26936332 |
| 18 | Larsson,S.C. and Virtamo,J. and Wolk,A. | Chocolate consumption and risk of stroke: a prospective cohort of men and meta-analysis | Neurology. 2012 Sep 18;79(12):1223-9. | 22933736 |
| 19 | Larsson,S.C. and Virtamo,J. and Wolk,A. | Black tea consumption and risk of stroke in women and men | Ann Epidemiol. 2013 Mar;23(3):157-60. | 23295000 |
| 20 | Matsumoto,C. and Petrone,A.B. and Sesso,H.D. and Gaziano,J.M. and Djousse,L. | Chocolate consumption and risk of diabetes mellitus in the Physicians' Health Study | Am J Clin Nutr. 2015 Feb;101(2):362-7. | 25646334 |
| 21 | McCullough,M.L. and Peterson,J.J. and Patel,R. and Jacques,P.F. and Shah,R. and Dwyer,J.T. | Flavonoid intake and cardiovascular disease mortality in a prospective cohort of US adults | Am J Clin Nutr. 2012 Feb;95(2):454-64. | 22218162 |
| 22 | Mostofsky,E. and Levitan,E.B. and Wolk,A. and Mittleman,M.A. | Chocolate intake and incidence of heart failure: a population-based prospective study of middle-aged and elderly women | Circ Heart Fail. 2010 Sep;3(5):612-6. | 20713904 |
| 23 | Muraki I and Imamura F and Manson JE and Hu FB and Willett WC and van Dam RM and Sun Q | Fruit consumption and risk of type 2 diabetes: results from three prospective longitudinal cohort studies. | BMJ. 2013 Aug 28;347:f5001. | 23990623 |
| 24 | Mursu,J. and Voutilainen,S. and Nurmi,T. and Tuomainen,T.P. and Kurl,S. and Salonen,J.T. | Flavonoid intake and the risk of ischaemic stroke and CVD mortality in middle-aged Finnish men: the Kuopio Ischaemic Heart Disease Risk Factor Study | Br J Nutr. 2008 Oct;100(4):890-5. Apr 1. | 18377681 |
| 25 | Nakachi,K. and Matsuyama,S. and Miyake,S. and Suganuma,M. and Imai,K. | Preventive effects of drinking green tea on cancer and cardiovascular disease: epidemiological evidence for multiple targeting prevention | Biofactors. 2000;13(1-4):49-54. | 11237198 |
| 26 | Nunez-Cordoba,J.M. and Martinez-Gonzalez,M.A. and Bes-Rastrollo,M. and Toledo,E. and Beunza,J.J. and Alonso,A. | Alcohol consumption and the incidence of hypertension in a Mediterranean cohort: the SUN study | Rev Esp Cardiol. 2009 Jun;62(6):633-41. | 19480759 |
| 27 | Odegaard,A.O. and Koh,W.P. and Yuan,J.M. and Pereira,M.A. | Beverage habits and mortality in Chinese adults | J Nutr. 2015 Mar;145(3):595-604. | 25733477 |
| 28 | Odegaard,A.O. and Pereira,M.A. and Koh,W.P. and Arakawa,K. and Lee,H.P. and Yu,M.C. | Coffee, tea, and incident type 2 diabetes: the Singapore Chinese Health Study | Am J Clin Nutr. 2008 Oct;88(4):979-85. | 18842784 |
| 29 | Saito,E. and Inoue,M. and Sawada,N. and Shimazu,T. and Yamaji,T. and Iwasaki,M. and Sasazuki,S. and Noda,M. and Iso,H. and Tsugane,S. and JPHC Study Group | Association of green tea consumption with mortality due to all causes and major causes of death in a Japanese population: the Japan Public Health Center-based Prospective Study (JPHC Study) | Ann Epidemiol. 2015 Jul;25(7):512-518.e3. | 25900254 |
| 30 | Suzuki,E. and Yorifuji,T. and Takao,S. and Komatsu,H. and Sugiyama,M. and Ohta,T. and Ishikawa-Takata,K. and Doi,H. | Green tea consumption and mortality among Japanese elderly people: the prospective Shizuoka elderly cohort | Ann Epidemiol. 2009 Oct;19(10):732-9. | 19628408 |
| 31 | Tanabe,N. and Suzuki,H. and Aizawa,Y. and Seki,N. | Consumption of green and roasted teas and the risk of stroke incidence: results from the Tokamachi-Nakasato cohort study in Japan | Int J Epidemiol. 2008 Oct;37(5):1030-40. | 18832387 |
| 32 | Tresserra-Rimbau,A. and Rimm,E.B. and Medina-Remon,A. and Martinez-Gonzalez,M.A. and de la Torre,R. and Corella,D. and Salas-Salvado,J. and Gomez-Gracia,E. and Lapetra,J. and Aros,F. and Fiol,M. and Ros,E. and Serra-Majem,L. and Pinto,X. and Saez,G.T. and Basora,J. and Sorli,J.V. and Martinez,J.A. and Vinyoles,E. and Ruiz-Gutierrez,V. and Estruch,R. and Lamuela-Raventos,R.M. and PREDIMED,Study,I | Inverse association between habitual polyphenol intake and incidence of cardiovascular events in the PREDIMED study | Nutr Metab Cardiovasc Dis. 2014 Jun;24(6):639-47. | 24552647 |
| 33 | Vogiatzoglou,A. and Mulligan,A.A. and Bhaniani,A. and Lentjes,M.A. and McTaggart,A. and Luben,R.N. and Heiss,C. and Kelm,M. and Merx,M.W. and Spencer,J.P. and Schroeter,H. and Khaw,K.T. and Kuhnle,G.G. | Associations between flavan-3-ol intake and CVD risk in the Norfolk cohort of the European Prospective Investigation into Cancer (EPIC-Norfolk) | Free Radic Biol Med. 2015 Jul;84:1-10. | 25795512 |
| 34 | Wedick,N.M. and Pan,A. and Cassidy,A. and Rimm,E.B. and Sampson,L. and Rosner,B. and Willett,W. and Hu,F.B. and Sun,Q. and van Dam,R.M. | Dietary flavonoid intakes and risk of type 2 diabetes in US men and women | Am J Clin Nutr. 2012 Apr;95(4):925-33. | 22357723 |
